# North Pacific meltwater weakens the Atlantic Meridional Overturning Circulation and preconditions Heinrich Stadial 1

**DOI:** 10.1038/s41467-026-75199-y

**Published:** 2026-07-04

**Authors:** Chijun Sun, Jiang Zhu, Bette L. Otto-Bliesner, Esther C. Brady, Sophia I. Macarewich

**Affiliations:** 1 Department of Earth and Planetary Sciences, University of California Davis, Davis, CA USA; 2 Climate & Global Dynamics, NSF National Center for Atmospheric Research, Boulder, CO USA

**Keywords:** Palaeoceanography, Palaeoclimate, Physical oceanography

## Abstract

Heinrich Stadials provide insights into a potential future weakening of the Atlantic Meridional Overturning Circulation (AMOC), yet the mechanisms driving AMOC weakening during these intervals remain elusive. While Heinrich Stadials are associated with iceberg-discharge events over the North Atlantic (i.e., Heinrich events), AMOC weakening is known to precede Heinrich events. Here we run freshwater hosing experiments to show that contemporaneous Northeast Pacific iceberg-discharge events (i.e., Siku events), which consistently occur at the onsets of Heinrich Stadials, could trigger AMOC weakening by transporting freshwater to the North Atlantic deepwater formation regions. This initial weakening may subsequently be amplified by further meltwater release from the British and Laurentide Ice Sheets, induced by stepwise subsurface warming in the northeastern and northern North Atlantic. Our findings suggest that Siku events may have played a critical role in preconditioning the North Atlantic for Heinrich events, underscoring the interconnected nature of the global ocean–cryosphere system.

## Abstract (web summary)

This study shows that Cordilleran ice discharge to the North Pacific may have triggered AMOC weakening during Heinrich Stadials, potentially destabilizing the European and Laurentide Ice Sheets and leading to Heinrich events.

## Introduction

The Atlantic Meridional Overturning Circulation (AMOC)—an ocean current system that accounts for over 70% of global cross-equatorial oceanic heat transport^1^—is projected to weaken in response to future warming^2^. Weakening of the AMOC is expected to have far-reaching impacts on global climate through redistribution of heat and energy^2,3^, yet the unclear rate and magnitude of AMOC weakening is likely a source for large uncertainties in projected future climate change^4^. How AMOC responds to external forcings and how a potential weakening of AMOC would impact the climate system needs to be studied and validated with observations. However, instrumental records of AMOC variability are too short, and the changes are too small to discern externally driven changes from internal variability^5,6^.

Heinrich events, which occurred during prolonged Greenland cold intervals known as Heinrich stadials, are associated with significant AMOC weakening as indicated by proxy records^7–9^, making them a target for understanding the causes, mechanisms, and impacts of AMOC weakening^3^. These Heinrich events are characterized by distinct ice-rafted debris layers in the so-called Ruddiman Belt across the subpolar North Atlantic, indicative of massive iceberg discharges from the Laurentide Ice Sheet^10^. Melting of these icebergs was initially hypothesized to be the triggering mechanism for AMOC weakening during Heinrich stadials, where the meltwater could reduce surface water buoyancy and weaken North Atlantic Deep Water (NADW) formation^11^. However, updated chronological analyses show that Heinrich events lag AMOC weakening by at least 1000 years^12^, indicating that Heinrich events could not possibly be the trigger for AMOC weakening during Heinrich stadials, but nevertheless play an important role in further weakening the AMOC during the peak of Heinrich stadials^13^. A full understanding of AMOC’s behaviors during Heinrich stadials hinges on better resolving the nature of Heinrich events and their relationship to AMOC variability.

Various hypotheses have been proposed for Heinrich events. An early hypothesis argues that Heinrich events are driven by a “binge-purge” mechanism associated with the internal instability of ice sheets^14^, but it is criticized for failing to explain the phase-locking between Heinrich events and the cold phases of the Dansgaard-Oschger (DO) cycles^15^. While a recent study finds that applying DO-like atmospheric forcings to an ice sheet model could largely reproduce the phase-locking pattern^15^, this mechanism remains to be tested with more realistic transient climate forcings. Recent theories emphasize the roles of the ocean. Proxy records from the subpolar North Atlantic suggest significant subsurface warming preceding the initiation of Heinrich events^16,17^, leading to the hypothesis that the subsurface warming could have disintegrated Laurentide ice shelves or ice cliffs, thereby increasing iceberg calving^16–19^, similar to the observed retreat of the West Antarctica ice today. Furthermore, Bassis et al. ^19^ incorporated DO-related isostatic adjustments, which regulate the exposure of the Laurentide Ice Sheet marine terminus to subsurface ocean warming, successfully simulating all Heinrich events of the last glacial period in the model. Thus, the precursory subsurface warming provides a viable mechanism for Heinrich events. The subsurface warming has been hypothesized to be driven by the early AMOC weakening, which reduces the downward mixing of cold surface waters, allowing heat to accumulate in the subsurface^20^. Yet, the mechanisms driving AMOC weakening prior to Heinrich events remain unclear. Modeling studies suggest that substantial AMOC weakening can result from variations in orbital forcing, CO₂, and ice sheet configurations^21–25^. Sedimentary records have also suggested that meltwater from the European Ice Sheets could have contributed to AMOC weakening^10,26–29^, however, this hypothesis remains debated due to the lack of consistent correspondence between European Ice Sheet melting and Heinrich events in proxy records^30,31^. A consistent framework explaining the coevolution of AMOC, ice sheet dynamics, and climate during Heinrich stadials is still lacking.

**Fig. 1:**
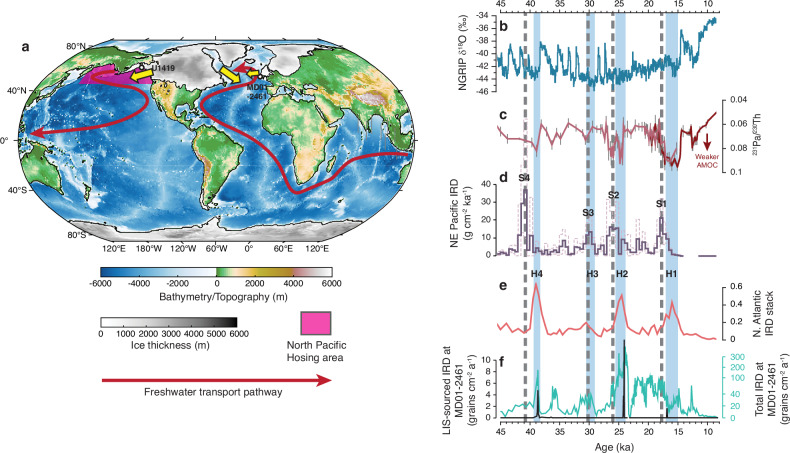
AMOC strength and Greenland climate in relation to North Pacific and North Atlantic meltwater events over the past 45 thousand years. **a** Boundary conditions at 19 thousand years ago (ka). Bathymetry, topography, and ice sheets are from the ICE-6G_C reconstruction^58^. Pink area indicates the hosing region in the North Pacific hosing experiments reported in this study. The red arrow indicates the pathway for the transport of freshwater from the North Pacific to the Atlantic. Yellow arrows indicate meltwater events associated with the Cordilleran, Laurentide, and British-Irish Ice Sheets. The map was generated using the m_map mapping package for MATLAB^70^ and the Climate Data Toolbox for MATLAB^71^. **b** North Greenland Ice Core Project (NGRIP) δ^18^O record^72^. **c** Bermuda Rise ^231^Pa/^230^Th records showing changes in the Atlantic Meridional Overturning Circulation (AMOC)^7–9^. **d** Ice-rafted debris (IRD) record from U1419 in the Northeast Pacific^32^. Dashed lines indicate ± 1 σ. **e** North Atlantic IRD stack^73^. **f** IRD records from MD2461 in the Northeast Atlantic showing total IRD predominantly from the British-Irish Ice Sheet (green curve) and IRD of dolomitic carbonate from the Laurentide Ice Sheet (black curve)^31^. Site locations for U1419 and MD01-2461 are shown in panel (**a**). The blue bars denote Heinrich Events 1–4 (H1–H4); the gray dashed straight lines denote Siku Events 1-4 (S1–S4).

More recently, sedimentary cores from the Northeast Pacific reveal that episodic iceberg discharges from the Cordilleran Ice Sheet, namely “Siku events”, consistently lead Atlantic Heinrich events by ~ 1400 years^32^ and broadly coincide with the onsets of Heinrich stadials and early AMOC weakening (Fig. 1). Previous studies of these Siku events mostly focused on their regional impacts in the North Pacific^32,33^; however, little is known about how these events may have affected the global ocean and climate. Motivated by the intriguing phasing relationship, we hypothesize that Siku events could initiate AMOC weakening, which would subsequently drive North Atlantic subsurface warming and trigger Heinrich events. To test this hypothesis, we run a set of climate model experiments exploring the impacts of Siku events. We focus on Siku Event 1 (S1; 19-16 ka), which is the most recent Siku event, occurring at the onset of Heinrich Stadial 1 (HS1; 19-15 ka) immediately after the Last Glacial Maximum (LGM; 22-19 ka), with abundant paleoclimate records and precise chronology offering well-constrained boundary conditions and global climate responses. In the first set of simulations, we apply a freshwater flux of 0.1, 0.2, or 0.5 Sverdrup (Sv; 1 Sv = 10^6 ^m^3 ^s^‒1^) to the Northeast Pacific (Supplementary Fig. S1) for 400 years under the glacial boundary conditions (hereafter “NP01”, “NP02”, and “NP05”) using the fully coupled Community Earth System Model version 1.3^34,35^ (see “Methods”). These forcing scenarios range from a realistic estimate (i.e., NP01) to an exaggerated value (i.e., NP05). An exaggeration of freshwater forcing is often needed to account for the known AMOC over-stability in current-generation general circulation models (see “Methods”). Furthermore, this range of forcing intensities allows us to assess the robustness and sensitivity of AMOC’s response to the North Pacific meltwater event. Building upon these results, we perform additional simulations to investigate how S1 meltwater, in conjunction with European Ice Sheet melt, may have impacted the North Atlantic cryosphere leading up to Heinrich Event 1 (H1; 15–17 ka; see “Methods”).

## Results and discussion

### AMOC weakening at the onset of HS1 driven by Cordilleran meltwater

**Fig. 2:**
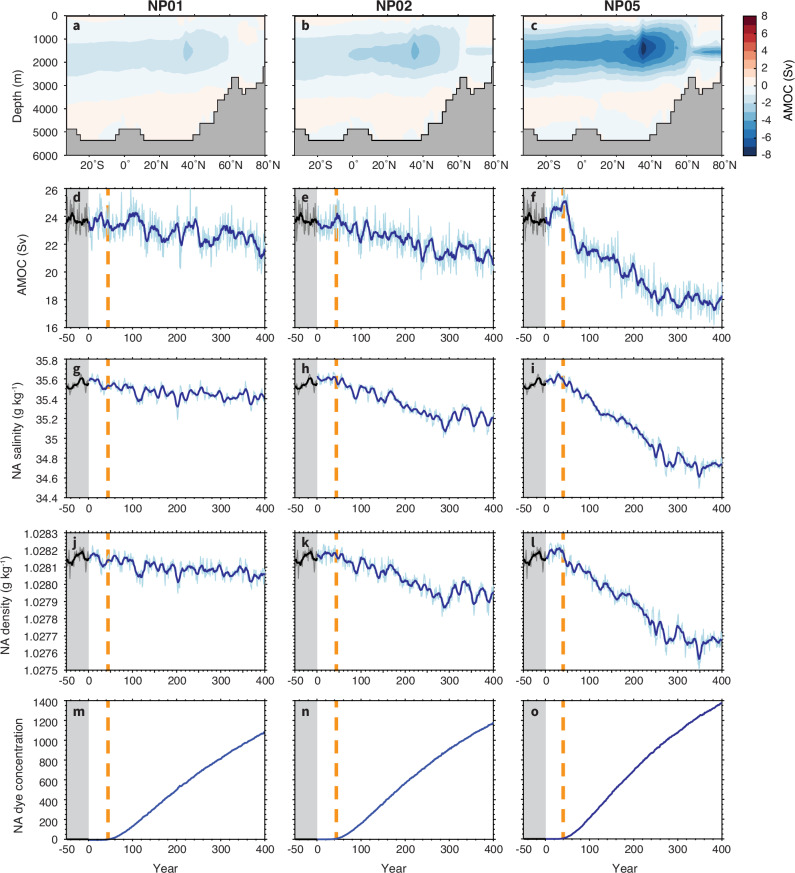
AMOC weakening in association with freshwater transport from the North Pacific. **a**–**c** Changes in AMOC strength in (**a**) NP01, (**b**) NP02, and (**c**) NP05 relative to the 19 ka control. **d**–**f** Evolution of AMOC strength (defined as the maximum stream function between 10 and 60˚N) in (**d**) NP01, (**e**) NP02, and (**f**) NP05. **g**–**i** Evolution of upper water (top 100 m) salinity in the North Atlantic (NA; 45–60°N) in (**g**) NP01, (**h**) NP02, and (**i**) NP05. **j**–**l** Evolution of upper water density in the North Atlantic in (**j**) NP01, (**k**) NP02, and (**l**) NP05. **m**–**o** Dye tracer concentration in the North Atlantic upper water, indicative of water mass transport from the North Pacific in (**m**) NP01, (**n**) NP02, and (**o**) NP05. In panels (**d**–**o**), the thin light-colored lines denote annual mean values, and the thick dark-colored lines denote 10-year moving average values. The gray bars indicate the last 50 years of the 19 ka control simulation. The orange dashed lines indicate the first appearance of the dye tracer in the North Atlantic.

In our experiments, the applied Northeast Pacific freshwater forcing weakens the AMOC by 7% (1.8 Sv), 11% (2.6 Sv), and 25% (6.0 Sv) in NP01, NP02, and NP05, respectively (Fig. 2a–f), exceeding AMOC natural variability in the model (Supplementary Fig. S2). The AMOC weakening is associated with a decrease in salinity and density of the North Atlantic surface waters (Supplementary Fig. 2g–l), which weakens NADW formation and thereby weakens the AMOC (Supplementary Fig. S3). The simultaneous arrival of the passive dye tracer (Fig. 2m–o), which we added to the North Pacific hosing region to track the transport and mixing of the water mass, indicates that it is the transport of the North Pacific freshwater to the North Atlantic that causes the AMOC weakening and North Atlantic freshening. Since the Bering Strait is closed at 19 ka, the freshwater transport takes a westward Pacific-Indian-Atlantic surface pathway: the freshwater is quickly mixed and transported to the tropical Pacific by the subtropical gyre, entering the Indian Ocean through the Indonesian Throughflow by model year 10 (Supplementary Fig. S4, see Supplementary Information). By model year 15, the water mass enters the South Atlantic via the Agulhas Leakage. In the Atlantic Ocean basin, the freshwater travels northward, eventually reaching the North Atlantic in model year 40-50. A weaker AMOC is amplified by a positive feedback associated with expanded sea ice, which increases meltwater fluxes when sea ice melts during summer and freshens the North Atlantic (Supplementary Figs. S5, 6). In addition, although atmospheric processes may also affect salinity by altering regional hydroclimate, their influence is likely secondary. Cooling over the North Atlantic reduces precipitation and runoff, which is largely balanced by reduced evaporation (Supplementary Fig. S7) and dwarfed by those from the meltwater flux (Supplementary Fig. S5).

The simulated pathway and rate of Pacific-to-Atlantic freshwater transport in our model are consistent with those based on a high-resolution eddy-permitting ocean model^33^ and modern observations synthesized by the “Estimating the Circulation and Climate of the Ocean” (ECCOv4) product^36,37^, which estimate transit times of 5–10 years for North Pacific waters to reach the tropical Pacific^33^ and ~30 years for waters advected through the Indonesian Throughflow to reach the tropical Atlantic^36^. Therefore, the freshwater transport mechanism proposed here, while novel in the context of deglacial climate change, is physically realistic and insensitive to the forcing intensity. Today, this pathway accounts for over 35% of the total cross-equatorial water mass in the upper branch of AMOC^36^. Quantitative reconstructions of Agulhas Leakage strength suggest that this ocean current importing Indian Ocean waters to the Atlantic remains active during glacial periods^38,39^, and thus, it is reasonable to infer that Siku meltwater could travel across the globe to the North Atlantic to impact the AMOC.

**Fig. 3:**
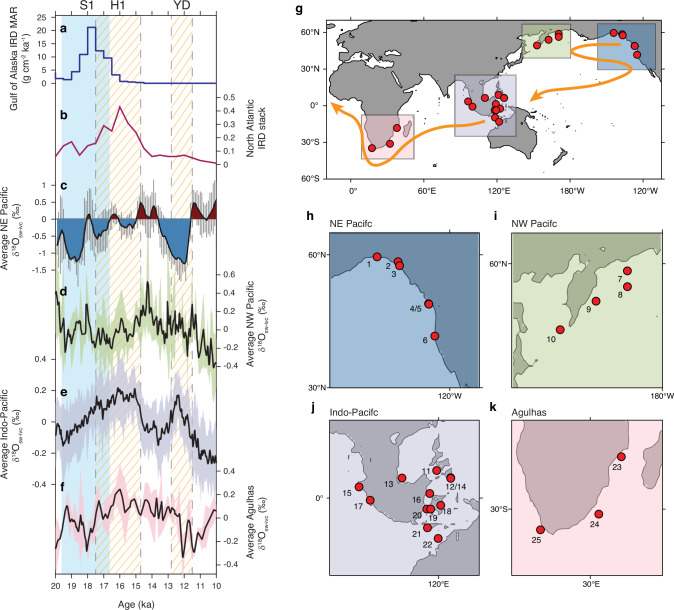
Proxy evidence for surface freshening along the Pacific-Indian-Atlantic freshwater transport pathway during S1. **a** IRD record from U1419 in the Northeast Pacific^32^. **b** North Atlantic IRD stack^73^. **c** Average of the δ^18^O_sw-ivc_ records from the Northeast (NE) Pacific synthesized by Praetorius et al.^33^. **d** Average of the δ^18^O_sw-ivc_ records from the Northwest (NW) Pacific. **e** Average of the δ^18^O_sw-ivc_ records from the Indo-Pacific Warm Pool region. **f** Average of the δ^18^O_sw-ivc_ records from the Agulhas Leakage region. To calculate the averages in (**d**–**f**), each individual record is linearly interpolated to a 20-year resolution and the mean deglacial values (20–10 ka) is removed prior to averaging. The color shadings denote ± σ, with the color corresponding to highlighted regions in panels (**g**–**k**). The vertical blue bar across the panels on the left highlights S1; the orange hatched bars highlight H1 and Younger Dryas. **g** Map showing the locations of all δ^18^O_sw-ivc_ records included. Colored boxes denote individual subregions. The orange arrow indicates the freshwater transport pathway. **h**–**k** Zoomed-in maps showing the locations of δ^18^O_sw-ivc_ records from (**h**) the Northeast Pacific; (**i**) the Northwest Pacific; (**j**) the Indo-Pacific Warm Pool; and (**k**) the Agulhas Leakage regions. Numbers adjacent to each site correspond to the site information listed in Supplementary Table S2. Maps were generated using the m_map mapping package for MATLAB^70^.

The proposed meltwater transport pathway is also supported by proxy evidence from multiple sites across the Pacific, Indonesian Throughflow, and south of Africa. Since ice sheet meltwater is isotopically lighter than seawater, planktic foraminiferal oxygen isotope data corrected for temperature and global ice volume changes (δ¹⁸O_sw-ivc_) can be used to track freshwater transport in the upper ocean. In the Northeast Pacific, an average of δ¹⁸O_sw-ivc_ records shows a negative shift of up to − 1.2‰ around 19 ka^33^ (Fig. 3c, h), consistent with the discharge of isotopically light meltwater from the Cordilleran Ice Sheet. The apparent chronological mismatch between the peak isotope shift and IRD accumulation likely reflects challenges associated with the dating of marine sediments, as well as inherently large uncertainties associated with the δ¹⁸O_sw-ivc_ proxy system and its interpretation, including those due to Mg/Ca-SST quantification, ice volume estimation, and variability in ocean current, hydroclimate, and meltwater δ¹⁸O. A negative isotope shift during S1 is also observed at key downstream locations, including the Northwest Pacific (Fig. 3d, i), the Indonesian Throughflow region (Fig. 3e, j), and along the Agulhas Current south of Africa (Fig. 3f, k). Although we cannot rule out the influence of other potential mechanisms in driving these isotope changes, given the proxy uncertainties, the broadly synchronous isotope depletion between 19 and 17 ka is consistent with the meltwater transport mechanism proposed in this study.

**Fig. 4:**
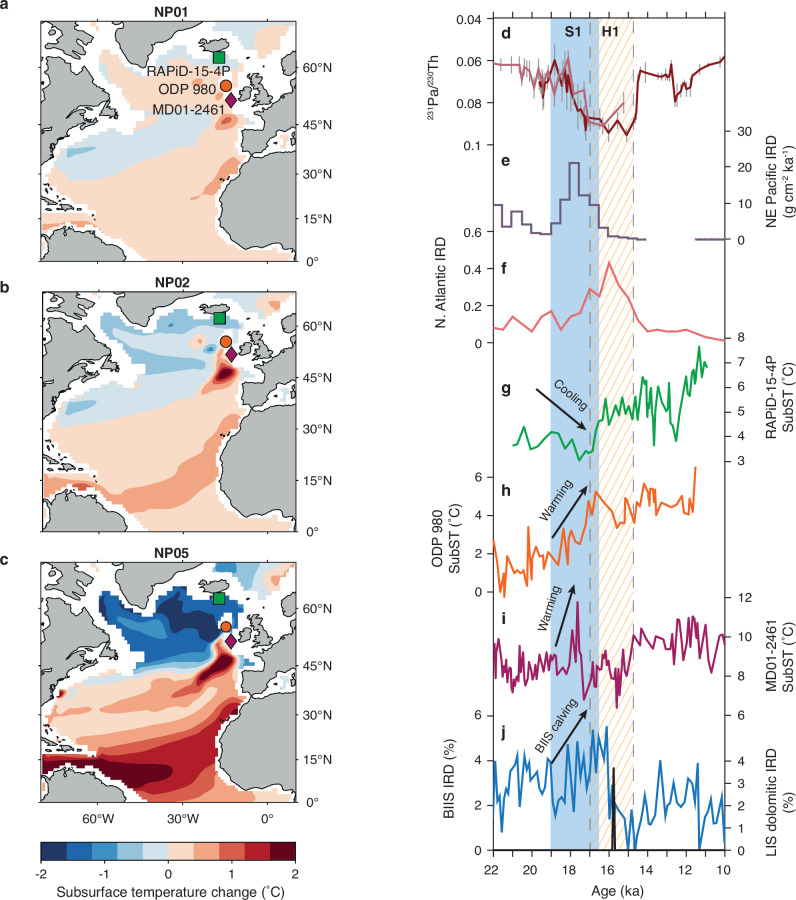
Simulated North Atlantic subsurface response consistent with proxy records. **a**–**c** Changes in annual mean North Atlantic subsurface temperature in (**a**) NP01, (**b**) NP02, and (**c**) NP05. Mean values of 300–700 m depth are shown. Green square, orange circle, and purple diamond denote the locations of sites RAPiD-15-4P, ODP 980, and MD01-2461, where subsurface temperature reconstructions are available and shown on the right. Maps were generated using the m_map mapping package for MATLAB^70^ and the Climate Data Toolbox for MATLAB^71^. **d** ^231^Pa/^230^Th-based AMOC reconstruction from the Bermuda Rise^7–9^. **e** IRD record from U1419 in the Northeast Pacific^32^. **f** North Atlantic IRD stack^73^. **g** Subsurface temperature (SubST) record from RAPiD-15-4P^74^. **h** SubST record from ODP 980^75^. **i** SubST from MD01-2461^40^. Subsurface temperatures in (**g**–**i**) are based on Mg/Ca of subsurface dwelling foraminfera *N. Pachyderma* (sinistral). The black arrows denote the trends of subsurface temperature change. **j** IRD record form MD01-2461^40^, with blue showing the percentage of BIIS-sourced grains and a single black spike marking Laurentide-derived dolomitic IRD. For data from MD01-2461, Max et al.^17^’s updated age model is used.

The robust responses in our North Pacific hosing simulations, as well as model-data agreement, suggest that the Pacific meltwater may have driven AMOC weakening at the onset of HS1. This mechanism does not require freshwater forcings directly from the North Atlantic (e.g., meltwater from the Laurentide or European Ice Sheets). Although the AMOC is not fully shut down and the changes are relatively small in our S1 simulations, it is consistent with the reconstructed AMOC evolution showing that AMOC weakening was gradual at the onset of HS1 (Fig. 4d)^7,8^.

### AMOC weakening amplified by BIIS retreat and Heinrich Event 1

An AMOC weakening caused by the Pacific meltwater from S1 can be amplified by local processes in the northern North Atlantic, through melting of ice shelves of the Laurentide Ice Sheet in the west^16,18^ or the European Ice Sheets in the east^26,27^. We find that this initial AMOC weakening and associated changes in ocean surface and subsurface temperatures may trigger a chain response, causing sequential freshwater discharges from the British Irish Ice Sheet (BIIS) and Laurentide ice shelves and leading to an AMOC collapse.

Our North Pacific hosing simulations show a pronounced subsurface warming in the mid-latitude Northeast Atlantic off the Irish southwest coast (Fig. 4a–c). This warming is associated with a weakened North Atlantic subpolar gyre and a strengthened and northward-shifted subtropical gyre, which causes accumulation of heat in the eastern basin along the North Atlantic Current (Supplementary Figs. S8, 9; see Supplementary Information). However, in contrast to the previous hypothesis that an initial AMOC weakening warms the northern North Atlantic subsurface and triggers a collapse of the Laurentide ice shelf^16,18^, this S1-driven subsurface warming does not extend into the northern North Atlantic or the Labrador Sea (Fig. 4a–c). Instead, cooling prevails in both the surface and subsurface of the northern North Atlantic due to reduced northward heat transport by a weaker but not collapsed AMOC (Fig. 4 and Supplementary Fig. S6), which would stabilize the Laurentide Ice Sheet. In addition, our ice sheet surface mass balance calculation using a Positive Degree Day (PDD) model (see “Methods”) suggests that the associated atmospheric responses, primarily due to surface cooling, would also likely promote expansion, rather than retreat, of the Laurentide Ice Sheet over the Labrador Sea (Supplementary Fig. S10). Together, these results reject the hypothesis that S1 meltwater could directly trigger Laurentide ice shelf melting by itself. Nevertheless, the simulated pattern of Northeast Atlantic subsurface temperature response agrees with proxy records from the region: two sites off the Irish west coast show abrupt subsurface warming (Fig. 4h, i), whereas a northern North Atlantic site registers cooling during S1 (Fig. 4g). This model-data agreement suggests that our simulations capture the key features of subsurface temperature changes in the North Atlantic during S1.

We propose that the Northeast Atlantic subsurface warming, in conjunction with surface cooling, could have promoted the retreat of the marine-terminated BIIS during S1 as an intermediate step toward H1. At the surface, the PDD surface mass balance model shows a net positive over the western British Isles in all three simulations (Supplementary Fig. S10) due to cooling and increased snowfall (in NP02 and NP05), which would promote seaward advances of the BIIS (Supplementary Fig. S10). Meanwhile, the subsurface warming could destabilize the BIIS from its marine grounding line. The combination of surface growth and basal melt provides a mechanism for enhanced iceberg calving that could explain the observed increase in BIIS-sourced IRD during S1 (Fig. 4j)^26–28,40^. Although the BIIS had only a limited ocean interface^41^, making basal melt alone an unlikely major freshwater source, debuttressing at its grounding line could have triggered larger-scale instability and accelerated retreat. This is consistent with sedimentary records showing increased meltwater discharge from the European Ice Sheets via the Channel River^29^, and paleoglaciological evidence of rapid BIIS retreat at ~ 19 ka^41^.

**Fig. 5:**
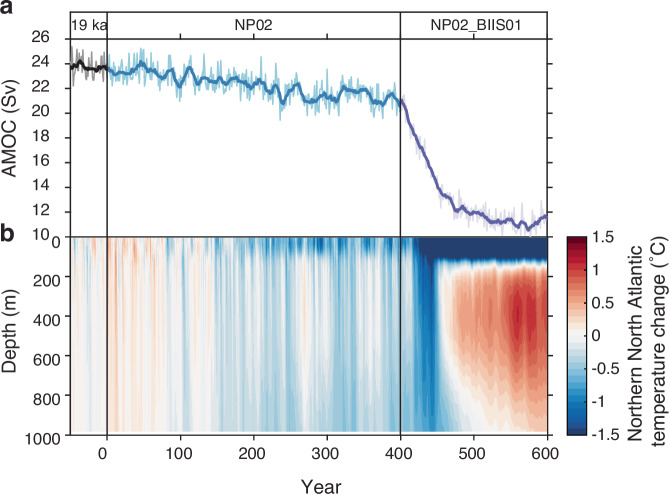
Responses of AMOC and northern North Atlantic temperature to North Pacific and BIIS meltwater events showing a strong subsurface warming towards the end. **a** Evolution of AMOC strength in the 19 ka control, NP02, and NP02_BIIS01 simulations. The thin light-colored lines represent annual mean values, and the thick dark-colored lines represent 10-year moving average values. **b** Hovmöller diagram of temperature anomalies in the northern North Atlantic (north of 45°N, excluding the Greenland-Iceland-Norwegian Seas) for the 19 ka control, NP02, and NP02_BIIS01 simulations.

Because BIIS-sourced IRD layers consistently underly the Laurentide-sourced Heinrich IRD layers, BIIS retreat has been hypothesized to be the precursor to Heinrich events^26–29^. Mechanistically, BIIS meltwater could lead to a Heinrich event by further weakening NADW formation and AMOC, which in turn would drive further subsurface ocean warming to destabilize the Laurentide ice shelf^16–18^. The BIIS is estimated to have discharged ~ 2 m sea-level equivalent (m s.l.e) of meltwater around 19 ka and 17 ka according to a transient ice sheet modeling study^42^, and this melting could have been accelerated by the S1-driven subsurface warming. The proximity of BIIS to the NADW formation region means that even a small freshwater forcing from the Northeast Atlantic could disproportionally weaken the AMOC^43^. We ran additional hosing experiments, in which a freshwater flux of 0.05 or 0.1 Sv is imposed along the west coast of Ireland under the boundary conditions after NP02 (“NP02_BIIS005” and “NP02_BIIS01”; see Methods), to better understand the impact of the BIIS meltwater event following S1. Our results show that while the 0.05 SV scenario (i.e., NP02_BIIS005) does not show warming in the northern North Atlantic, the one forced with 0.1 Sv shows substantial further AMOC weakening, causing subsurface warming in the northern North Atlantic in about 50 years (Fig. 5b and Supplementary Fig. S11). In addition, both simulations show subsurface warming in the Nordic Seas, which could potentially drive more freshwater release from the Fennoscandian Ice Sheet (FIS), further weakening the AMOC (Supplementary Fig. S11). This is consistent with sedimentary evidence for increased FIS meltwater release during S1^29,44^. At the Labrador Sea region where the Laurentide Ice Sheet grounds, the subsurface temperature is ~ 1.4 °C warmer than in the LGM control (Fig. 5 and Supplementary Fig. S11). Marcott et al.^16^ used an ice shelf model to show that a Labrador Sea subsurface warming of similar magnitude would greatly accelerate the rate of basal melt of the Laurentide Ice Sheet, and at this rate, the ice shelf is estimated to collapse in ~ 1000 years. Therefore, our results support that H1 could be triggered by S1, with the BIIS meltwater discharge as an important intermediate step to drive subsurface warming in the northern North Atlantic.

Furthermore, the Siku-BIIS-Heinrich sequence may also reconcile a key limitation of the BIIS precursor hypothesis that not all BIIS meltwater events were followed by Heinrich events^30,31^. We note that every Heinrich-leading BIIS meltwater event was preceded by a Siku event (Fig. 1), implying that a preceding Siku event may be a necessary ingredient for BIIS meltwater to trigger a Heinrich event. To test this hypothesis, we run additional simulations to examine the effect of adding 0.1 Sv of BIIS meltwater under the 19 ka control conditions without preconditioning by the Siku meltwater (“ctrl_BIIS01; see “Methods”) and compared it to NP02_BIIS01. The results show that the AMOC is consistently ~ 2 Sv weaker in the simulation run under the NP02 initial conditions than in the one under the 19 ka control climate conditions (Supplementary Fig. S12). More importantly, these two runs show strikingly different northern North Atlantic subsurface temperature responses: NP02_BIIS01 produces subsurface warming in the northern North Atlantic after year ~ 50, whereas ctrl_BIIS01 remains anomalously cooler for an extended period until year 100 (Supplementary Information; Supplementary Fig. S13), suggesting that the preconditioning of the North Atlantic by S1 meltwater could drastically accelerate the basal melting of the Laurentide ice shelf. The different subsurface temperature responses to AMOC weakening stem from the competing impacts of warming associated with suppressed vertical mixing and cooling due to reduced poleward oceanic heat transport^20^. In NP02_BIIS01, Siku meltwater has preconditioned the North Atlantic with a weaker initial AMOC state and more buoyant surface waters, which, combined with BIIS meltwater, makes it easier to cross the threshold where subsurface warming would prevail. Subsequently, this subsurface warming would drive iceberg discharge from the Laurentide Ice Sheet, leading to H1. As such, the role of S1 meltwater during H1 may involve both initiating BIIS meltwater release via a weakened AMOC and its lingering freshwater effect that amplifies the impact of the BIIS precursor event.

### Summary and Implications

The interactions between AMOC, ice sheet dynamics, and climate during Heinrich stadials are long-standing paleoclimate questions that have important implications for our understanding of the Earth system. This study uses a climate model to demonstrate that Siku Event 1 could have initiated the weakening of the AMOC at the onset of Heinrich Stadial 1. This initial AMOC weakening could be subsequently amplified through triggering the BIIS meltwater release, progressively leading to basal melting of the Laurentide Ice Sheet and a collapse of the AMOC. The processes involved are consistent with both the timing and patterns of paleoclimate records, including ice-rafted debris in the North Pacific and the North Atlantic, ocean subsurface temperature variations, AMOC strength, and changes in seawater oxygen isotopes along the freshwater transport pathway. Together, our results provide a unifying framework that explains the sequential meltwater events from the Cordilleran, European, and Laurentide Ice Sheets during HS1.

This study does not consider the impacts of solar insolation, CO_2_, and ice sheet. Given that these forcings evolve gradually and slowly at the early HS1, they are unlikely to explain the AMOC weakening at the onset of HS1. Using the iTRACE factorized-forcing simulations, we find that the magnitude of AMOC weakening due to insolation, CO_2_, and ice sheet at 19 ka are considerably smaller than the response in our North Pacific freshwater hosing simulations (Fig. S2), highlighting an important role for S1 meltwater in weakening the AMOC at the onset of HS1. That said, all these mechanisms are not mutually exclusive. In particular, increasing summer insolation during the deglaciation may have enhanced ice sheet melting and freshwater input^10,45^, providing an additional freshwater forcing for AMOC weakening.

Although our study focuses on HS1, the recurring sequence of Siku events, BIIS meltwater discharge, and Heinrich events during at least HS2-4 according to available proxy records (Fig. 1) suggests that a similar mechanism may be plausible for these earlier events as well. However, we acknowledge that all Heinrich stadials are not the same, with each associated with distinct forcings and boundary conditions. For example, Farmer et al.^46^ present evidence for an open Bering Strait during HS4, which is different from our model setup with a closed Bering Strait for HS1. Using a high-resolution ocean model with an open Bering Strait, Praetorius et al.^33^ demonstrated that North Pacific freshwater could be transported to the North Atlantic via the Arctic route within a decade, leading to cooling in the North Atlantic. This Arctic pathway is likely more efficient than the Pacific–Indian–Atlantic route proposed for HS1 in this study and is equally, if not more effective, at weakening the AMOC. Given that the European Ice Sheets were significantly smaller during HS4, Pacific-sourced meltwater may have played a larger and more direct role during H4 than H1. Meanwhile, H3 represents a small and atypical Heinrich event, with Ruddiman Belt IRD dominated by European sources^10,47^. Our North Pacific hosing simulations suggest that even with a weak North Pacific freshwater forcing, the European ice sheets could still be impacted and lead to H3. However, these hypotheses remain to be tested with further studies, as our results show that the northern North Atlantic subsurface temperature response and the potential impacts on the Laurentide Ice Sheet are sensitive to the combined influences remotely from the North Pacific freshwater and locally from the European Ice Sheets. This highlights the need for future studies to evaluate Heinrich stadials on a case-by-case basis, accounting for event-specific forcings and boundary conditions. Expanded spatiotemporal coverage of proxy data, such as seawater chemical and physical properties, will be critical for further constraining such modeling efforts. Similarly, advances in next-generation eddy-resolving high-resolution models will improve the representation of narrow boundary currents, convections, and eddies, processes which play a key role in freshwater transport and AMOC strength^48,49^.

With respect to their individual impacts, our study demonstrates that freshwater forcings in the North Pacific and North Atlantic produce distinct global ocean and atmospheric responses. Previous studies have largely interpreted global and regional climate shifts during Heinrich stadials in paleoclimate records as driven by North Atlantic meltwater-forced Heinrich events, overlooking Siku events as a potential independent driver. However, multi-phased climate shifts during Heinrich stadials have been documented at various localities^50–52^. A well-known example is the dramatic “big dry” and “big wet” hydroclimate shifts observed over the southwestern US during HS1^50^. A two-phase shift, with drying during early HS1, synchronous with S1, and wetting during late HS1, synchronous with H1, is consistent with the drying shown in our North Pacific hosing simulations (Supplementary Fig. S14) and wetter conditions in previously reported North Atlantic hosing simulations^53^. Whether these phases represent distinct climate responses to consecutive Siku and Heinrich events needs to be investigated in detail with improved temporal resolution and a well-constrained chronology of proxy data in conjunction with model simulations in future studies. We recommend that future modeling efforts employ stepwise freshwater forcings in the North Pacific and the North Atlantic Oceans to more accurately simulate the complex climate dynamics of these intervals.

Lastly, our study shows that the AMOC is susceptible to both local and remote influences. Future changes in the thermohaline circulation may be driven not only by the melting of the Greenland and Antarctic ice sheets but also by salinity changes due to changes in evaporation, precipitation, and continental runoff both locally and remotely^54,55^. The advection of anomalous seawater salinity from remote regions to the North Atlantic is regulated at key ocean gateways, such as the Agulhas Leakage, the Drake Passage, and the Arctic Gateways, where seawater exchange is dynamically governed by air-sea interactions^38,56^. Improved representation of the global hydrological cycle and ocean circulation in climate models will be critical for reducing uncertainties in projections of future AMOC change.

## Methods

### Model setup

The climate model used in this study is the fully coupled isotope-enabled Community Earth System Model (CESM) version 1.3 (iCESM1.3)^34,35^. The model components include the Community Atmospheric Model version 5 (CAM5) for the atmosphere, the Community Land Model version 4 (CLM4) for land, the Parallel Ocean Program version 2 (POP2) for the ocean, and the Community Ice Code version 4 (CICE4) for sea ice. The atmosphere and the land models have a horizontal resolution of 1.9° × 2.5°, and the ocean and the sea ice models have a nominal horizontal resolution of 1°. The atmosphere model has 30 vertical levels, and the ocean model has 60 vertical levels. Previous multi-model comparisons of North Atlantic hosing simulations indicate that this model ranks among the top when evaluated against a global synthesis of hydroclimate responses to Heinrich events^3,57^.

The control case used in this study is the 19 ka climate from the recently released isotope-enabled transient climate experiment (iTRACE) of the last deglacial^34^. iTRACE was run on the same iCESM1.3 model setup as described in this study. It incorporates time-evolving boundary conditions and external forcings between 20-11 ka, including orbital change, greenhouse gas concentrations, and topography, sea level, and ice sheets from the ICE-6GC reconstruction^58^, providing a realistic transient climate simulation of the last deglacial^34^. The average of 19-19.05 ka climate is used as the control to compare against our hosing simulations. The relatively stable AMOC and North Atlantic conditions over this 50-year period suggest that decadal variability is small compared to the forced changes examined here (Fig. 2). Using only 50 years minimizes the potential impacts of other varying deglacial forcings (i.e., orbit and greenhouse gases).

Since iTRACE was originally run on an older supercomputer, Yellowstone at the National Science Foundation National Center for Atmospheric Research (NSF NCAR), we run a 200-year extension of the 19 ka control simulation (ctrl_ext) on the new NSF NCAR supercomputer, Derecho, to test the reproducibility of the model results across different machines. This simulation shows a stable climate over the 200-year duration and no long-term drift in AMOC strength (Supplementary Fig. S2), suggesting that the global ocean and atmosphere are in equilibrium in the control case.

### North Pacific hosing simulations

The Northeast Pacific hosing simulations reported in this study are branched from the 19 ka climatology of iTRACE to best represent the boundary conditions at the onset of Siku 1. To capture the possible range of freshwater fluxes and test model sensitivity to Siku meltwater forcing, we run a set of Northeast Pacific hosing simulations with three forcing intensities: 0.1, 0.2, and 0.5 Sv. Freshwater is continuously added to the Northeast Pacific (45–70°N, 160–235°E; Supplementary Fig. S1) for 400 years in the NP01, NP02, and NP05 simulations, respectively. A passive dye tracer is added continuously to the hosing region to track the transport and mixing of the Northeast Pacific water mass across the global ocean. The average of the last 50 years of each simulation is calculated to compare against the 19 ka control simulation.

The total added freshwater in our simulations is 3.8, 7.5, and 18.8 m s.l.e. in NP01, NP02, and NP05, respectively, which falls within the estimated magnitude of rapid sea-level rise around 19 ka (10–15 m)^59^. Meanwhile, we acknowledge that the imposed meltwater forcing in NP02 and especially NP05 may have overestimated the actual meltwater contribution from the Cordilleran Ice Sheet during S1, given that the total Cordilleran ice sheet volume is estimated to be ~ 8.8 m s.l.e^60^. and that melt from other ice sheets also contributed to the global sea-level change at 19 ka^59,61^. However, such exaggerations are not unreasonable because the current generation of general circulation models is known to produce an overly stable AMOC^62^. For example, while H1 meltwater release from the Laurentide Ice Sheet has been estimated at only ~ 0.02 Sv^63^, substantially stronger forcings (0.25 to 1 Sv) are typically required for the models to achieve atmospheric and oceanic responses that are consistent with proxy data, including the model used in this study^3,57,64^. Indeed, previous studies found that when a smaller freshwater forcing, such as 0.1 Sv, applied to the North Atlantic in CESM versions 1.2 or 1.3, the AMOC response in the model is too small, failing to propagate the climate signal outside of the Atlantic Ocean basin^3,57^ or drive subsurface warming in the North Atlantic^20^. The models’ over-stability may result from model biases in surface water salinity in the South Atlantic, which cause the models to have a net import of freshwater in the South Atlantic, contradicting the net export shown in observational data^65^. This bias salinifies the Atlantic Ocean when the AMOC is weakened, internally reinvigorating the AMOC when the external forcing is too small^65^. In this context, our slight exaggeration of freshwater forcing is not only justified but also allows us to test the sensitivity and robustness of the AMOC’s response to varying forcing intensities. The qualitatively similar responses across NP01, NP02, and NP05 (Fig. 2) suggest that the same Pacific-Indian-Atlantic freshwater transport mechanism drives AMOC weakening regardless of forcing intensity.

### BIIS hosing simulations

We run two sets of simulations to assess the impact of a BIIS meltwater event. In the first set, we apply a freshwater flux of 0.05 or 0.1 Sv to the Irish west coast (0–15°W, 50–60°N; Supplementary Fig. S1) for 200 years (i.e., NP02_BIIS005 and NP02_BIIS01; Supplementary Table S1), branching from year 400 of NP02, with the Atlantic already freshened and the AMOC weakened. The moderate North Pacific hosing scenario, NP02, is chosen to provide boundary conditions because its North Atlantic responses agree well with proxy data of surface and subsurface changes during S1 (Fig. 4). The total added BIIS freshwater is 0.94 and 1.88 m s.l.e. in NP02_BIIS005 and NP02_BIIS01, respectively, within the estimated freshwater contribution based on ice sheet modeling^42^ and thus represent a conservative forcing, considering the model’s over-stability bias discussed above. In both NP02_BIIS005 and NP02_BIIS01 simulations, the AMOC weakens rapidly within the first 50 years (Supplementary Fig. S12). However, in contrast to the continued weakening in NP02_BIIS01, the AMOC in NP02_BIIS005 begins to gradually strengthen after year 50 (Supplementary Fig. S12). This is because the water masses entering the Atlantic basin through the Agulhas Leakage become saltier as the remaining North Pacific freshwater becomes exhausted, which outcompetes the weak freshwater forcing applied in the Northeast Atlantic in this experiment (i.e., NP02_BIIS005). The 50-year duration is also consistent with the timing that we noted for water mass transport from the North Pacific to the North Atlantic NADW formation sites (Fig. 2m–o). This comparison suggests that the 0.05 Sv forcing scenario is likely too weak, while NP02_BIIS01 is the more realistic scenario for Heinrich Stadial 1.

In the second set of BIIS simulations, we apply the same 0.05 or 0.1 Sv of freshwater fluxes to the Irish west coast, but under the boundary conditions of the 19 ka control (i.e., “ctrl_BIIS005” and “ctrl_BIIS01”; Supplementary Table S1), to evaluate the preconditioning effects of Siku meltwater. The comparison between this set of simulations with their NP02 counterparts (i.e., NP02_BIIS01 vs. ctrl_BIIS01 and NP02_BIIS005 vs. ctrl_BIIS005) shows that the AMOC is consistently weaker when the North Atlantic has already been freshened after a Siku event (Supplementary Fig. S12), suggesting an important role of pre-existing Siku meltwater in enhancing AMOC weakening.

### Positive degree day model

We use a positive degree day (PDD) ice sheet surface mass balance model to estimate the atmospheric impacts of North Pacific meltwater on Laurentide and European ice sheets. This model assumes that melt is proportional to the sum of daily mean air temperature above 0 ˚ C (i.e., PDD). Because our model output data is at a monthly resolution, a normal distribution is assumed for daily temperature variations. For each month, the monthly mean PDD_*m*_ (°C day) is calculated using the equation^66^:

1 $${{{\rm{PDD}}}}_{m}={N}_{m}\cdot \frac{1}{\sigma \sqrt{2\pi }}{\int }_{\!\!\!\!0}^{\infty }T\cdot \exp \left(-\frac{{\left(T-{T}_{m}\right)}^{2}}{2{\sigma }^{2}}\right){dT}$$

where *N*_*m*_ is the number of days in month m; *T*_*m*_ is the monthly mean surface temperature from our model output. *σ* is assumed to be 5 ˚C^67^. The equation can be solved using an error function following Calov and Greve^68^:

2 $${{{\rm{PDD}}}}_{m}={N}_{m}\cdot \left[\frac{\sigma }{\sqrt{2\pi }}\exp \left(-\frac{{{T}_{m}}^{2}}{2{\sigma }^{2}}\right)+\frac{{T}_{m}}{2}{{\rm{erfc}}}\left(-\frac{{T}_{m}}{\sqrt{2}\sigma }\right)\right]$$

Monthly surface mass balance, SMB_*m*_, is calculated as

3 $${{{\rm{SMB}}}}_{m}={S}_{m}-{M}_{m}+{R}_{m}$$

where *S*_*m*_ is the monthly mean snowfall accumulation (mm/month) from our model output; *M*_*m*_ is monthly snow melt rate (mm/month), and *R*_*m*_ is the refreeze of melted snow (mm/month). *M*_*m*_ and *R*_*m*_ are calculated as:

4 $${M}_{m}={D}_{{{\rm{snow}}}}\cdot {{{\rm{PDD}}}}_{m}$$

5 $${R}_{m}={f}_{{{\rm{refreeze}}}}\cdot {M}_{m}$$

where *D*_snow_ is a constant degree-day factor of 3 mm ˚C^-1^ day^-1 66,67^, and *f*_refreeze_ is the refreezing factor, which is set as 0.5 in this study, commonly used for the Greenland Ice Sheet today^66^. The annual mean ice sheet mass balance is calculated as the sum of SMB_*m*_.

## Supplementary information

Supplementary Information

Transparent Peer Review file

## Data Availability

The model data generated in this study are available at Figshare^69^ (10.6084/m9.figshare.32653614).

## Code availability

The MATLAB code used to perform the δ^18^O_sw-ivc_ data averaging and ice sheet surface mass balance is available at Figshare^69^ (10.6084/m9.figshare.32653614).
