# Supplementary material for: North Pacific meltwater weakens the Atlantic Meridional Overturning Circulation and preconditions Heinrich Stadial 1
Source: Nat Commun. 2026 Jul 4;17:8389. doi: 10.1038/s41467-026-75199-y (PMC13473526; doi:10.1038/s41467-026-75199-y)
Supplement: Supplementary file 1 [file 41467_2026_75199_MOESM1_ESM.pdf]

Supplementary Information for

North Pacific meltwater weakens the Atlantic Meridional Overturning Circulation and preconditions Heinrich Stadial 1

Chijun Sun[1,2 *], Jiang Zhu[2], Bette L. Otto-Bliesner[2], Esther C. Brady[2], Sophia I. Macarewich[2]

[1] Department of Earth and Planetary Sciences, University of California Davis, Davis, CA 95616
[2] Climate & Global Dynamics, NSF National Center for Atmospheric Research, Boulder, CO 80301

*Corresponding author: Chijun Sun (cjsun@ucdavis.edu)

SUPPLEMENTARY TEXT

1. Inter-basin freshwater transport

The dye tracer concentration across the global oceans, which tracks the transport and mixing of the added freshwater in the North Pacific, shows that the freshwater is quickly mixed into the North Pacific subtropical gyre and transported to the lower latitudes (Fig. S4). At approximately model year 10 in both NP02 and NP05, the freshwater is transported across Indonesia and into the Indian Ocean, where it continues to propagate westward along the Indian Ocean Equatorial Currents. Around model year 15, the freshwater begins to leak into the South Atlantic Ocean through the Agulhas Leakage. In the Atlantic basin, the upper branch of an active AMOC transports this freshwater northward. The northern North Atlantic deep water formation region begins to receive this North Pacific-originated freshwater at approximately year 40. The transport route and time are broadly consistent with modern observations, where water mass can be transported from the Indonesian Throughflow to the tropical South Atlantic in about 30 years [1], suggesting that the simulated freshwater transport is realistic.

In our simulations, the freshwater is mostly transported in the upper ocean, with more than 90% of the added dye tracer remaining in the upper 1000 m of the global ocean by year 200 in both NP02 and NP05. This suggests that the vast majority of the hosed freshwater is transported via the surface transport pathway described above. Unlike the North Atlantic, there is a lack of deep water formation in the North Pacific, which prevents extensive downward mixing of water in this region. The freshwater forcing further lowers the density of North Pacific surface water, which strengthens the stratification. Indeed, proxy data show increased radiocarbon age of intermediate waters in the North Pacific during Siku events, which is likely driven by reduced mixing and increased stratification [2].

2. Dynamics of Northeast Atlantic subsurface warming

The North Atlantic subsurface temperature change is characterized by cooling at the subpolar North Atlantic and warming to the south (Fig. 4). This warming off the west coast of Ireland is part of a broad band of warming along the eastern sector of the North Atlantic Current which separates the North Atlantic subpolar and subtropical gyres (“SPG” and “STG” hereafter). It is also present at the surface, albeit slightly away from the shoreline (Fig. S15). A stronger correlation between the Northeast Atlantic subsurface warming and the SPG index (defined as mean barotropic stream function between 10-60°W and 50-62°N [3-5]) than between the subsurface warming and the AMOC strength suggests that the warming is more directly associated with a weaker SPG (Fig. S8-9). Changes in the barotropic stream function in the North Atlantic suggest that there is a weakening of the SPG, especially along its southern edge (Fig. S8). Consequently, the northward transport of warm tropical water mass into the northern North Atlantic (NNA) via the SPG is reduced, resulting in cooling of the NNA and accumulation of heat along the North Atlantic Current. In addition, there is a strengthening and northward shift of the STG along its northern edge, which would incorporate more warm tropical water into the North Atlantic Current, leading to further warming.

The strength of the SPG is known to be controlled by the radial density gradient of the SPG and wind stress over the North Atlantic [6-8]. In our simulations, both oceanic and atmospheric dynamics contribute to the weakening of the SPG. The arrival of the North Pacific freshwater to

the NNA decreases the upper ocean density at both the central and eastern SPG (Fig. S1). An increased sea ice meltwater flux causes additional freshening in the central SPG (Fig. S5) when a weakened AMOC drives an expansion of sea ice in the northern North Atlantic (Fig. S5), which relaxes the radial density gradient and consequently weakens the SPG strength (Fig. S9). Additionally, cooling over the NNA results in a positive sea-level pressure anomaly in NP02 and NP05, which drives anticyclonic wind anomalies over this region, producing wind stress that further weakens the SPG (Fig. S15).

SUPPLEMENTARY FIGURES

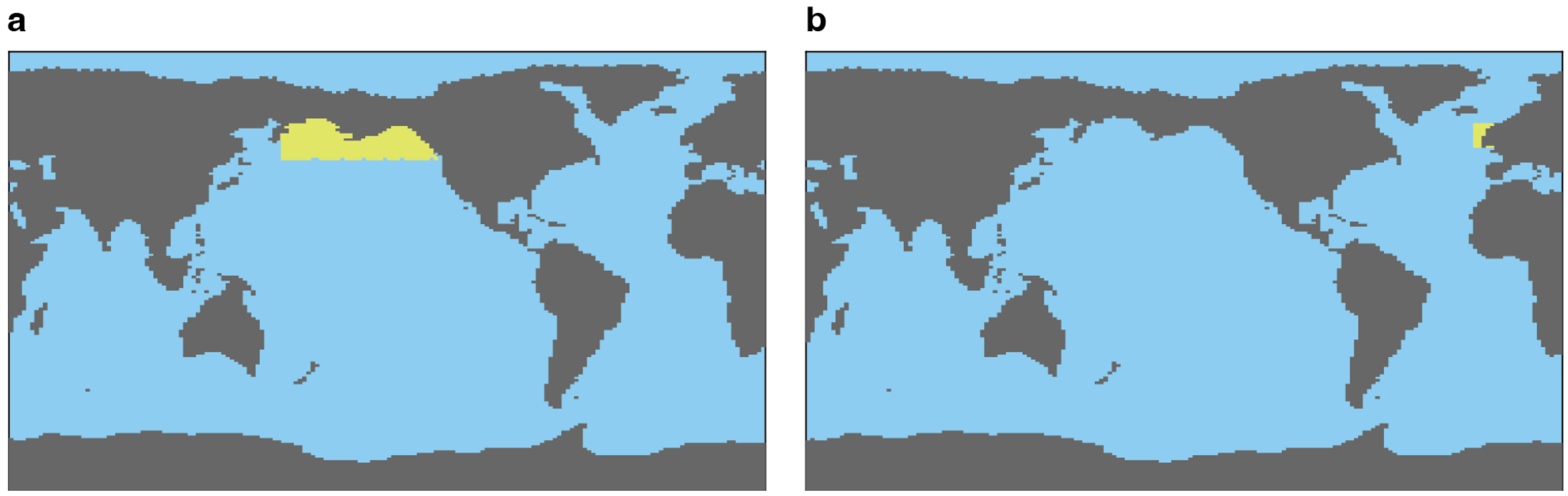

Figure S1 | Maps showing where freshwater forcing is applied in hosing simulations. (a) North Pacific hosing experiments (i.e., NP01, NP02, and NP05). **(b)** British-Irish Ice Sheet (BIIS) hosing experiments (i.e., NP02_BIIS005, NP02_BIIS01, ctrl_BIIS005, and ctrl_BIIS01). Yellow shading highlights the hosing region. Maps were generated using the m_map mapping package for MATLAB [9] and the Climate Data Toolbox for MATLAB [10].

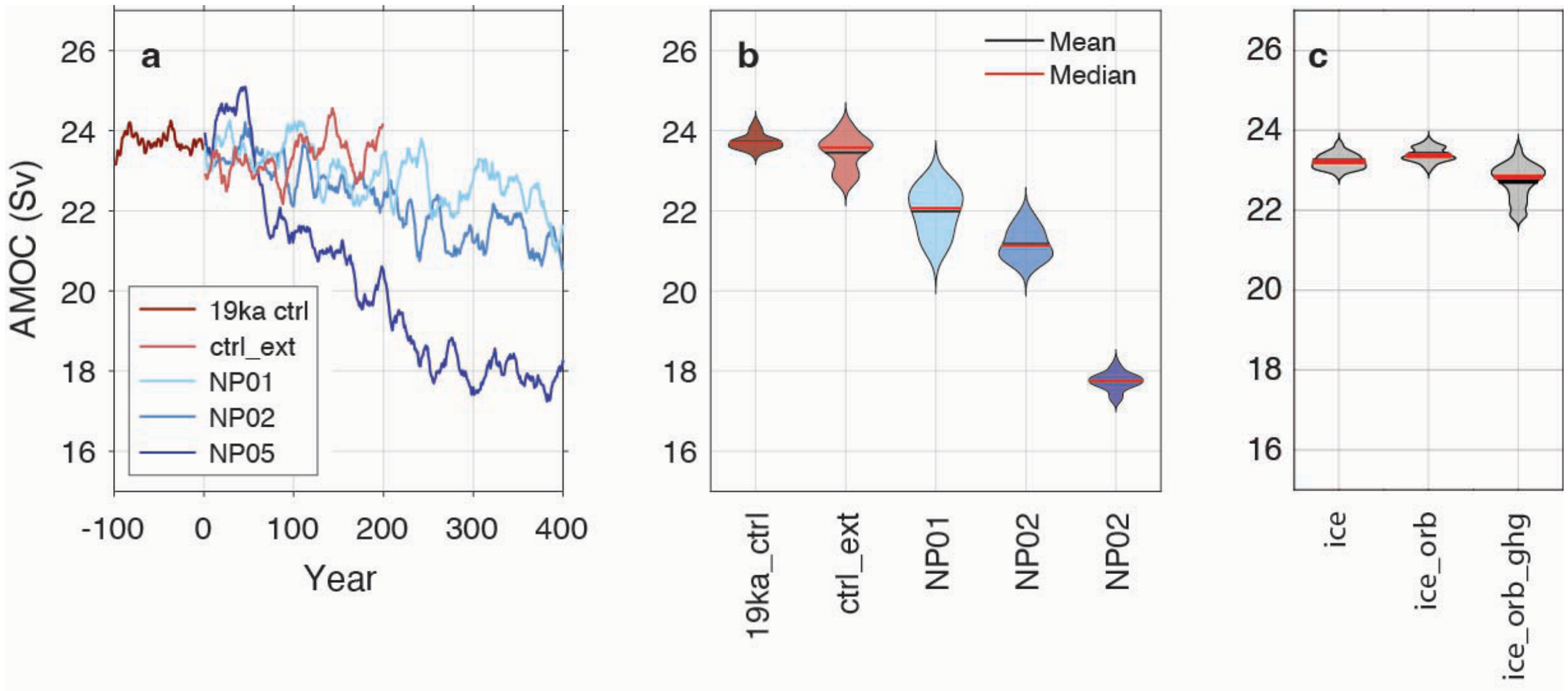

Figure S2 | Simulated AMOC responses to North Pacific hosing compared to the control run and iTRACE factorized forcing runs. (a) Evolution of AMOC strength in the 19 ka control, extended control, NP01, NP02, and NP05 simulations. **(b)** Violin plots showing the distribution of AMOC strength during the last 50 years of each simulation. **(c)** Violin plots showing the distribution of AMOC strength during 19–18 ka in iTRACE factorized-forcing runs forced with ice sheet (ice), ice sheet + orbital configurations (ice_orb), and ice sheet + orbital configurations + greenhouse gas (ice_orb_ghg). AMOC values are smoothed using a 10-year moving average. The AMOC strength is significantly weaker in all three North Pacific hosing experiments compared to the 19 ka control, the extended control simulations, and the iTRACE factorized-forcing runs according to a Student's *t*-test.

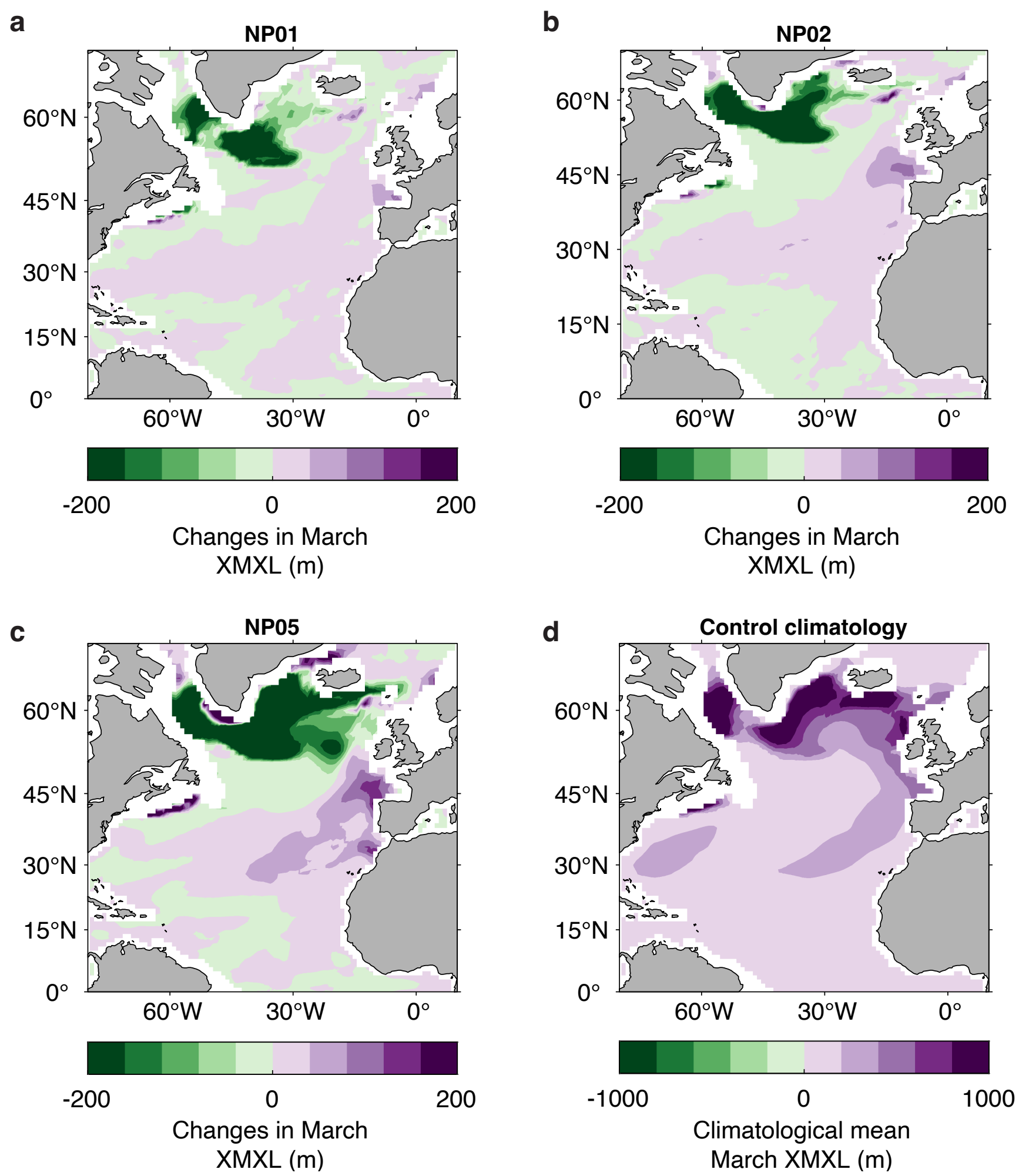

Figure S3 | Changes in mean March maximum mixed-layer depth (XMXL). (a) Difference in mean March XMXL between NP01 and the 19 ka control. **(b)** Difference in mean March XMXL between NP02 and the 19 ka control. **(c)** Difference in mean March XMXL between NP05 and the 19 ka control. **(d)** Climatological mean March XMXL in the 19 ka control simulation. Maps were generated using the m_map mapping package for MATLAB [9] and the Climate Data Toolbox for MATLAB [10].

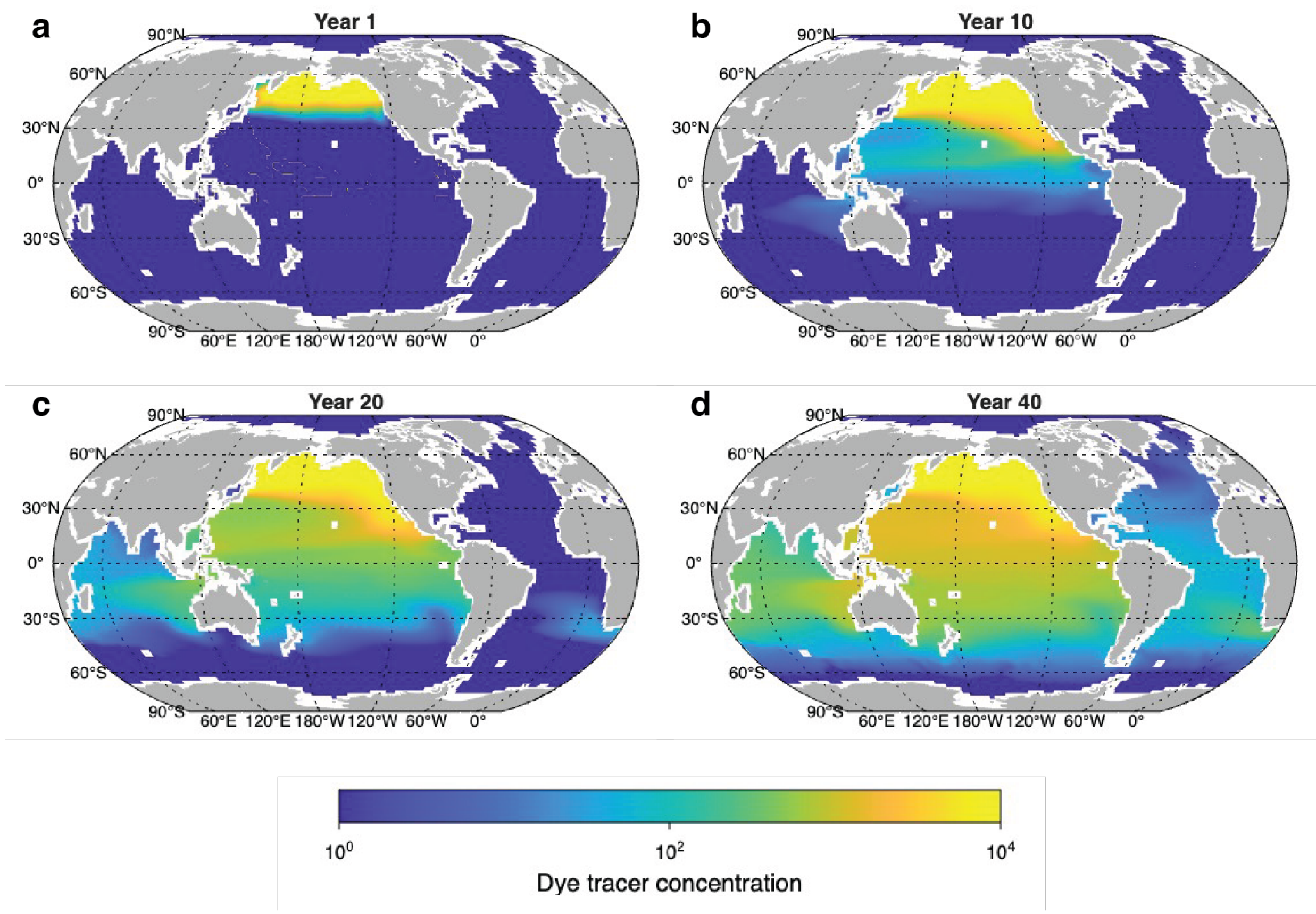

Figure S4 | Evolution of passive dye tracer concentration in the global ocean in the NP02 experiment. Panels show dye tracer concentrations at **(a)** Year 1, **(b)** Year 10, **(c)** Year 20, and **(d)** Year 40 after the onset of freshwater forcing, illustrating a Pacific-Indian-Atlantic freshwater transport pathway. Maps were generated using the m_map mapping package for MATLAB [9] and the Climate Data Toolbox for MATLAB [10].

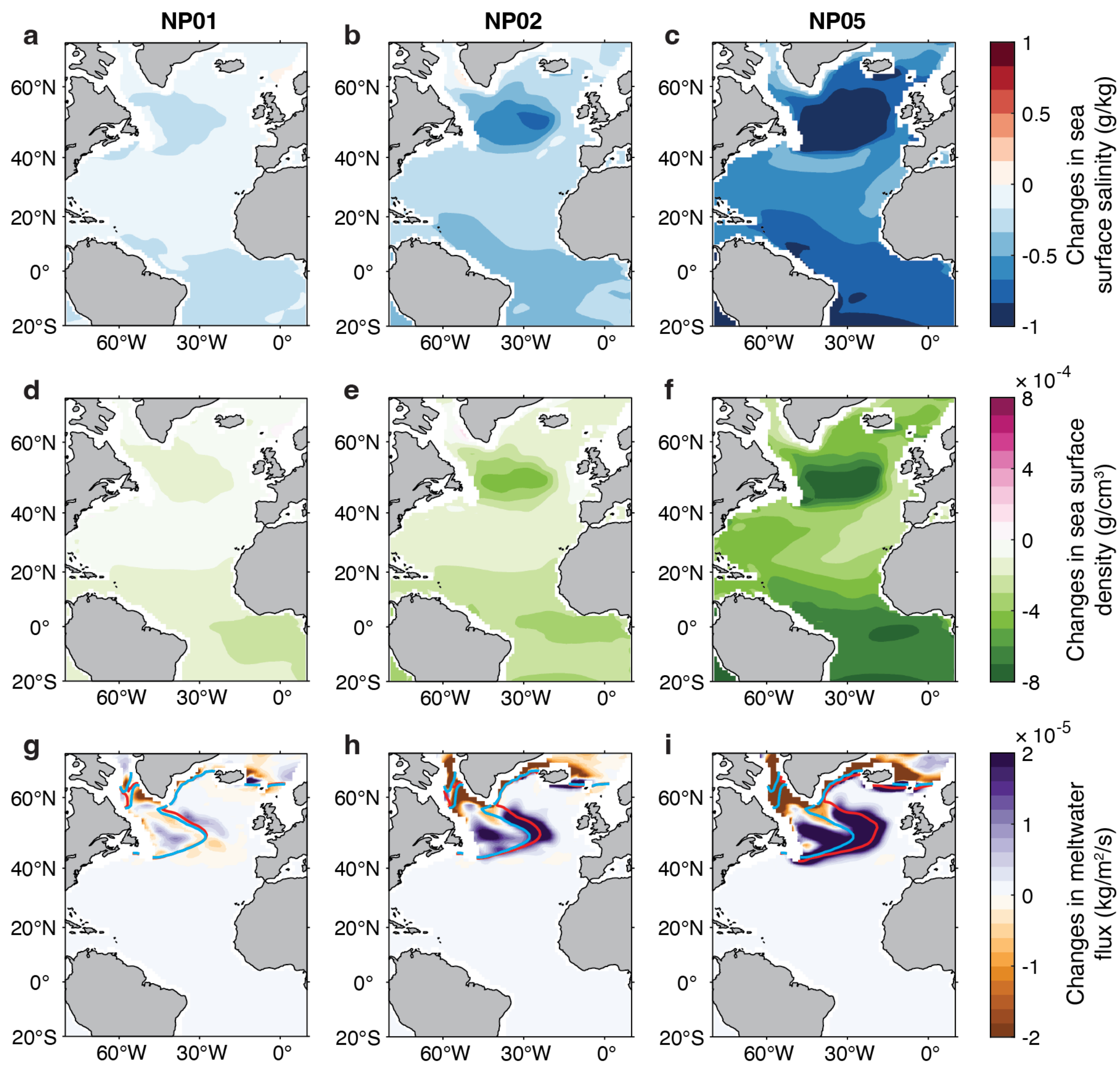

Figure S5 | Maps of mean annual changes in the North Atlantic relative to the 19 ka control. **(a)** Sea surface salinity change in NP01; **(b)** sea surface salinity change in NP02; **(c)** sea surface salinity change in NP05. **(d)** Sea surface potential density change in NP01; **(e)** sea surface potential density change in NP02; **(f)** sea surface potential density change in NP05. **(g)** Sea-ice meltwater flux change in NP01; **(h)** sea-ice meltwater flux change in NP02; **(i)** sea-ice meltwater flux change in NP05. In **(g–i)**, the blue and red lines indicate the February sea-ice margins (defined as the 15% sea-ice concentration contour) in the 19 ka control and hosing simulations, respectively. Maps were generated using the m_map mapping package for MATLAB [9] and the Climate Data Toolbox for MATLAB [10].

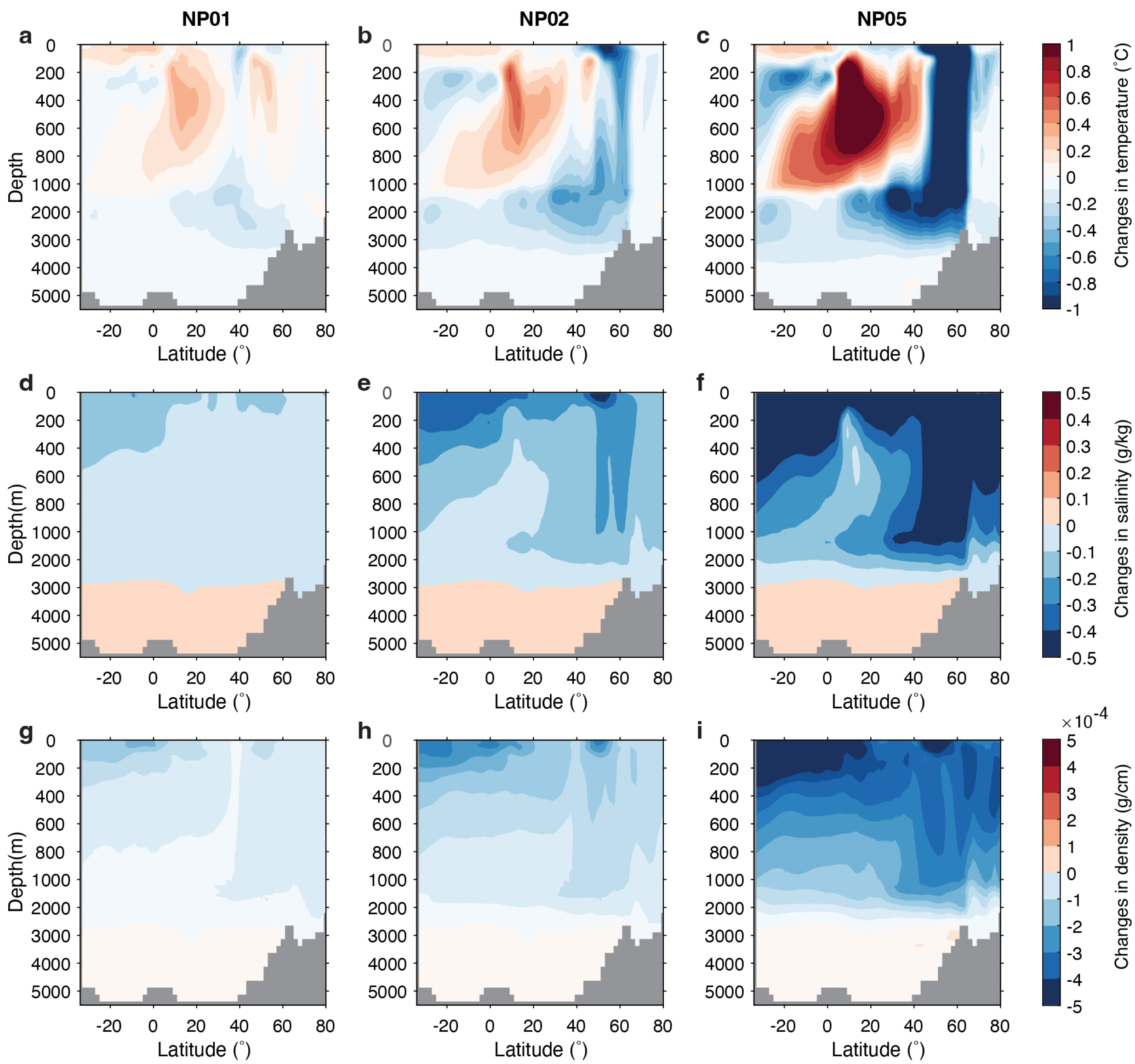

Figure S6 | Zonally averaged Atlantic Ocean anomalies relative to the 19 ka control simulation. Temperature anomalies in **(a)** NP01, **(b)** NP02, and **(c)** NP05; salinity anomalies in **(d)** NP01, **(e)** NP02, and **(f)** NP05; and potential density anomalies in **(g)** NP01, **(h)** NP02, and **(i)** NP05.

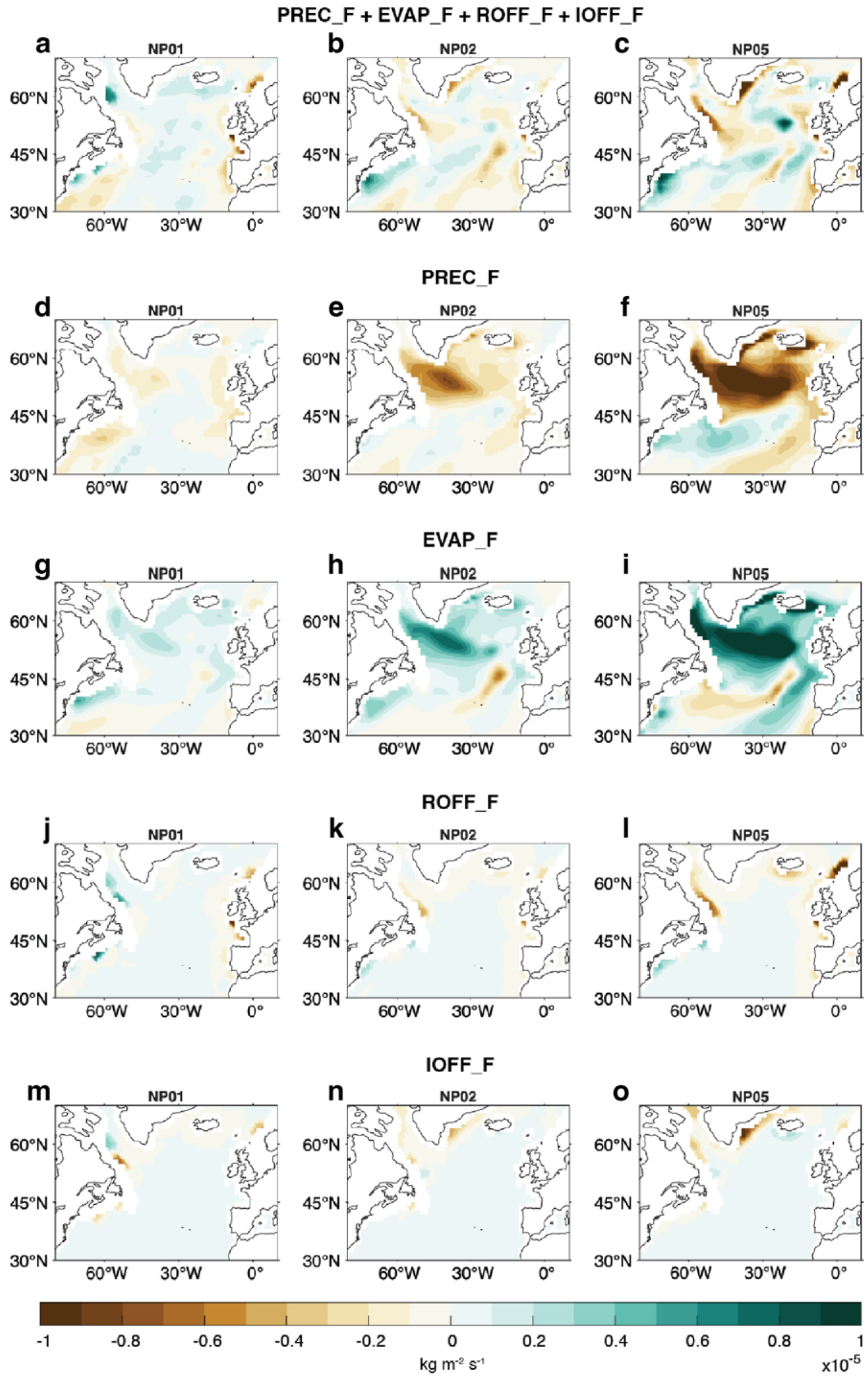

Figure S7 | Changes in freshwater flux components over the North Atlantic relative to the 19 ka control simulation. Net freshwater flux anomaly (PREC_F + EVAP_F + ROFF_F + IOFF_F) in **(a)** NP01, **(b)** NP02, and **(c)** NP05; precipitation anomaly (PREC_F) in **(d)** NP01, **(e)** NP02, and **(f)** NP05; evaporation anomaly (EVAP_F) in **(g)** NP01, **(h)** NP02, and **(i)** NP05; runoff anomaly (ROFF_F) in **(j)** NP01, **(k)** NP02, and **(l)** NP05; and ice runoff anomaly from land snow capping (IOFF_F) in **(m)** NP01, **(n)** NP02, and **(o)** NP05. Maps were generated using the m_map mapping package for MATLAB [9] and the Climate Data Toolbox for MATLAB [10].

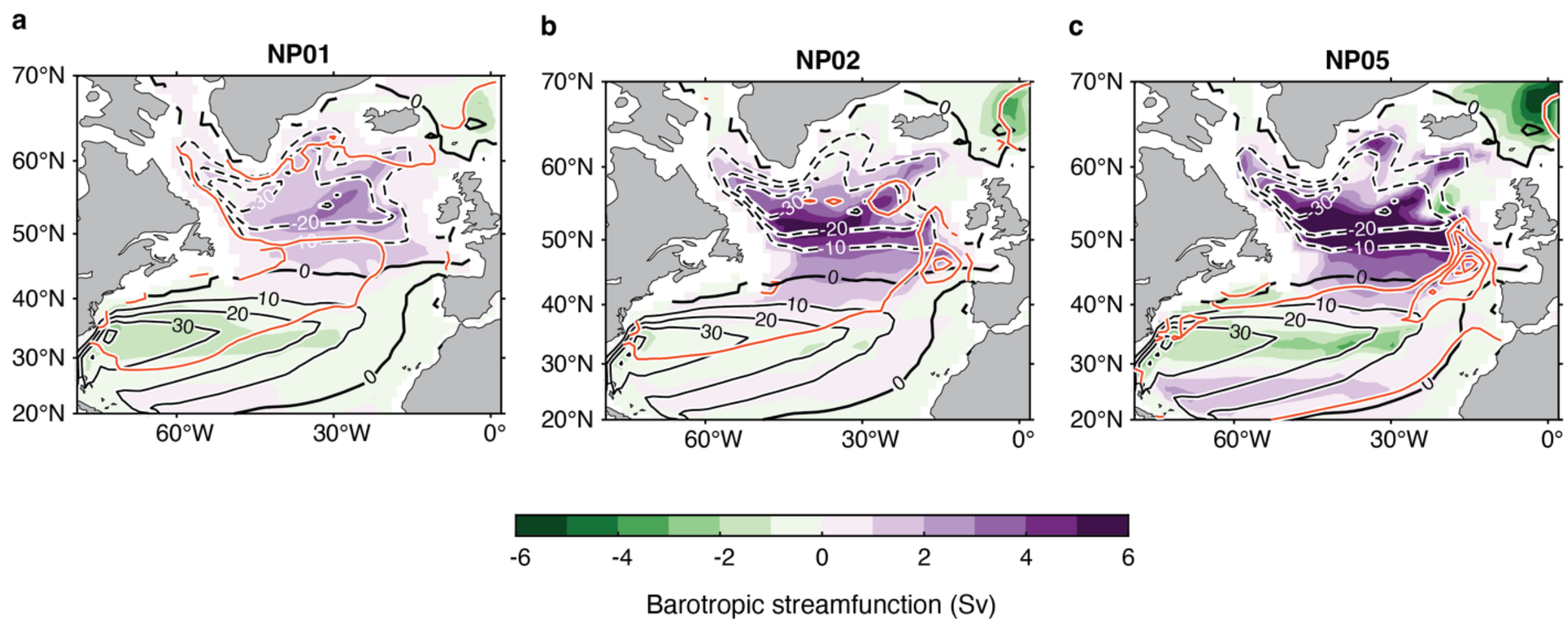

Figure S8 | Northeastern Atlantic subsurface warming associated with a weakened North Atlantic subpolar gyre. Annual mean anomalies of barotropic stream function (BSF; color shading) and 500-meter depth temperature (orange contours; the contour interval is 1°C; only non-negative contours are shown) in **(a)** NP01, **(b)** NP02, and **(c)** NP05 relative to the 19 ka control simulation. Black contours denote the climatological mean BSF, with solid and dashed contours indicating positive and negative values, respectively, and marking the locations of the climatological subtropical (positive BSF) and subpolar (negative BSF) gyres. The thick black contour denotes the zero BSF contour. Positive BSF anomalies within the subpolar gyre indicate a weakening of the subpolar gyre. Maps were generated using the m_map mapping package for MATLAB [9] and the Climate Data Toolbox for MATLAB [10].

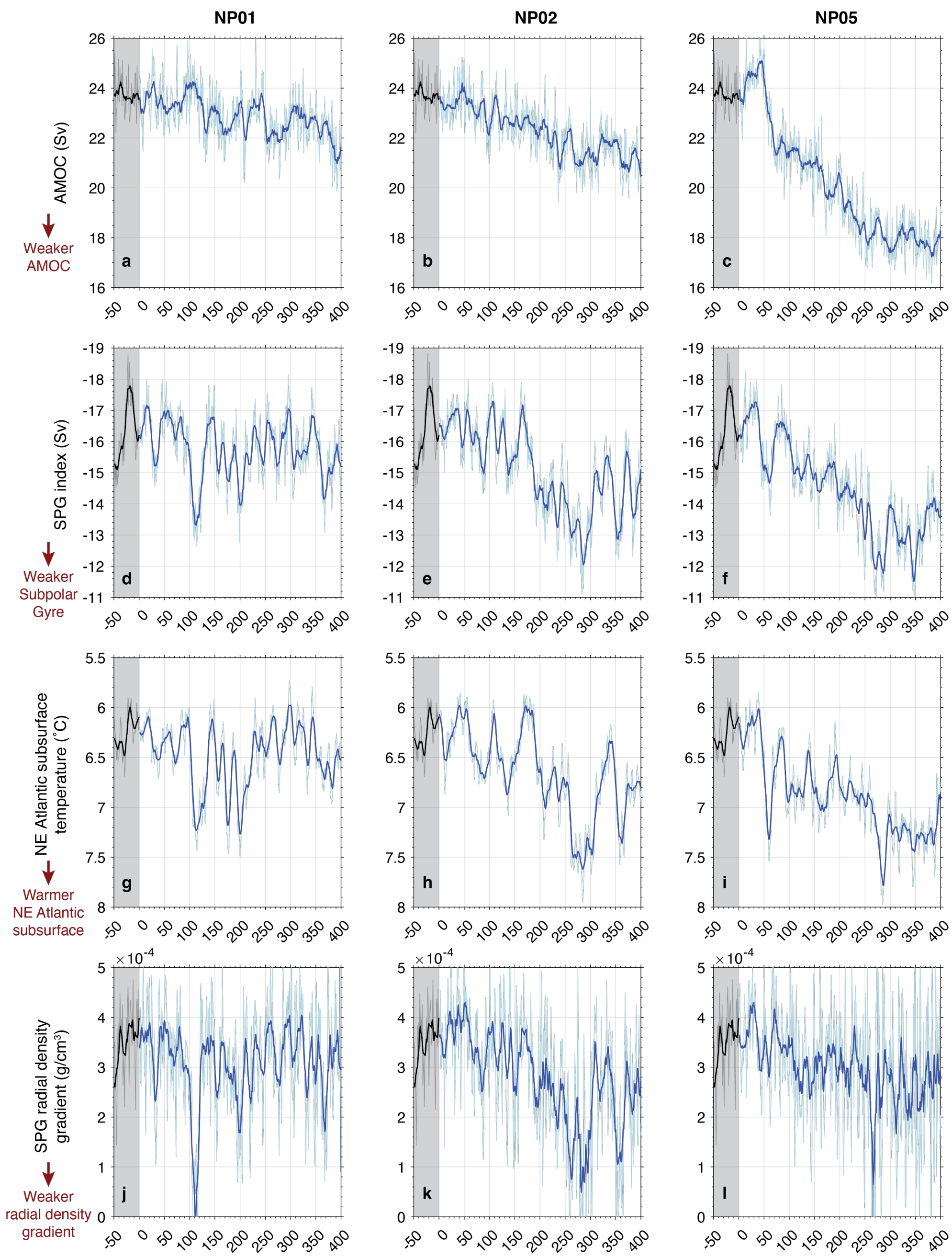

Figure S9 | North Atlantic subtropical gyre weakening driven by North Pacific freshwater forcing. Evolution of AMOC strength in **(a)** NP01, **(b)** NP02, and **(c)** NP05; subpolar gyre (SPG) index, defined as the mean barotropic streamfunction between 10–60°W and 50–62°N, in **(d)** NP01, **(e)** NP02, and **(f)** NP05; northeastern Atlantic subsurface temperature (40-50°N, 10-20°W, 300-700 meter depth) in **(g)** NP01, **(h)** NP02, and **(i)** NP05; and radial density gradient in the SPG, defined as the difference between surface density in the eastern SPG (15-20°W, 50-60°N) and central SPG (40-45°W, 50-60°N) regions, in **(j)** NP01, **(k)** NP02, and **(l)** NP05. The light-colored curves represent annual mean values, and the dark-colored curves represent 10-year moving average values in control (grey/black) and hosing simulations (blues). The gray bars denote the control simulation.

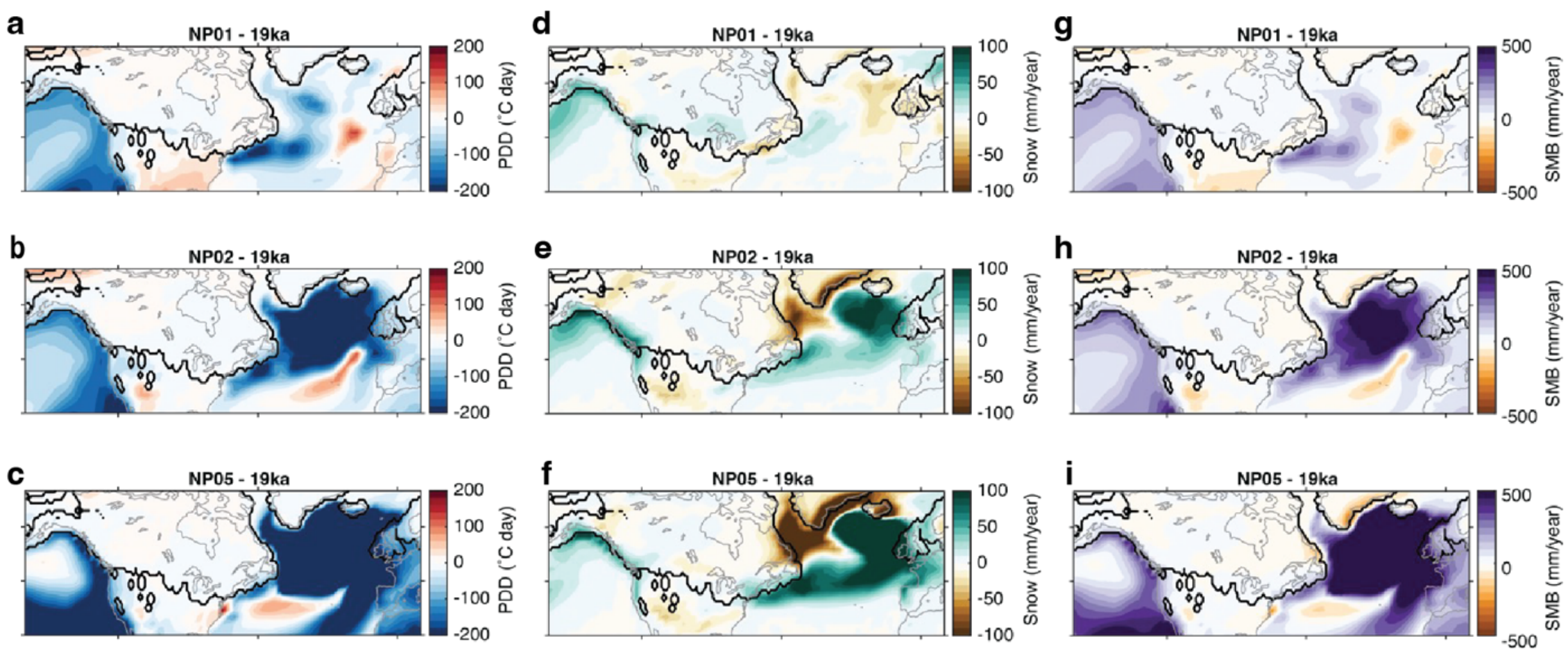

Figure S10 | Estimated impacts of North Pacific meltwater on ice-sheet surface mass balance. Changes in mean annual positive degree days relative to the 19 ka control in **(a)** NP01, **(b)** NP02, and **(c)** NP05; changes in mean annual snowfall in **(d)** NP01, **(e)** NP02, and **(f)** NP05; and changes in mean annual surface mass balance in **(g)** NP01, **(h)** NP02, and **(i)** NP05. Thick black contours delineate the extent of the ice sheets. Maps were generated using the m_map mapping package for MATLAB [9] and the Climate Data Toolbox for MATLAB [10].

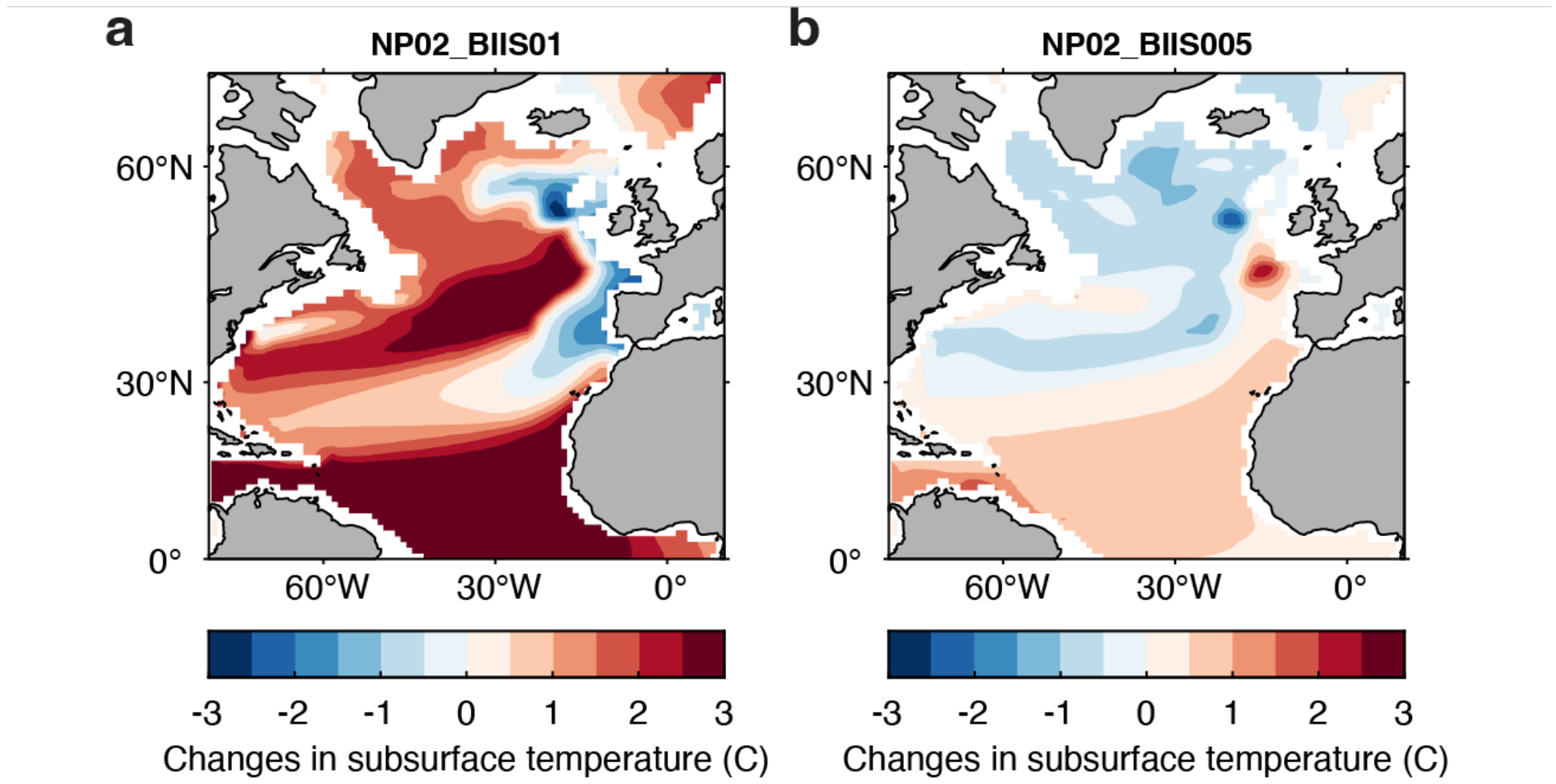

Figure S11 | North Atlantic subsurface temperature response to meltwater from the British-Irish Ice Sheet (BIIS). (a) NP02_BIIS01, **(b)** NP02_BIIS005. Both simulations are branched from year 400 of NP02. Maps were generated using the m_map mapping package for MATLAB [9] and the Climate Data Toolbox for MATLAB [10].

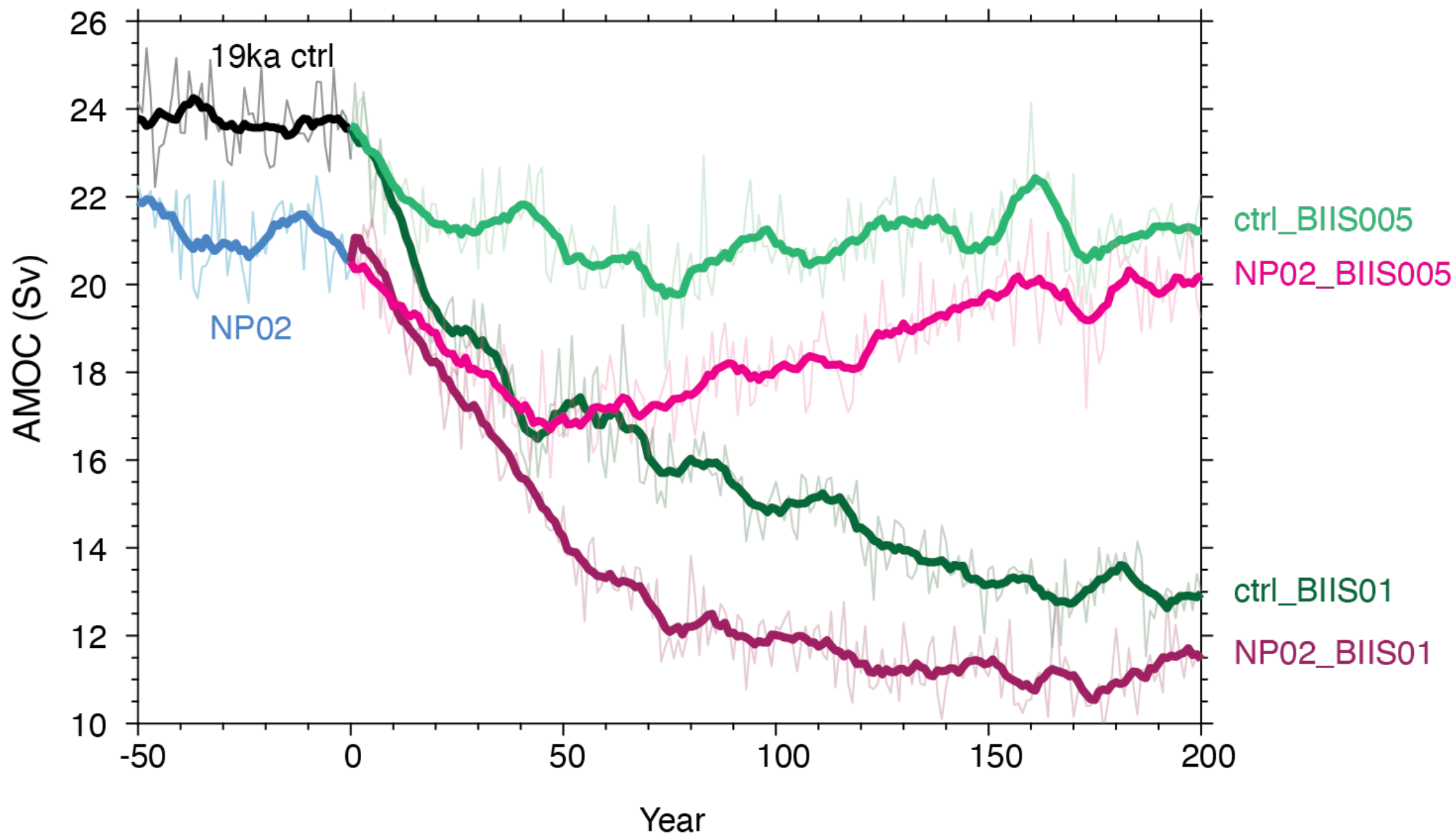

Figure S12 | The impact of Siku preconditioning on AMOC response to BIIS meltwater. Comparison of AMOC changes in BIIS experiments branched from the 19ka control (ctrl_BIIS01 and ctrl_BIIS005; red curves) vs. those from the NP02 (NP02_BIIS01 and NP02_BIIS005; green curves) boundary conditions. The simulations branched from NP02 consistently show a weaker AMOC compared to their counterparts branched from the 19ka control run.

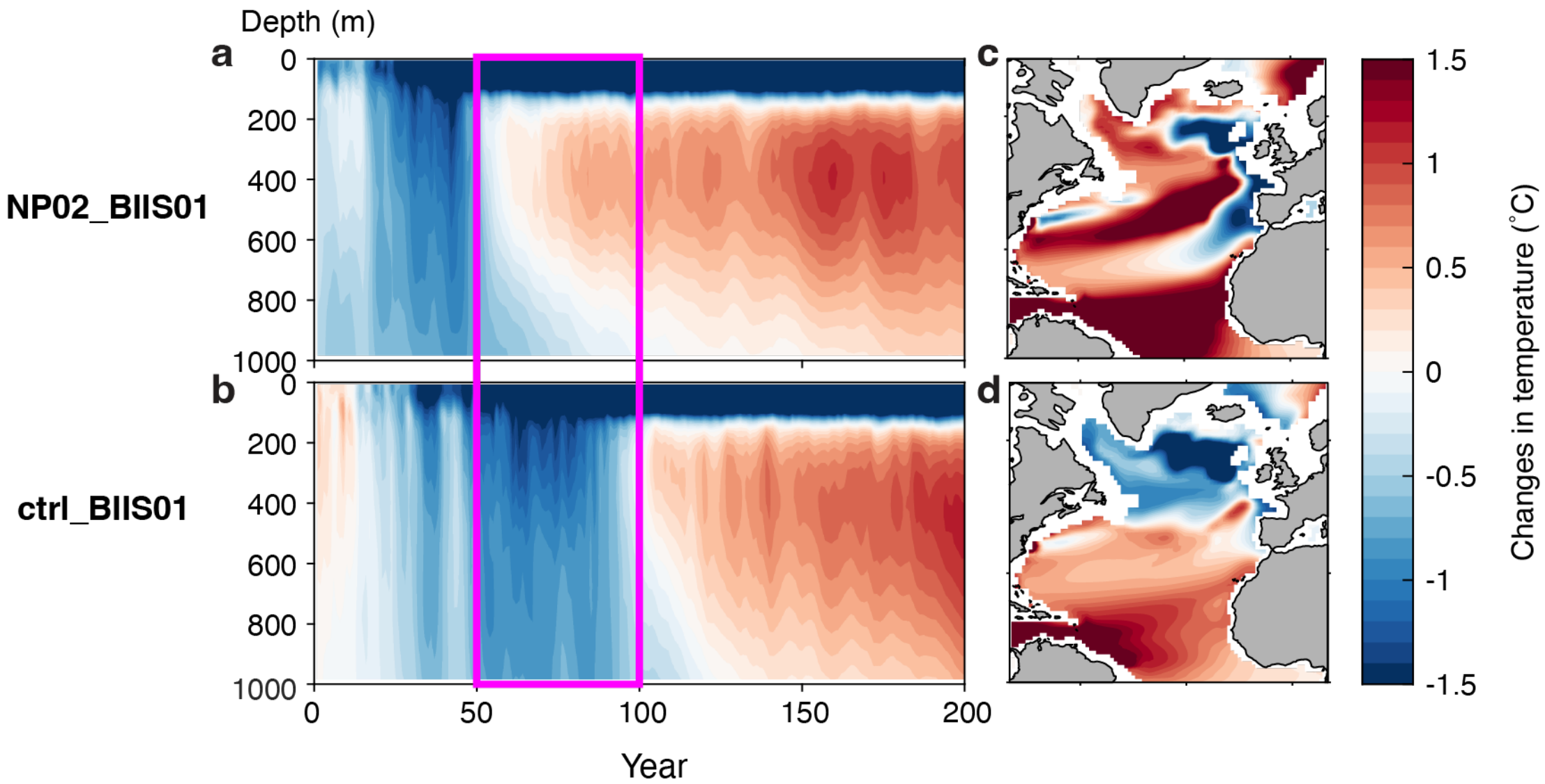

Figure S13 | The impact of Siku preconditioning on North Atlantic subsurface temperature response to BIIS meltwater. (a-b) Hovmoller plots of northern North Atlantic (north of 45°N, including the Labrador Sea but not the Greenland-Iceland-Norwegian Seas) temperature change in depth and time in **(a)** NP02_BIIS01 and **(b)** ctrl_BIIS01, respectively. **(c-d)** North Atlantic subsurface temperature change in **(c)** NP02_BIIS01 and **(d)** ctrl_BIIS01 averaged over year 50-100 of the simulations. The magenta box on the left panels highlights the difference between the two simulations between year 50 and 100. Maps were generated using the m_map mapping package for MATLAB [9] and the Climate Data Toolbox for MATLAB [10].

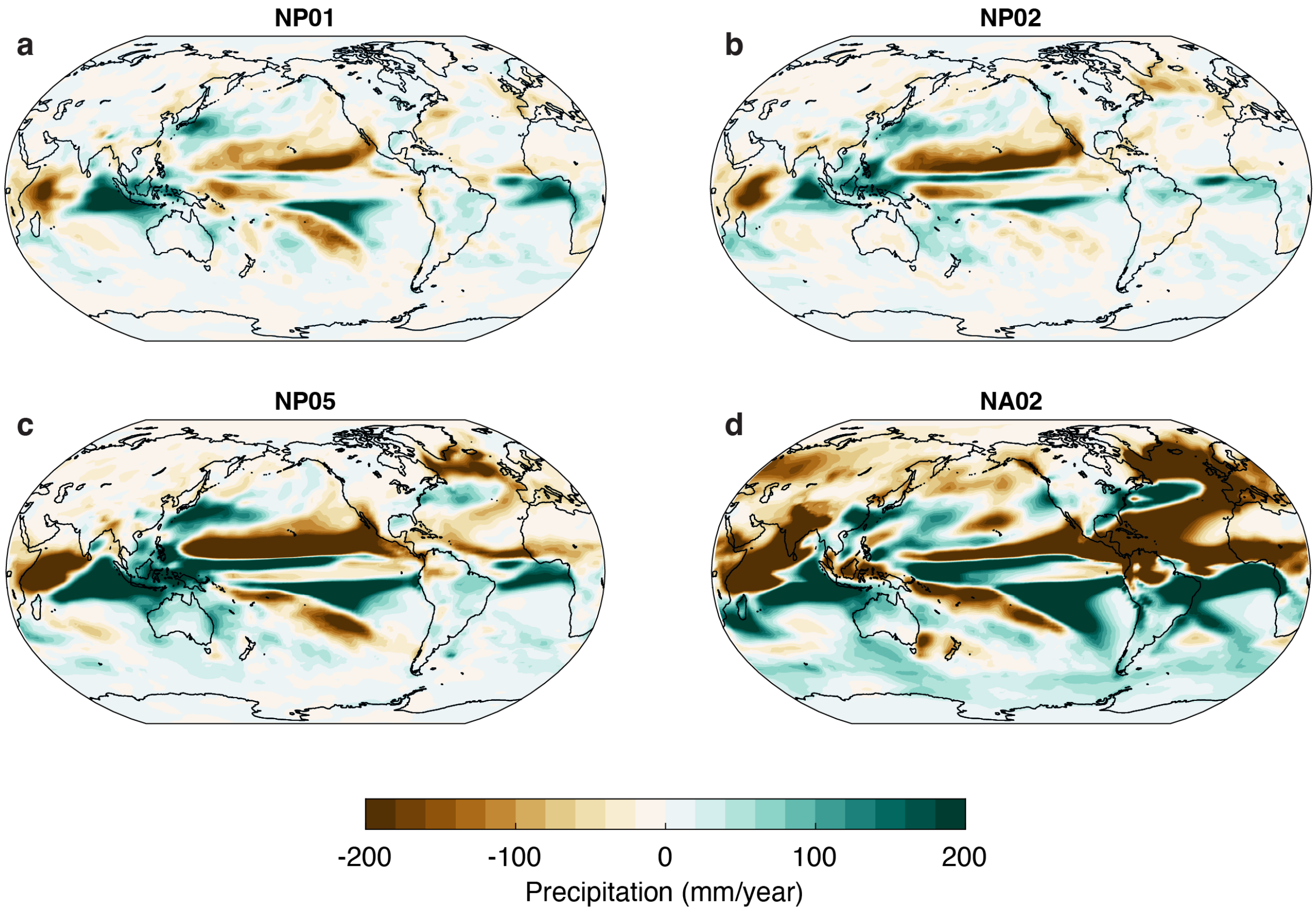

Figure S14 | Changes in mean annual precipitation (mm/year) in hosing simulations relative the 19 ka control. (a) NP01, **(b)** NP02, **(c)** NP05, and **(d)** NA02. Maps were generated using the m_map mapping package for MATLAB [9] and the Climate Data Toolbox for MATLAB [10].

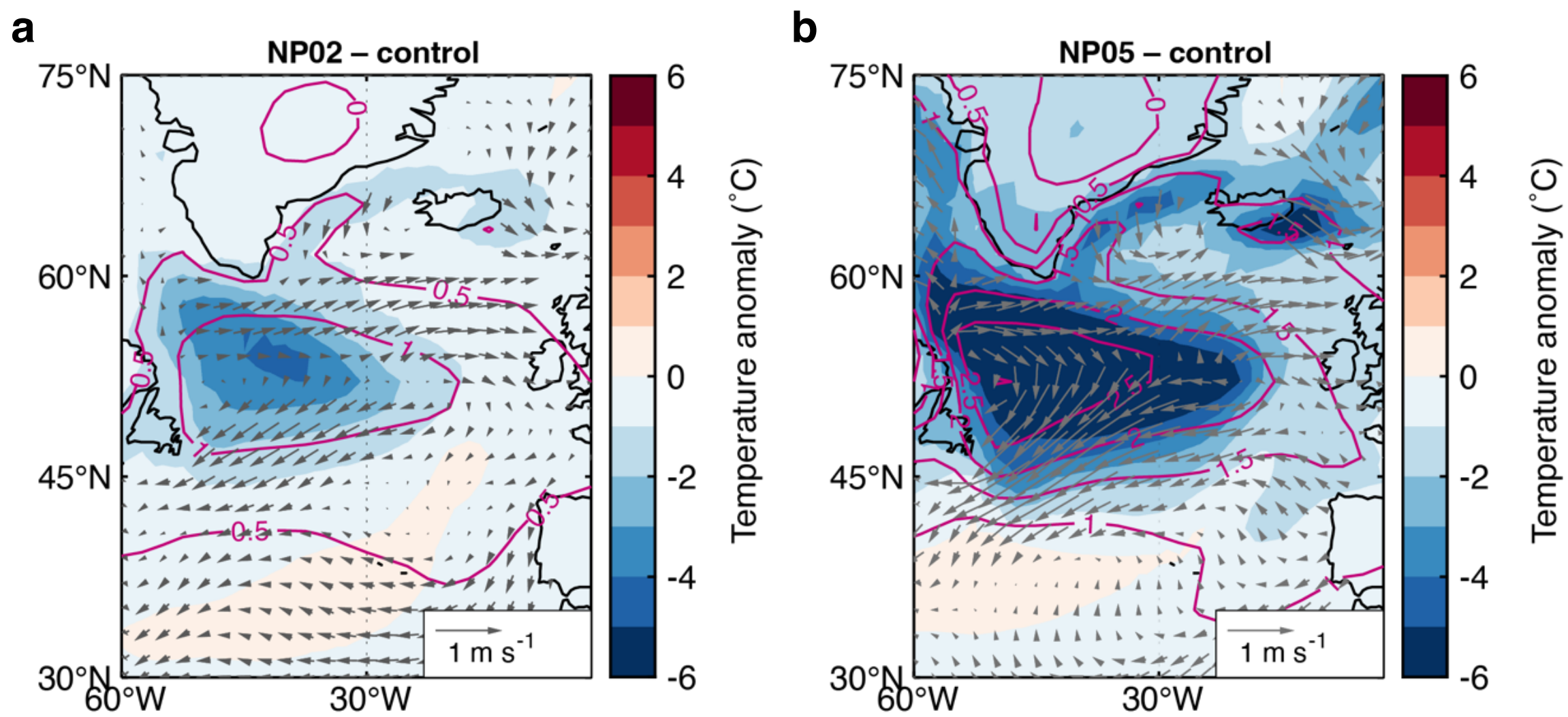

Figure S15 | Atmospheric processes driving further weakening of the North Atlantic subpolar gyre (SPG). Changes in surface temperature (shadings), sea-level pressure (contours), and 1000 hPa wind (vectors) in **(a)** NP02 and **(b)** NP05 relative to the 19 ka control, showing a high-pressure anomaly due to northern North Atlantic cooling driving clockwise wind anomaly to further weaken the SPG. Maps were generated using the m_map mapping package for MATLAB [9] and the Climate Data Toolbox for MATLAB [10].

SUPPLEMENTARY TABLES

Table S1. List of simulations reported in this study

ID	Hosing region	Boundary conditions	Freshwater flux	Total length
Control	N/A	N/A	N/A	19.05–19 ka
ctrl_ext	N/A	iTRACE 19 ka	N/A	200 years
NP01	45–70°N, 160–235°E	iTRACE 19 ka	0.1 Sv	400 years
NP02	45–70°N, 160–235°E	iTRACE 19 ka	0.2 Sv	400 years
NP05	45–70°N, 160–235°E	iTRACE 19 ka	0.5 Sv	400 years
NP02_BIIS01	0–15°W, 50–60°N	NP02 Year 400	0.1 Sv	200 years
NP02_BIIS005	0–15°W, 50–60°N	NP02 Year 400	0.05 Sv	200 years
ctrl_BIIS01	0–15°W, 50–60°N	iTRACE 19 ka	0.1 Sv	200 years
ctrl_BIIS005	0–15°W, 50–60°N	iTRACE 19 ka	0.05 Sv	200 years
NA02	80˚W–20˚E, 50–70˚N	iTRACE 19 ka	0.2 Sv	200 years

Table S2. List of $\delta^{18}O_{sw\text{-}ivc}$ records included in Fig. 3

Site number	Core name	Longitude	Latitude	Reference
NE Pacific				
1	EW0408-85JC	-144.20	59.60	from Praetorius et al. [11]
2	EW0408-66JC	-137.20	58.50	from Praetorius et al. [11]
3	EW0408-26JC	-136.70	57.60	from Praetorius et al. [11]
4	MD02-2496	-127.00	49.00	from Praetorius et al. [11]
5	JT96-09PC	-126.90	48.90	from Praetorius et al. [11]
6	ODP 1019	-124.90	41.70	from Praetorius et al. [11]
NW Pacific				
7	SO201-2-101KL	170.69	58.88	Riethdorf et al. [12]
8	SO201-2-77KL	170.70	56.33	Riethdorf et al. [12]
9	SO201-2-12KL	162.38	53.99	Riethdorf et al. [12]
10	LV29-114-3	152.88	49.38	Riethdorf et al. [12]
Indo-Pacific				
11	MD97-2141	121.30	8.80	Rosenthal et al. [13]
12	MD06-3075	125.83	6.48	Fraser et al. [14]
13	CG2	110.15	6.39	Huang et al. [15]
14	MD98-2181	125.83	6.30	Stott et al. [16]
15	SO189-119KL	96.32	3.52	Mohtadi et al. [17]
16	SO217-18522	119.08	1.40	Schröder et al. [18]
17	SO189-39KL	99.90	-0.78	Mohtadi et al. [17]
18	TGS-931	122.62	-2.41	Schröder et al. [18]
19	SO217-18526	118.17	-3.62	Schröder et al. [18]
20	SO217-18515	119.36	-3.63	Schröder et al. [18]
21	MD98-2165	118.34	-9.65	Levi et al. [19]
22	MD01-2378	121.79	-13.08	Xu et al. [20]
Agulhas				
23	GIK16160-3	37.87	-18.24	Wang et al. [21]
24	CD154 10-06P	32.15	-31.17	Simon et al. [22]
25	MD02-2594	17.34	-34.71	Dyez et al. [23]

SUPPLEMENTARY REFERENCES

1. Rousselet, L., Cessi, P. & Forget, G. Routes of the upper branch of the Atlantic meridional overturning circulation according to an ocean state estimate. *Geophysical research letters* **47**, e2020GL089137 (2020).
2. Walczak, M. H. *et al.* Phasing of millennial-scale climate variability in the Pacific and Atlantic Oceans. *Science* **370**, 716-720 (2020).
3. Koul, V. *et al.* Unraveling the choice of the north Atlantic subpolar gyre index. *Scientific Reports* **10**, 1005 (2020).
4. Moreno-Chamarro, E., Zanchettin, D., Lohmann, K. & Jungclaus, J. H. An abrupt weakening of the subpolar gyre as trigger of Little Ice Age-type episodes. *Climate Dynamics* **48**, 727-744 (2017).
5. Klockmann, M., Mikolajewicz, U., Kleppin, H. & Marotzke, J. Coupling of the subpolar gyre and the overturning circulation during abrupt glacial climate transitions. *Geophysical Research Letters* **47**, e2020GL090361 (2020).
6. Hátún, H., Sandø, A. B., Drange, H., Hansen, B. & Valdimarsson, H. Influence of the Atlantic Subpolar Gyre on the Thermohaline Circulation. *Science* **309**, 1841-1844 (2005). https://doi.org:doi:10.1126/science.1114777
7. Born, A. & Stocker, T. F. Two Stable Equilibria of the Atlantic Subpolar Gyre. *Journal of Physical Oceanography* **44**, 246-264 (2014). https://doi.org:https://doi.org/10.1175/JPO-D-13-073.1
8. Häkkinen, S., Rhines, P. B. & Worthen, D. L. Atmospheric Blocking and Atlantic Multidecadal Ocean Variability. *Science* **334**, 655-659 (2011). https://doi.org:doi:10.1126/science.1205683
9. Pawlowicz, R. M_Map: A mapping package for MATLAB. *Computer Software* **765** (2020).
10. Greene, C. A. *et al.* The climate data toolbox for MATLAB. *Geochemistry, Geophysics, Geosystems* **20**, 3774-3781 (2019).
11. Praetorius, S. K. *et al.* The role of Northeast Pacific meltwater events in deglacial climate change. *Science Advances* **6**, eaay2915 (2020).
12. Riethdorf, J. R., Max, L., Nürnberg, D., Lembke-Jene, L. & Tiedemann, R. Deglacial development of (sub) sea surface temperature and salinity in the subarctic northwest Pacific: Implications for upper-ocean stratification. *Paleoceanography* **28**, 91-104 (2013).
13. Rosenthal, Y., Oppo, D. W. & Linsley, B. K. The amplitude and phasing of climate change during the last deglaciation in the Sulu Sea, western equatorial Pacific. *Geophysical Research Letters* **30** (2003).
14. Fraser, N. Deglacial changes in West Pacific circulation: insights from benthic foraminiferal δ13C. *Late Pleistocene climate and circulation history of the western equatorial Pacific*, 86 (2015).
15. Huang, J. *et al.* Terrigenous supplies variability over the past 22,000 yr in the southern South China Sea slope: Relation to sea level and monsoon rainfall changes. *Journal of Asian Earth Sciences* **117**, 317-327 (2016).

16 Stott, L., Poulsen, C., Lund, S. & Thunell, R. Super ENSO and global climate oscillations at millennial time scales. *science* **297**, 222-226 (2002).
17 Mohtadi, M. *et al.* North Atlantic forcing of tropical Indian Ocean climate. *Nature* **509**, 76-80 (2014).
18 Schröder, J. F. *et al.* Deglacial warming and hydroclimate variability in the central Indonesian archipelago. *Paleoceanography and Paleoclimatology* **33**, 974-993 (2018).
19 Levi, C. *et al.* Low-latitude hydrological cycle and rapid climate changes during the last deglaciation. *Geochemistry, Geophysics, Geosystems* **8** (2007).
20 Xu, J., Holbourn, A., Kuhnt, W., Jian, Z. & Kawamura, H. Changes in the thermocline structure of the Indonesian outflow during Terminations I and II. *Earth and Planetary Science Letters* **273**, 152-162 (2008).
21 Wang, Y. V. *et al.* Northern and southern hemisphere controls on seasonal sea surface temperatures in the Indian Ocean during the last deglaciation. *Paleoceanography* **28**, 619-632 (2013).
22 Simon, M. H. *et al.* Salt exchange in the Indian-Atlantic Ocean Gateway since the Last Glacial Maximum: A compensating effect between Agulhas Current changes and salinity variations? *Paleoceanography* **30**, 1318-1327 (2015).
23 Dyez, K. A., Zahn, R. & Hall, I. R. Multicentennial Agulhas leakage variability and links to North Atlantic climate during the past 80,000 years. *Paleoceanography* **29**, 1238-1248 (2014). https://doi.org:https://doi.org/10.1002/2014PA002698
