# Supplementary material for: North Pacific meltwater weakens the Atlantic Meridional Overturning Circulation and preconditions Heinrich Stadial 1
Source: Nat Commun. 2026 Jul 4;17:8389. doi: 10.1038/s41467-026-75199-y (PMC13473526; doi:10.1038/s41467-026-75199-y)
Supplement: Supplementary file 2 [file 41467_2026_75199_MOESM2_ESM.pdf]

nature portfolio

Peer Review File

North Pacific meltwater weakens the Atlantic Meridional Overturning Circulation and preconditions Heinrich Stadial 1

Corresponding Author: Dr Chijun Sun

This file contains all reviewer reports in order by version, followed by all author rebuttals in order by version.

Version 0:

Reviewer comments:

Reviewer #1

(Remarks to the Author)
Review of Sun and others:
“Triggering of Heinrich events by Cordilleran Ice Sheet meltwater into the North Pacific”

Summary:
Using the complex Earth System Model (iCESM) and freshwater hosing experiments in the Northeast Pacific, the authors investigate the influence of Siku events (i.e., freshwater discharge events from the Cordilleran Ice Sheet) on changes in the Atlantic Ocean. They found that the freshwater signal from the Northeast Pacific can be transported to the North Atlantic via a westward Pacific-Indian-Atlantic pathway, leading to AMOC weakening and subsurface warming near the BIIS. With further freshwater release into the North Atlantic, additional reductions in the AMOC and increased subsurface warming over the northern North Atlantic are observed. Based on these findings, the authors suggest that Siku events may play an important role initiating AMOC weakening and further triggering Heinrich events through subsurface warming.

Main comments:
The processes discussed in the manuscript are intriguing, emphasizing the importance of a global perspective in paleoclimate studies, particularly regarding ice-ocean-atmosphere interactions across different ocean basins. The text is also clearly written. However, there are still some major concerns that need to be fully addressed before the manuscript can meet the requirements for publication.

1. The freshwater hosing experiments, which introduce 0.2 and 0.5 Sv of freshwater into the northeast Pacific, seem overly large when considering the potential contributions from freshwater perturbations originating from the Cordilleran Ice Sheet (CIS). Although the manuscript argues that these values are consistent with an estimated global sea-level rise of ~20 m attributed to the northern hemisphere, I question how much of this contribution can be attributed to the CIS. In comparison to the Laurentide ice sheet, the CIS was significantly smaller in size. Seguinot et al. (2016) estimate a sea-level equivalent of approximately 8 m for the CIS during the LGM. Moreover, around 18 ka, the CIS was still fully developed and in a glacial stage, making full deglaciation unlikely. And any oscillatory freshwater discharge during this period would have been much smaller.

2. Indeed, the exact freshwater hosing value and the associated ocean responses are likely model-dependent. Therefore, the sensitivity of AMOC responses to freshwater perturbations – such as those introduced directly over the middle of the North Atlantic (e.g., the IRD belt) – should be presented as a reference and discussed in greater detail.

3. In the results and discussion section, the authors state that their findings “underscore the sensitivity of freshwater hosing experiments to the Atlantic Ocean’s initial conditions.” However, this needs to be presented and discussed in greater detail. In their model setup, they describe only one experiment BIIS01, which was continued from the experiment NP02. To support their conclusions, a more thorough analysis incorporating experiments initialized with different ocean conditions and varying hosing values should be conducted.

4. The model only discusses the influences of the ocean. However, since the manuscript aims to link the Siku events with global climate effects and subsequent Heinrich events, it is also essential to address the atmospheric responses, the resulting surface mass balance changes on the ice sheets, and potential impacts on ice sheet dynamics (e.g. Heinrich

events). As part of the Earth system, I believe that atmospheric responses are also crucial to the land-based northern hemisphere ice sheets.

5. This study is based on one mechanism for triggering Heinrich events, with ocean subsurface warming identified as the key driver. However, various other mechanisms have been proposed, such as the binge-purge mechanism. Additionally, a recent study (Schannwell et al., 2024) proposes that multiple DO events could lead to phasing locking with Heinrich events. It would be necessary to include more discussions of these alternative theories.

Specific comments:

Line 74-79: Why specifically 0.2 and 0.5 Sv? As is mentioned earlier, Seguinot et al. (2016) estimates a sea level equivalent of approximately 8 meters for the Cordilleran Ice Sheet during the LGM. Based on this, I would expect a smaller freshwater input associated with Siku events. Did you also test the response to, for example, 0.1 Sv?

Line 115-127: This paragraph, along with the associated discussion on present-day water mass transportation, appears out of place. It lacks a smooth transition and does not align with the logical flow of the current section. I recommend rephrasing it for better integration or moving it to the discussion section, where it may be more relevant.

Line 129-135, 185-189. In this section, the author propose that an initial AMOC weakening could be largely amplified by the sequential freshwater discharges from the BIIS or LIS. Therefore, it is essential to compare AMOC responses without the initial AMOC weakening. More specifically, add a reference simulation analogous to BIIS01, but initialized from the 19 ka control rather than NP02, would provide a more robust comparison.

Line 151-152, 157-159: I question to what extent the retreat of the BIIS is driven by subsurface warming resulting from the initial AMOC weakening, since the ocean interface of the BIIS is rather small. While the authors estimate a 2.5 m global sea-level change, I am skeptical about how much of this contribution specifically originates from the BIIS. Clarification or additional evidence to support this attribution would strengthen the argument.

Line 164-165: Following the previous comment, conducting additional hosing experiments with varying freshwater fluxes would be essential to evaluate the sensitivity and responses of the AMOC more comprehensively. This would provide a clearer understanding of the potential range of impacts caused by different magnitudes of freshwater input.

Line 209-210: The authors briefly mention atmospheric (or previously climate) responses; however, there is no detailed discussion on this aspect. Expanding on the atmospheric responses, and impacts on the surface mass balance changes are essential, as the Northern Hemisphere ice sheets are largely influenced by atmospheric processes.

Fig. S9: Since this is a key part of the main conclusion of the manuscript, it would be better to move this figure into the main text for better integration and emphasis.

References:

Schannwell, C., Mikolajewicz, U., Kapsch, M. L., & Ziemen, F. (2024). A mechanism for reconciling the synchronisation of Heinrich events and Dansgaard-Oeschger cycles. Nature Communications, 15(1), 2961.

Seguinot, J., Rogozhina, I., Stroeven, A. P., Margold, M., & Kleman, J. (2016). Numerical simulations of the Cordilleran ice sheet through the last glacial cycle. The Cryosphere, 10(2), 639-664.

Reviewer #2

(Remarks to the Author)
Review Nat Comm: Chijun Sun et al. (2025).

Heinrich events are cold periods during the (last) deglaciation associated with iceberg meltwater release from the Laurentide ice sheet, observed in the North Atlantic. Siku events are observed in the North Pacific and are associated with iceberg melting from the Cordilleran ice sheet. This study investigates the influence of Siku events on the occurrence of Heinrich events. It is an interesting study because the influence of freshwater feedbacks on the climate system and the ocean in specific are often omitted. In the ideal world, these freshwater fluxes come from dynamic ice sheet models in order to have realistic numbers.

This study explains the occurrence of Heinrich events as a chain reaction where a Siku event in the North Pacific leads to ice shelf melting of the British Irish Ice Sheet (BIIS), the resulting freshwater from the BISS would further weaken the AMOC and lead to subsurface warming in the subpolar North Atlantic. I find it an interesting study with a bold conclusion based on one extended experiment. I believe these findings could be better supported if some additional simulations would be performed, which I further detail below.

The simulations are 400 years long. One additional experiments is 600 years long, where an additional freshwater flux from the BIIS is added at model year 400 when the freshwater flux in the North Pacific is halted. It would be interesting to have a

control run where no additional freshwater is added during the entire length of the simulations (600 years) and another run where the freshwater flux in the North Pacific is halted at year 400 and no additional freshwater flux is added from the BIIS to see whether the AMOC recovers. Also, only one additional run is performed as a continuation from NP02. It would be interesting whether the simulations show the same results for other meltwater releases (NP05 or another additional freshwater release experiment).

Line by line comments:

L74-75: How did you choose the values of 0.2 and 0.5 Sv and why are these numbers exactly chosen?

L78-79: It is indeed true that a sea-level rise of 18.8 m in 400 years is a realistic number during the peak of the deglaciation. However, this number comes mainly from 4 main ice masses (North American ice sheet, Fennoscandian ice sheet, Greenland ice sheet and the Antarctic ice sheet). It is estimated that the North American ice sheet (Cordilleran ice sheet and Laurentide ice sheet combined) had a volume equivalent to ~60-80m sea level, or ~45-60% of the 130 m sea-level lowering at the Last Glacial Maximum. It is thought that the Cordilleran ice sheet disappeared fast – in about 4000 years. Given that the Cordilleran ice sheet is the smaller one, a sea-level rise equivalent up to 18.8 m in 400 years is high. Do you assume that the Laurentide ice sheet also partly drained into the North Pacific?

L81-82: Say how you would investigate the causes of AMOC weakening in this study instead of giving a result here.

L99: Why does figure S6 appear in the text before you first mention S3, S4 or S5? Please order the figures chronological by first appearance in the text.

L166: Why did you decide to run an additional experiments where freshwater is released from the BIIS only for the NP02 scenario and not for the NP05 scenario?

L181: Why did you not perform a run where you only have meltwater from the BIIS without precursor meltwater release from a Siku event? You have all the modelling tools in place not to make speculations and hypothesis whether the freshwater forcing could be sufficient.

Reviewer #3

(Remarks to the Author)
Review notes on ‘Triggering of Heinrich Events by Cordilleran Ice Sheet Meltwater into the North Pacific’ by Sun et al.

Sun et al., perform a series of freshwater hosing experiments to investigate possible connections between Northeast Pacific Siku events and North Atlantic Heinrich events using CESM1.3, which uses an ocean model with 1 degree horizontal resolution. Based on the results of their simulations, the authors suggest that Heinrich Event 1 could have been triggered by Siku Event 1.

Their results can be broken down into two main sections:

(1) The addition of freshwater—either 0.2 Sv or 0.5 Sv over 400 years—into the Northeast Pacific causes AMOC to weaken because of a decrease in the salinity and thus density of North Atlantic surface waters. Since the Bering Strait was closed at 19 ka BP, the authors suggest that this freshwater reaches the North Atlantic via the Indonesian Throughflow and Agulhas Leakage. The authors also rule out local changes in hydroclimate because there is a decreasing trend in precipitation over the North Atlantic.

(2) While these initial hosing experiments lead to a weakening of the AMOC, warming of the subsurface is restricted to latitudes south 50N, which is inconsistent with the classic view that warming of the northern North Atlantic subsurface causes Heinrich Events by triggering Laurentide Ice Sheet collapse. Consequently, the authors suggest (and then test this hypothesis with an additional hosing experiment [0.1 Sv released into the Northeast Atlantic over 200 years]) that subsurface warming in the Northeast Atlantic, due a weaker AMOC, caused the British Irish Ice Sheet to melt, releasing additional freshwater in the North Atlantic, which was sufficient to warm the northern North Atlantic surface and trigger a collapse of the Laurentide Ice Sheet consistent with Heinrich Events.

I thoroughly enjoyed reading this well-considered and well-written study, which applies a relatively novel concept to address a long-standing question. I am generally supportive of its eventual publication, provided the authors address the concerns and suggestions outlined below, especially my major concern.

Main comments:

My major concern with the study is that is in unclear why this new and relatively complicated Siku mechanism is needed as a trigger for Heinrich Events since the Bassis et al., (2017) hypothesis means we can explain Heinrich Events as paced by isostatic rebound linked to DO type events caused by cyclical changes in the Nordic Seas. Therefore, it is crucial that early on in the manuscript, the authors acknowledge that although plausible/viable mechanisms for explaining Heinrich Events

exist, e.g., Bassis et al., 2017, this study wants to explore the potential, but not necessarily requisite, role of Pacific freshwater in Heinrich Events.

I also have concerns that this study is not explicit enough in addressing the major limitations, assumptions, and caveats associated with the study's experimental design.

For example, while the added freshwater flux, equivalent to 7.5m and 18.8m, is within bounds of total global sea level rise, it implies that nearly all global sea level rise was due to freshwater flux from the Cordilleran and/or Pacific sector of the LIS. However, previous work (e.g., Clark et al., 2004; Carlson & Clark, 2012; Toucanne et al., 2015) has argued for substantial amounts of melt at 18-19 ka BP from the BIIS and FIS, which is incompatible with the prescribed flux freshwater into the Northeast Pacific associated with Siku 1. Please address this inconsistency by including some additional text discussing the feasibility of the volumes of freshwater fluxes used in these hosing experiments with respect to previous work, i.e., the references included above.

Additional text is also required acknowledging that the hypothesised mechanistic relationship between Siku 1 and Heinrich 1 may not be valid for other earlier Heinrich Events, both in terms of the differing volumes freshwater flux (and global sea level rise) and variable boundary conditions during the last glacial period. For example, previous work, e.g., Hemming et al., 2004 (and references therein) & Roberts et al., 2014, suggests that during earlier Heinrich stadials there wasn't 15+ metres of sea level change. Would the volume of freshwater equivalent to a much smaller global sea level rise also result in the same sequence of downstream events leading to a Heinrich Event (e.g., subsurface warming in the Northeast Atlantic, BIIS melt etc)? This should be discussed explicitly within the text, including some acknowledgement that the prescribed volume of freshwater fluxes used in these hosing experiments is not applicable to all Heinrich Events. Similarly, there also needs to be more discussion of the varying boundary conditions during the last glacial period and how this impacts the proposed Northeast Pacific-North Atlantic connection. For example, it has been suggested that the Bering Strait was open from 46-35.7 ka BP (Farmer et al., 2022), so how would this have impacted the freshwater flux associated with Siku 4? Both these points raise the issue that the proposed mechanism is only applicable to Siku 1/Heinrich 1, and this must be acknowledged within the main text.

Although a 1 degree resolution ocean model is good, it is not as high as some state of the art ocean models used in similar studies. Does model resolution have any impact on freshwater routing/spreading pathways? Can you leverage the results from other modeling studies looking at spreading pathways in the ocean, e.g., Condron et al., 2009, to help make the case the freshwater routing in this study is robust and not model dependent? Please include an additional paragraph discussing how simulations using a higher resolution model may (or may not) impact the results of this study. This paragraph should also include a sentence acknowledging that the impact of freshwater on AMOC is dependent on model resolution, e.g., Shan et al., 2024.

Invoking the BIIS as an intermediary step is well argued and supported with hosing experiment BIIS01, however, after that, the mechanism is a bit more ad hoc and there is no consideration given to the potential impact of additional FIS melt and associated freshwater input into the northern North Atlantic. Please make this section clearer and include some additional text acknowledging other potential impacts and uncertainties associated with BIIS melt.

Since the Bering Strait was closed at 19 ka BP, the authors suggest that freshwater from S1 reaches the North Atlantic via the Indonesian Throughflow and Agulhas Leakage. While this routing is consistent with modelling/observational data and supported by planktic d18Osw records from the southern tip of Africa, more paleo evidence from other sites along the proposed flow path are necessary to fully constrain this long and complex routing, e.g., data from the Pacific Ocean, Indian Ocean, and/or South Atlantic. Or if such records are not available, then this should be acknowledged in the text, e.g., 'paleo evidence in support of this freshwater routing is limited to the southern tip of Africa'.

Related to this, the main text would benefit from a more comprehensive description of how freshwater originating in the Northeast Pacific reaches the subpolar North Atlantic. At present the description is limited to 2/3 lines in the main text, with some additional information in the SI, the latter of which contains some really useful context for the reader. Given the relative brevity of the manuscript (which is definitely not a bad thing), can you incorporate this inter-basin freshwater transport text into the main text?

Thanks for a really fascinating study!

Line by line comments:

L38: 'variability' instead of 'strength' might be better?
L39: Include a reference to the RAPiD array at 26.5oN, e.g., https://www.bodc.ac.uk/data/published_data_library/catalogue/10.5285/223b34a3-2dc5-c945-e063-7086abc0f274/
L55/56: Unclear, please reword, e.g., …'similar to the observed retreat of the West Antarctic ice today'.
L91-94: Where is the evidence for a decreasing trend in precipitation over the North Atlantic?
L102: Not sure I agree with the suggested positive feedback though – would cooling not also increase the density of surface waters, thereby sustaining/facilitating deep water formation?
L116-117: '…represents a robust physical process supported by other modeling studies as well as modern observations of surface water mass transport'. It looks like you refer to the modern observational data before the climate modeling work, so flip the order to reflect that in this set-up sentence.
L117: Can you also be a bit more specific about the source of these conclusions, e.g., at least mention the use of ECCOv4?

L120: I think the point here is that the Rousselet et al., 2020 study is supportive of an Indonesian Throughflow origin, but is there any evidence that these waters originated in the Northeast Pacific?
L124: What model? Can you be more specific again please?
L144: Missing a 'the', e.g., 'warms the northern North Atlantic'.
L146: 'cooling prevails at both surface and subsurface' should be 'cooling prevails in both the surface and subsurface'.
L194: This feels more like a conclusion then implications section rather than a discussion

References:

Clark, P. U., McCabe, A. M., Mix, A. C., & Weaver, A. J. (2004). Rapid rise of sea level 19,000 years ago and its global implications. Science, 304(5674), 1141-1144.

Carlson, A. E., & Clark, P. U. (2012). Ice sheet sources of sea level rise and freshwater discharge during the last deglaciation. Reviews of Geophysics, 50(4).

Toucanne, S., Soulet, G., Freslon, N., Jacinto, R. S., Dennielou, B., Zaragosi, S., ... & Bayon, G. (2015). Millennial-scale fluctuations of the European Ice Sheet at the end of the last glacial, and their potential impact on global climate. Quaternary Science Reviews, 123, 113-133.

Hemming, S. R. (2004). Heinrich events: Massive late Pleistocene detritus layers of the North Atlantic and their global climate imprint. Reviews of Geophysics, 42(1).

Roberts, W. H., Valdes, P. J., & Payne, A. J. (2014). A new constraint on the size of Heinrich Events from an iceberg/sediment model. Earth and Planetary Science Letters, 386, 1-9.

Farmer, J. R., Pico, T., Underwood, O. M., Cleveland Stout, R., Granger, J., Cronin, T. M., ... & Sigman, D. M. (2023). The Bering Strait was flooded 10,000 years before the last glacial maximum. Proceedings of the National Academy of Sciences, 120(1), e2206742119.

Condron, A., Winsor, P., Hill, C., & Menemenlis, D. (2009). Simulated response of the Arctic freshwater budget to extreme NAO wind forcing. Journal of Climate, 22(9), 2422-2437.

Shan, X., Sun, S., Wu, L., & Spall, M. (2024). Role of the Labrador Current in the Atlantic Meridional Overturning Circulation response to greenhouse warming. Nature Communications, 15(1), 7361.

Reviewer #4

(Remarks to the Author)
I co-reviewed this manuscript with one of the reviewers who provided the listed reports. This is part of the Nature Communications initiative to facilitate training in peer review and to provide appropriate recognition for Early Career Researchers who co-review manuscripts.

Version 1:

Reviewer comments:

Reviewer #1

(Remarks to the Author)
Main comments:
This is my second review of "Triggering of Heinrich events by Cordilleran Ice Sheet meltwater into the North Pacific" by Sun and others. I thank the authors for incorporating the comments into the revised manuscript. My major concern related to the magnitude of freshwater hosing associated with ice sheet dynamics. While the authors argue that the current generation of general circulation models is known to produce an overly stable AMOC, which may be a fair point.

Overall, I do find this to be a valuable study from a mechanistic and global perspective. The work highlights the complexity of the Earth system by demonstrating how ice-sheet changes in different regions can generate interconnected climate response (mainly ocean responses here) across multiple ocean basins. However, since this study does not explicitly simulate the triggering of the BIIS or Heinrich event 1 associated with ice sheet dynamics, I would be cautious about making definitive statements regarding "triggering the Heinrich events" (for example, Lines 69-70, Lines 99-100, Line 163 …). It may be more appropriate to focus on the mechanism related to North Atlantic subsurface warming. Other mechanisms proposed to trigger Heinrich events cannot be ruled out.

Line 137-152: The meltwater transport pathway and the comparison with proxy evidence. I appreciate the efforts. However, it remains unclear to what extent the signals can be attributed to the S1 event: (1) the maxima don't match between the IRD and the average NE and NW Pacific records (Fig. 3a,c,d) during 20-17 ka; decreases in the freshwater proxy signal occur earlier than the S1 event. (2) phase changes among different proxy records also differ substantially. The response times indicated by modelling across basins are on the order of tens to hundreds of years, whereas such delays are not evident in

the proxy records. (3) there appears to be a strong signal during the YD in the NE pacific record. What is the cause of this signal? Could another Siku event have occurred during this period? If so, why do the Indo-Pacific records exhibit opposite phase changes? Overall, during the deglaciation period, changes in these delta18O records could be complex, given the large changes in orbital configuration, greenhouse gas concentrations, North Hemisphere ice sheets, and associated large-scale precipitation and hydroclimate changes. This needs to be interpreted carefully and should not be attributed solely to the Siku event.

Line 157-161: "… reconciling the conundrum that AMOC weakening occurred prior to H1 without the presence of Laurentide meltwater discharge". Not sure about this statement. In your NP02 experiment, the AMOC strength decreases to about 21 Sv, which is still quite vigorous, whereas in NP05, the freshwater input of 0.5 Sv seems excessively large. Moreover, increasingly recent studies suggest that millennial-scale oscillations may arise from changes in boundary conditions, such as orbit-CO2-ice sheet configurations, and this cause during the studied period cannot be ruled out. Additionally, during 19-17ka, insolation was already quite high, making it highly likely that the Eurasian ice sheets (including not only the BIIS but also the Fennoscandian and Barents-Kara Sea ice sheets) began to melt (e.g., Patton et al., 2017), which should also not be ignored.

Other comments:

Line 69-70: This sentence sounds weird. The precursory subsurface warming is one of the proposed mechanisms; describing it as "satisfactory" maybe too subjective.

Line 108-111: "precipitation decreases over the northern North Atlantic NADW formation site". How does continental runoff into the North Atlantic change? Which could potentially also be linked with atmospheric mechanisms.

Reference:
Patton, H., Hubbard, A., Andreassen, K., Auriac, A., Whitehouse, P. L., Stroeven, A. P., ... & Hall, A. M. (2017). Deglaciation of the Eurasian ice sheet complex. Quaternary Science Reviews, 169, 148-172.

Reviewer #2

(Remarks to the Author)
The authors have carefully addressed my major comments. A discussion on the freshwater release corresponding to a certain ice sheet volume and the possible magnitude of this ice sheet volume has been properly addressed for both the Cordilleran Ice Sheet and the British-Irish Ice Sheet. The author's also corresponded to my request for additional simulations. Therefore I would like to support this preprint for publication in Nature Communications.

Reviewer #3

(Remarks to the Author)
The authors have done an excellent job addressing all of my comments. I am particularly impressed with the inclusion of the additional model runs and thorough proxy compilation, as well as their thoughtful responses. Therefore, I recommend publication of this paper.

Apart from a couple of small editorial suggestions, my only very minor point is that the authors should include a line in the main text acknowledging the use of exaggerated freshwater forcing and signposting the methods section that contains the justification, i.e., that the use of exaggerated freshwater forcing is valid due to AMOC's over-stability in GCMs. I think this point is well-argued, and a quick acknowledgment would be useful, as the freshwater volumes are quite striking on first read, and I assume would be for most paleoceanographers.

L61: Consider using 'hinges' instead of 'would hinge'.

L137: The phrase/argument '…by proxy evidence' could be strengthened by highlighting the spatial extent of the proxy compilation. It's not just a couple of sites; it's much more impressive than that, e.g., …by proxy evidence from multiple sites in the Pacific, Indonesian Throughflow and south of Africa.

Reviewer #4

(Remarks to the Author)
I co-reviewed this manuscript with one of the reviewers who provided the listed reports. This is part of the Nature Communications initiative to facilitate training in peer review and to provide appropriate recognition for Early Career Researchers who co-review manuscripts.

Version 2:

Reviewer comments:

Reviewer #1

(Remarks to the Author)
This is my third review of “Triggering of Heinrich events by Cordilleran Ice Sheet meltwater into the North Pacific” by Sun and others. I thank the authors for incorporating my comments into the revised manuscript, including changing the title. I only have one more comment:

Line 74-75:”these mechanisms are more relevant for Heinrich stadials at glacial terminations or inceptions rather than all Heinrich stadials…” I do not agree with this statement. A number of recent studies have examined DO-type oscillations during MIS 3, when climate conditions were intermediate, for example Zhang et al. (2021, NG) and Vettoretti et al. (2022, NG). Please revise this sentence.

Reference:
Zhang, X. et al. Direct astronomical influence on abrupt climate variability. Nat. Geosci. 14, 819–826 (2021).
Vettoretti, G., Ditlevsen, P., Jochum, M. & Rasmussen, S. O. Atmospheric CO2 control of spontaneous millennial-scale ice age climate oscillations. Nat. Geosci. 15, 300–306 (2022).

Open Access This Peer Review File is licensed under a Creative Commons Attribution 4.0 International License, which permits use, sharing, adaptation, distribution and reproduction in any medium or format, as long as you give appropriate credit to the original author(s) and the source, provide a link to the Creative Commons license, and indicate if changes were made.
In cases where reviewers are anonymous, credit should be given to 'Anonymous Referee' and the source.
The images or other third party material in this Peer Review File are included in the article’s Creative Commons license, unless indicated otherwise in a credit line to the material. If material is not included in the article’s Creative Commons license and your intended use is not permitted by statutory regulation or exceeds the permitted use, you will need to obtain permission directly from the copyright holder.
To view a copy of this license, visit https://creativecommons.org/licenses/by/4.0/

Response to Reviewer #1

Reviewer #1 (Remarks to the Author):

Review of Sun and others:
"Triggering of Heinrich events by Cordilleran Ice Sheet meltwater into the North Pacific"

Summary:
Using the complex Earth System Model (iCESM) and freshwater hosing experiments in the Northeast Pacific, the authors investigate the influence of Siku events (i.e., freshwater discharge events from the Cordilleran Ice Sheet) on changes in the Atlantic Ocean. They found that the freshwater signal from the Northeast Pacific can be transported to the North Atlantic via a westward Pacific-Indian-Atlantic pathway, leading to AMOC weakening and subsurface warming near the BIIS. With further freshwater release into the North Atlantic, additional reductions in the AMOC and increased subsurface warming over the northern North Atlantic are observed. Based on these findings, the authors suggest that Siku events may play an important role initiating AMOC weakening and further triggering Heinrich events through subsurface warming.

Main comments:
The processes discussed in the manuscript are intriguing, emphasizing the importance of a global perspective in paleoclimate studies, particularly regarding ice-ocean-atmosphere interactions across different ocean basins. The text is also clearly written. However, there are still some major concerns that need to be fully addressed before the manuscript can meet the requirements for publication.

We thank Reviewer #1 for the supportive feedback and helpful comments. We have carefully addressed all the concerns. Please find our responses to each comment below.

1. The freshwater hosing experiments, which introduce 0.2 and 0.5 Sv of freshwater into the northeast Pacific, seem overly large when considering the potential contributions from freshwater perturbations originating from the Cordilleran Ice Sheet (CIS). Although the manuscript argues that these values are consistent with an estimated global sea-level rise of ~20 m attributed to the northern hemisphere, I question how much of this contribution can be attributed to the CIS. In comparison to the Laurentide ice sheet, the CIS was significantly smaller in size. Seguinot et al. (2016) estimate a sea-level equivalent of approximately 8 m for the CIS during the LGM. Moreover, around 18 ka, the CIS was still fully developed and in a glacial stage, making full deglaciation unlikely. And any oscillatory freshwater discharge during this period would have been much smaller.
We acknowledge that the imposed meltwater forcing in NP02 and especially NP05 may have overestimated the actual meltwater contribution from the Cordilleran Ice Sheet during S1, given that the total Cordilleran ice sheet volume is estimated to be ~8.8 m s.l.e (Seguinot et al., 2016) and that melt from other ice sheets also contributed to the global

sea-level change at 19 ka. However, we would like to argue that such exaggerations can be justified because the current generation of general circulation models is known to produce an overly stable AMOC (Valdes, 2011). For example, while H1 meltwater release from the Laurentide Ice Sheet has been estimated at only ~0.02 Sv (Zhou & McManus, 2024), substantially stronger forcings (0.25 to 1 Sv) are typically required to achieve atmospheric and oceanic responses that are consistent with proxy data (DiNezio et al., 2025; Lawman et al., 2025; Pöppelmeier et al., 2023). The models' over-stability may result from model biases in surface water salinity in the South Atlantic, which cause the models to have a net import of freshwater in the South Atlantic, contradicting the net export shown in observational data (Weijer et al., 2019). This bias salinifies the Atlantic Ocean when the AMOC is weakened, internally reinvigorating the AMOC when the external forcing is too small (Weijer et al., 2019). In this context, our slight exaggeration of freshwater forcing is not only justified, but also allows us to test the sensitivity and robustness of the AMOC's response to varying forcing intensities.

Furthermore, we performed an additional experiment, with 0.1 Sv of freshwater added to the North Pacific (3.8 m sea level equivalent) following Reviewers' suggestions. This simulation shows qualitatively similar responses across NP01, NP02, and NP05, including freshwater transport pathway (Fig. 2; copied below) and North Atlantic subsurface temperature change (Fig. 4a-c; copied below). This suggests that the mechanisms related to the impact of North Pacific freshwater proposed in our study are robust and insensitive to hosing strength.

Please see our revised Methods section in line 357-390.

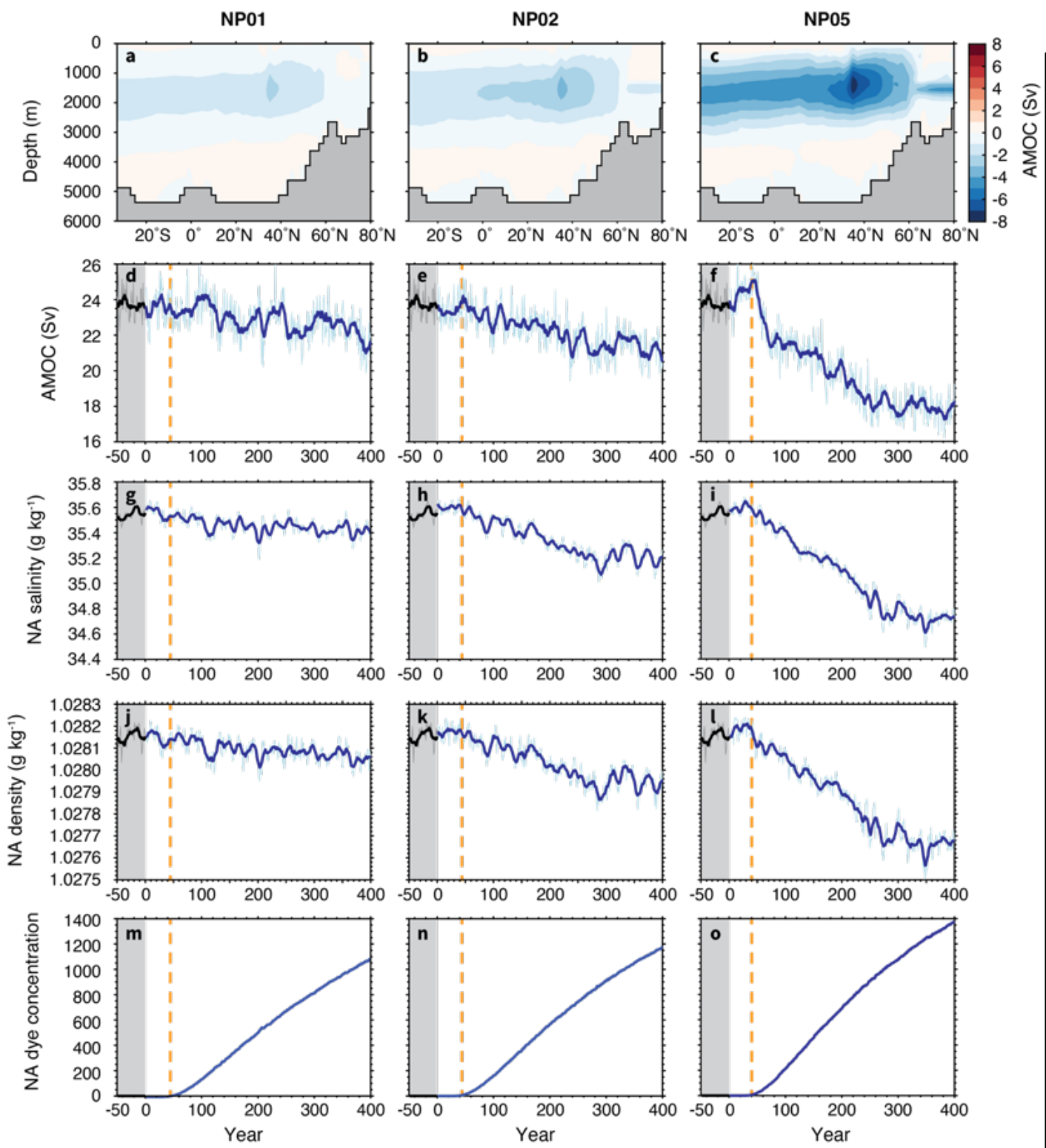

Figure 2 | Relationship between AMOC weakening and freshwater transport from the North Pacific. a-c) Changes in AMOC strength. **d-f)** Evolution of AMOC strength (defined as the maximum stream function between 10 and 60 ° N). **g-i)** Evolution of upper water (top 100 m) salinity in the North Atlantic (45-60°N). **j-l)** Evolution of upper water density in the North Atlantic. **m-o)** Dye tracer concentration in the North Atlantic upper water, indicative of water mass transport from the North Pacific. In panels d-o, the thin light-colored curves represent annual mean values, and the thick dark-colored curves represent 10-year moving average values. The gray bars indicate the last 50 years of the 19 ka control simulation. The orange dashed lines indicate the first appearance of the dye tracer in the North Atlantic.

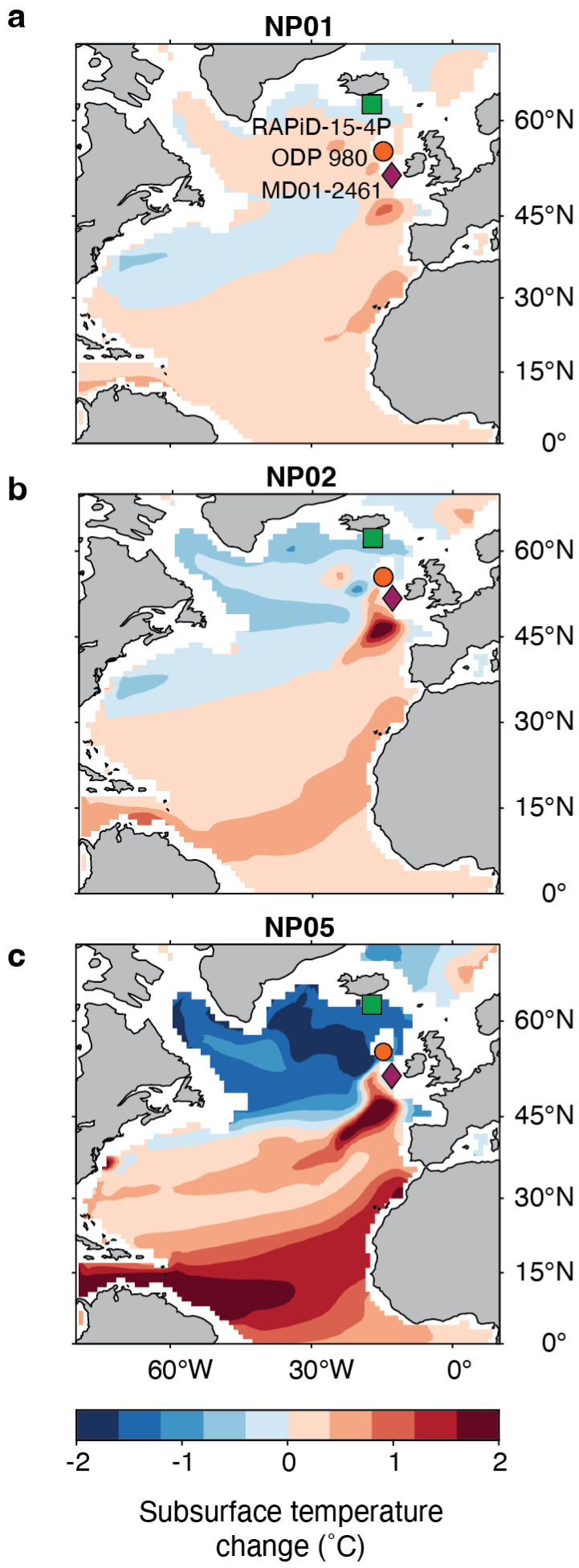

Figure 4 | a–c) Changes in annual mean North Atlantic subsurface temperature in a) NP01, b) NP02, and c) NP05. Mean values of 300–700 m depth are shown.

- DiNezio, P. N., Shanahan, T. M., Sun, T., Sun, C., Wu, X., Lawman, A., Lea, D., Kageyama, M., Merkel, U., Prange, M., Otto-Bliesner, B., & Zhang, X. (2025, 2025/08/01). Tropical response to ocean circulation slowdown raises future drought risk. *Nature, 644*(8077), 676-683.
- Lawman, A. E., Sun, C., Wu, X., Sun, T., Piatrunia, N., Gomez, K., Kageyama, M., Merkel, U., Prange, M., Otto-Bliesner, B., Zhang, X., DiNezio, P., & Shanahan, T. (2025, 2025/12/01/). Mechanisms driving the global tropical response to a weakened AMOC during Heinrich Stadial 1. *Quaternary Science Reviews, 369*, 109567.
- Pöppelmeier, F., Jeltsch-Thömmes, A., Lippold, J., Joos, F., & Stocker, T. F. (2023). Multi-proxy constraints on Atlantic circulation dynamics since the last ice age. *Nature Geoscience, 16*(4), 349-356.
- Seguinot, J., Rogozhina, I., Stroeven, A. P., Margold, M., & Kleman, J. (2016). Numerical simulations of the Cordilleran ice sheet through the last glacial cycle. *The Cryosphere, 10*(2), 639-664.
- Valdes, P. (2011). Built for stability. *Nature Geoscience, 4*(7), 414-416.
- Weijer, W., Cheng, W., Drijfhout, S. S., Fedorov, A. V., Hu, A., Jackson, L. C., Liu, W., McDonagh, E. L., Mecking, J. V., & Zhang, J. (2019). Stability of the Atlantic Meridional Overturning Circulation: A review and synthesis. *Journal of Geophysical Research: Oceans, 124*(8), 5336-5375.

- Zhou, Y., & McManus, J. F. (2024). Heinrich event ice discharge and the fate of the Atlantic Meridional Overturning Circulation. *Science, 384*(6699), 983-986.

2. Indeed, the exact freshwater hosing value and the associated ocean responses are likely model-dependent. Therefore, the sensitivity of AMOC responses to freshwater perturbations – such as those introduced directly over the middle of the North Atlantic (e.g., the IRD belt) – should be presented as a reference and discussed in greater detail.

We thank Reviewer #1 for this suggestion. Hosing simulations in which freshwater is added to the North Atlantic using this particular model, iCESM1.3 or its close predecessor, CESM1.2, has previously been heavily tested. For example, He et al. (2020) looked specifically at the North Atlantic subsurface response to AMOC weakening and found that a threshold of ~0.2 Sv of freshwater is required for the model to reproduce the subsurface warming response inferred from proxy data. Similarly, a series of studies focusing on hydroclimate responses to AMOC weakening show that a freshwater forcing of ~0.2 Sv is needed to drive the climate responses consistent with proxy data (DiNezio et al., 2025; Lawman et al., 2025). When only a smaller forcing of 0.1 Sv is applied, the model fails to reproduce the hydroclimate patterns outside of the Atlantic Ocean basin. In contrast, when a stronger freshwater forcing of 0.2 to 1 Sv is applied, the simulations are ranked among the highest in the multi-model comparisons against proxy data. In comparison, a recent estimation of meltwater release from the Laurentide Ice Sheet during Heinrich 1 is one order of magnitude smaller at ~0.02 Sv (Zhou & McManus, 2024), suggesting that while the model is capable of simulating the large-scale oceanic and atmospheric response to AMOC weakening, the model is overly stable to freshwater forcing, requiring a larger than actual freshwater input to produce the correct response. This over-stability of AMOC is a known issue for this model as well as many others. Please also see our response to the first comment about model over-stability.

Please see our revised Methods in line 357-390.

3. In the results and discussion section, the authors state that their findings "underscore the sensitivity of freshwater hosing experiments to the Atlantic Ocean's initial conditions." However, this needs to be presented and discussed in greater detail. In their model setup, they describe only one experiment BIIS01, which was continued from the experiment NP02. To support their conclusions, a more thorough analysis incorporating experiments initialized with different ocean conditions and varying hosing values should be conducted.
We thank Reviewer 1 for this suggestion. In this revision, we report 4 hosing simulations to test the impact of BIIS meltwater:

- **NP02_BIIS01:** previously named BIIS01 in our initial submission, where 0.1 Sv of freshwater forcing is added to the Irish west coast, branched from the climate of year 400 of NP02

- **ctrl_BIIS01:** new simulation branched from the climate of iTRACE 19ka, with 0.1 Sv of freshwater forcing added to the Irish west coast.
- **NP02_BIIS005:** new simulation branched from the climate of year 400 of NP02, but with only 0.05 Sv added to the Irish west coast.
- **ctrl_BIIS005:** new simulation branched from the climate of iTRACE 19ka, but with only 0.05 Sv of freshwater forcing added to the Irish west coast.

The comparison between NP02_BIIS01 and NP02_BIIS005 is used to understand the impact of BIIS meltwater events. In both NP02_BIIS005 and NP02_BIIS01 simulations, the AMOC weakens rapidly within the first 50 years (Fig. S12; copied below). However, in contrast to the continued weakening in NP02_BIIS01, the AMOC in NP02_BIIS005 begins to gradually strengthen after year 50. This is because the water masses entering the Atlantic basin through the Agulhas Leakage become saltier as the remaining North Pacific freshwater becomes exhausted, which outcompetes the weak freshwater forcing applied in the Northeast Atlantic in this experiment (i.e., NP02_BIIS005). This comparison suggests that the 0.05 Sv forcing scenario is likely too weak, while NP02_BIIS01 is the more realistic scenario for Heinrich Stadial 1. The total added BIIS freshwater is 0.94 and 1.88 m s.l.e. in NP02_BIIS005 and NP02_BIIS01, respectively, within the estimated freshwater contribution of 2 m s.l.e. based on ice sheet modeling (Hubbard et al., 2009) and thus represent a conservative forcing, considering the model's over-stability bias discussed above.

- Hubbard, A., Bradwell, T., Golledge, N., Hall, A., Patton, H., Sugden, D., Cooper, R., & Stoker, M. (2009). Dynamic cycles, ice streams and their impact on the extent, chronology and deglaciation of the British–Irish ice sheet. Quaternary Science Reviews, 28(7-8), 758-776.

Please see our revised main text in line 206-230 and Methods in line 393-411

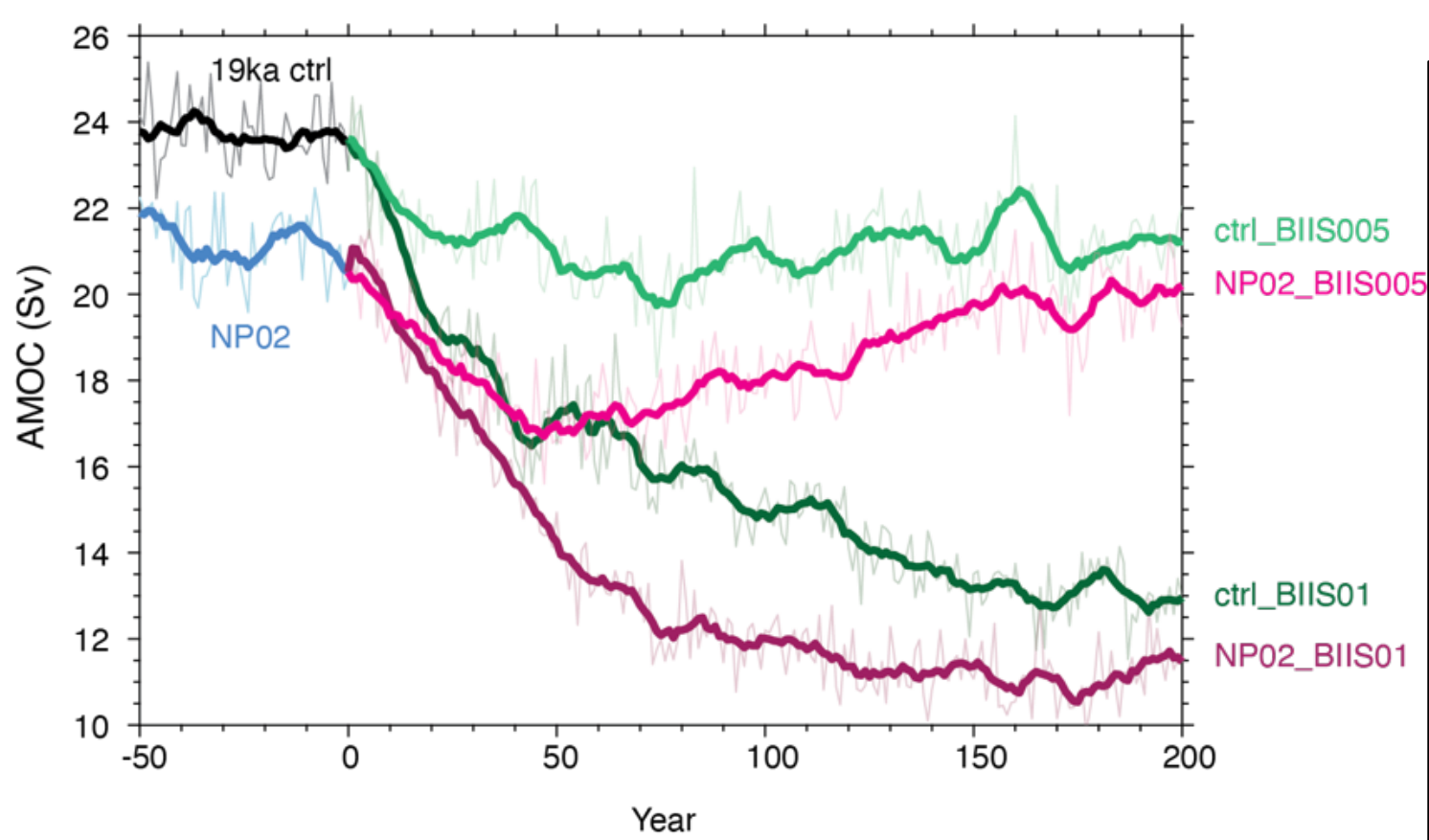

Fig. S12 | Comparison of AMOC change in BIIS experiments branched from the 19ka control (ctrl_BIIS01 and ctrl_BIIS005; red curves) vs. those from the NP02 (NP02_BIIS01 and NP02_BIIS005; green curves) boundary conditions. The simulations branched from NP02 consistently show a weaker AMOC compared to their counterparts branched from the 19ka control run.

The comparisons between BIIS simulations run with NP02 vs 19ka conditions (i.e., NP02_BIIS01 vs ctrl_BIIS01 and NP02_BIIS005 vs ctrl_BIIS005) shows that the AMOC is consistently weaker when the North Atlantic has already been freshened after a Siku event (Fig. S12), suggesting an important role of pre-existing Siku meltwater in enhancing AMOC weakening. Furthermore, NP02_BIIS01 and ctrl_BIIS01 show drastically different northern North Atlantic subsurface temperature responses: NP02_BIIS01 produces subsurface warming in the northern North Atlantic after year ~50, whereas ctrl_BIIS01 remains anomalously cooler for an extended period until year 100 (Fig. S13 copied below),

suggesting that the preconditioning of the North Atlantic by S1 meltwater could drastically accelerate the basal melting of the Laurentide ice shelf. The different subsurface temperature responses to AMOC weakening stem from the competing impacts of warming associated with suppressed vertical mixing and cooling due to reduced poleward oceanic heat transport (He et al., 2020). In NP02_BIIS01, Siku meltwater has preconditioned the North Atlantic with a weaker initial AMOC state and more buoyant surface waters, which, combined with BIIS meltwater, makes it easier to cross the threshold where subsurface warming would prevail. Subsequently, this subsurface warming would drive iceberg discharge from the Laurentide Ice Sheet, leading to H1. As such, the role of S1 meltwater in triggering H1 may involve both initiating BIIS meltwater release via a weakened AMOC and its lingering freshwater effect that amplifies the BIIS precursor event.

Please see our revised main text in line 232-254 and Methods in line 413-420.

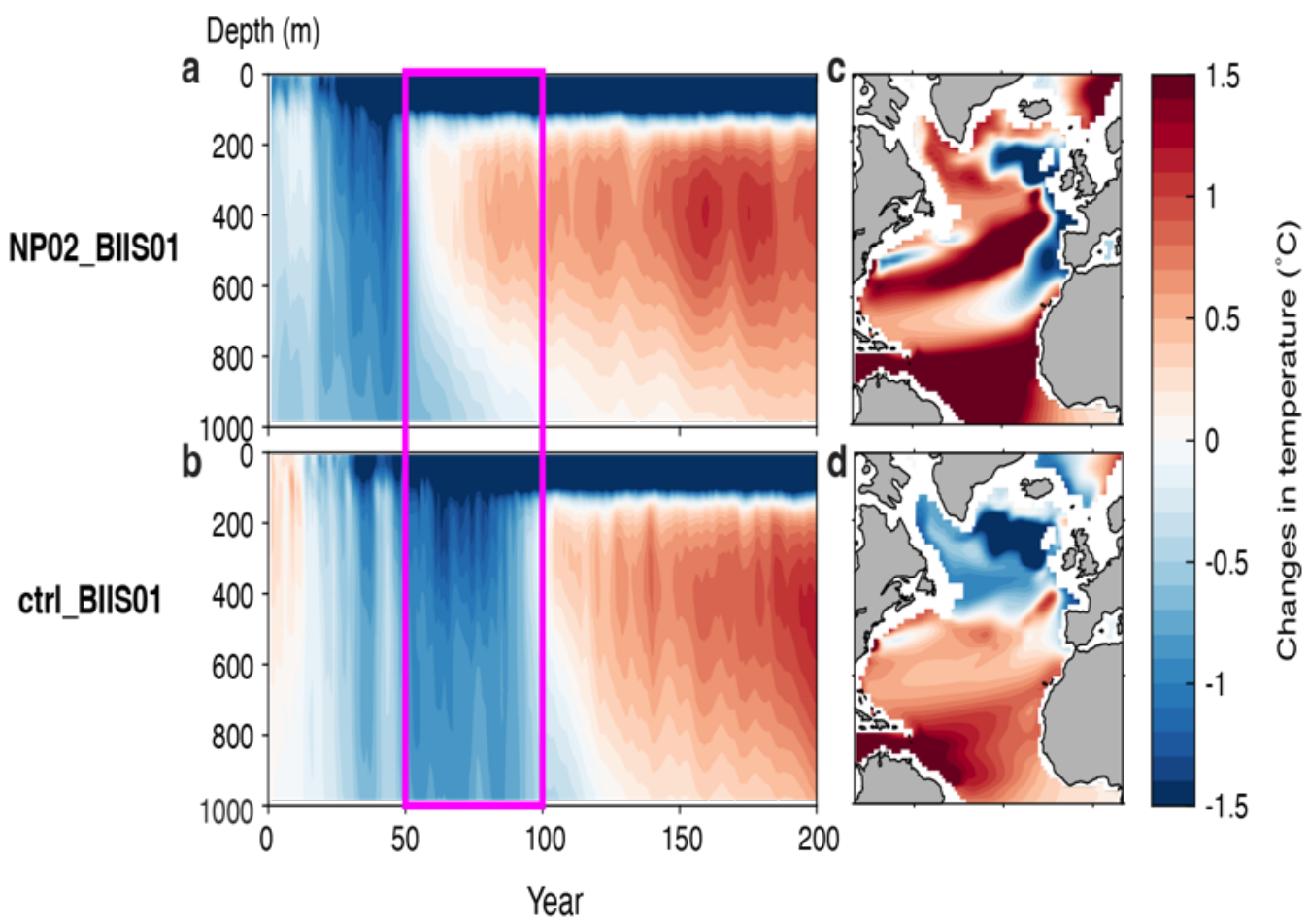

Fig. S13. Comparison of 0.1Sv of meltwater flux from the BIIS under the boundary conditions of NP02 and 19ka control. a-b) Hovmoller plots of northern North Atlantic (north of 45°N, including the Labrador Sea but not the Greenland-Iceland-Norwegian Seas) temperature change in depth and time in a) NP02_BIIS01 and b) ctrl_BIIS01, respectively. **c-d)** North Atlantic subsurface temperature change in c) NP02_BIIS01 and d) ctrl_BIIS01 average over year 50-100 of the simulations. The magenta box on the left panels highlights the difference between the two simulations between year 50 and 100.

4. The model only discusses the influences of the ocean. However, since the manuscript aims to link the Siku events with global climate effects and subsequent Heinrich events, it is also essential to address the atmospheric responses, the resulting surface mass balance changes on the ice sheets, and potential impacts on ice sheet dynamics (e.g. Heinrich events). As part of the Earth system, I believe that atmospheric responses are also crucial to the land-based northern hemisphere ice sheets.

We thank Reviewer 1 for this suggestion. In this manuscript, we use a positive degree day (PDD) model to show that the atmospheric response to North Pacific freshwater forcing, primarily due to surface cooling, would also likely promote expansion, rather than retreat, of the Laurentide Ice Sheet over the Labrador Sea (Fig. S10). Together with a northern North Atlantic subsurface cooling in these experiments, our results reject the hypothesis that S1 meltwater could directly trigger Laurentide ice shelf melting by itself.

Meanwhile, we also note that over the BIIS, the PDD surface mass balance model shows a net positive in all three NP hosing simulations (Fig. S10) due to cooling and increased snowfall, which would promote seaward advances of the BIIS. Meanwhile, the subsurface warming off the Irish west coast could destabilize the BIIS from its marine grounding line.

The combination of surface growth and basal melt provides a mechanism for enhanced iceberg calving that could explain the observed increase in BIIS-sourced IRD during S1

Please see our revised main text in line 181-185, 194-200 and Methods in line 422-441

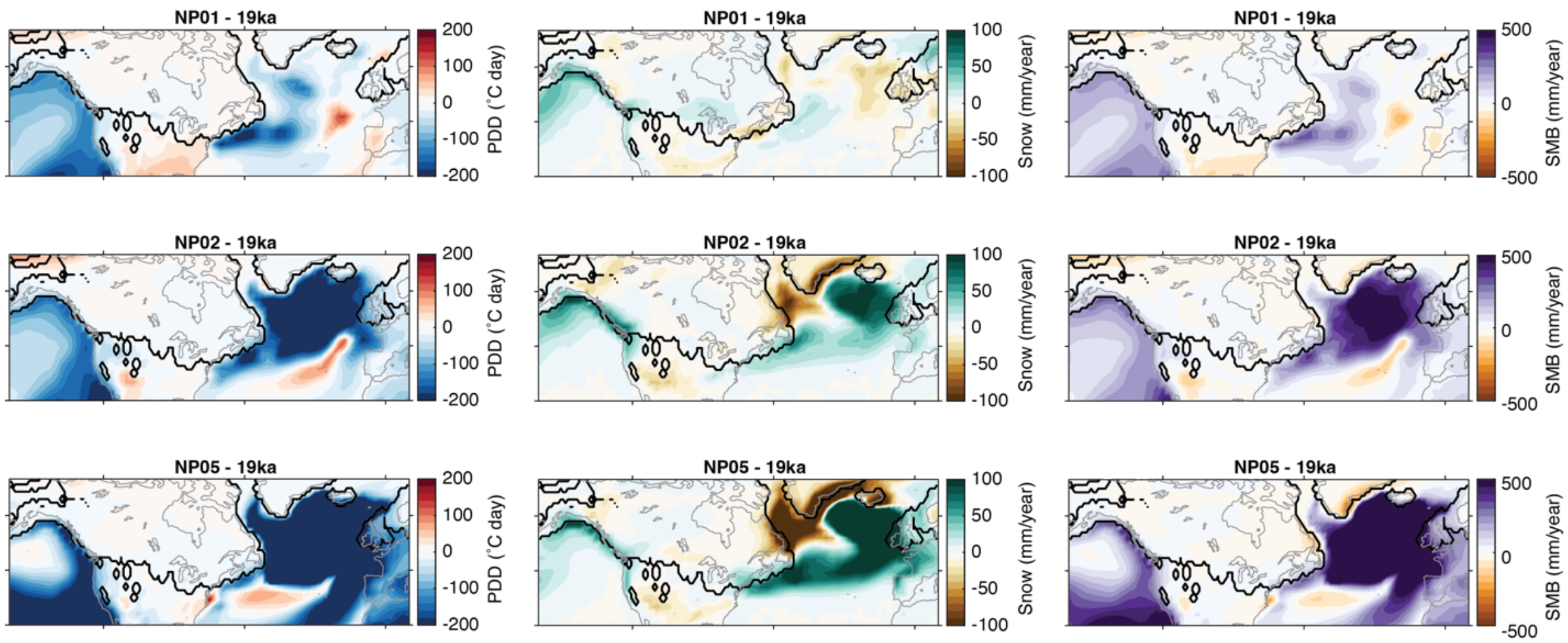

Fig. S10 | Estimated impacts of North Pacific meltwater on ice sheet surface mass balance. Left: changes in mean annual positive degree days in NP01, NP02, and NP05 compared to the 19ka control. Middle: changes in mean annual snowfall in NP01, NP02, and NP05 compared to the 19ka control. Right: changes in mean annual surface mass balance in NP01, NP02, and NP05 compared to the 19ka control. Thick black lines mark the spatial extent of ice sheets.

5. This study is based on one mechanism for triggering Heinrich events, with ocean subsurface warming identified as the key driver. However, various other mechanisms have been proposed, such as the binge-purge mechanism. Additionally, a recent study (Schannwell et al., 2024) proposes that multiple DO events could lead to phasing locking with Heinrich events. It would be necessary to include more discussions of these alternative theories.

We thank Reviewer #1 for this suggestion. In this revision, we review of existing mechanisms for triggering Heinrich events in the introduction, citing studies by MacAyeal (1993), Schannwell et al. (2024), Marcott et al. (2011), Max et al. (2022), Alvarez-Solas et al. (2010), and Bassis et al. (2017). Please see revised text in line 56-79.

Specific comments:

Line 74-79: Why specifically 0.2 and 0.5 Sv? As is mentioned earlier, Seguinot et al. (2016) estimates a sea level equivalent of approximately 8 meters for the Cordilleran Ice Sheet during the LGM. Based on this, I would expect a smaller freshwater input associated with Siku events. Did you also test the response to, for example, 0.1 Sv?

We thank Reviewer #1 for the specific comment on this issue. Following reviewer #1's suggestion, we performed a 0.1 Sv North Pacific hosing simulations. Please see our detailed response to major comment #1 above.

Line 115-127: This paragraph, along with the associated discussion on present-day water mass transportation, appears out of place. It lacks a smooth transition and does not align with the logical flow of the current section. I recommend rephrasing it for better integration or moving it to the discussion section, where it may be more relevant.
We agree with the reviewer's comment that this paragraph appears out of place. We have rewritten this paragraph and separated the model and proxy data into two paragraphs to improve the transition smoothness for an improved logical flow. Please see revised text in line 124-135.

Line 129-135, 185-189. In this section, the author propose that an initial AMOC weakening could be largely amplified by the sequential freshwater discharges from the BIIS or LIS. Therefore, it is essential to compare AMOC responses without the initial AMOC weakening. More specifically, add a reference simulation analogous to BIIS01, but initialized from the 19 ka control rather than NP02, would provide a more robust comparison.
We agree with the reviewer that it is important to compare AMOC responses to BIIS discharge without the initial AMOC weakening due to North Pacific freshwater. We ran a new 0.1Sv BIIS hosing simulation initialized from the 19 ka control ("ctrl_BIIS01) to compare with our "NP02_BIIS01" run. Please see our detailed response to major comment #3 above.

Line 151-152, 157-159: I question to what extent the retreat of the BIIS is driven by subsurface warming resulting from the initial AMOC weakening, since the ocean interface of the BIIS is rather small. While the authors estimate a 2.5 m global sea-level change, I am skeptical about how much of this contribution specifically originates from the BIIS. Clarification or additional evidence to support this attribution would strengthen the argument.

We thank reviewer for this comment. We acknowledge that the BIIS retreat driven directly by subsurface warming may be limited due to a relatively small ocean interface. In this revision, we argue that both the surface cooling and subsurface warming in response to the initial AMOC weakening contributed to increased calving activity at the Irish west coast. Surface cooling with increased snowfall leads to a positive surface mass balance, while subsurface warming could destabilize the BIIS from its marine grounding line. Furthermore, we argue that debuttressing of the BIIS from its marine grounding could have destabilized the ice sheet and triggered accelerated retreat. This hypothesis is consistent with sedimentary records showing increased meltwater discharge from the southern European Ice Sheets to the Northeast Atlantic via the Channel River, as well as paleoglaciological evidence for a rapid retreat of the BIIS near 19 ka. A transient ice sheet model predicts that the BIIS alone discharged ~2 m sea-level equivalent of meltwater between 19ka and 17ka

(Hubbard et al., 2009). This process could have been accelerated via the destabilization effect of the initial AMOC weakening, which is not included in that transient modeling study.

Please see the revised text in line 192-204

- Hubbard, A., Bradwell, T., Golledge, N., Hall, A., Patton, H., Sugden, D., Cooper, R., & Stoker, M. (2009). Dynamic cycles, ice streams and their impact on the extent, chronology and deglaciation of the British–Irish ice sheet. Quaternary Science Reviews, 28(7-8), 758-776.

Line 164-165: Following the previous comment, conducting additional hosing experiments with varying freshwater fluxes would be essential to evaluate the sensitivity and responses of the AMOC more comprehensively. This would provide a clearer understanding of the potential range of impacts caused by different magnitudes of freshwater input.

We thank the reviewer for this specific comment expanding on major comment #3. We addressed this concern by running additional hosing simulations with varying freshwater fluxes and initial conditions to understand the impact of BIIS meltwater. Please see our response to major comment #3 above.

Line 209-210: The authors briefly mention atmospheric (or previously climate) responses; however, there is no detailed discussion on this aspect. Expanding on the atmospheric responses, and impacts on the surface mass balance changes are essential, as the Northern Hemisphere ice sheets are largely influenced by atmospheric processes.

We thank the reviewer for this specific comment expanding on major comment #4. We addressed this concern using a positive degree day surface mass balance model. Please see our response to major comment #4 above.

Fig. S9: Since this is a key part of the main conclusion of the manuscript, it would be better to move this figure into the main text for better integration and emphasis.

Thanks for this suggestion. We have moved the figure to the main text as Fig 5.

We thank Reviewer #1 once again for the thoughtful comments and suggestions, which have greatly strengthened our manuscript. We hope that our revisions satisfactorily address all the concerns.

Response to Reviewer #2

Reviewer #2 (Remarks to the Author):

Review Nat Comm: Chijun Sun et al. (2025).

Heinrich events are cold periods during the (last) deglaciation associated with iceberg meltwater release from the Laurentide ice sheet, observed in the North Atlantic. Siku events are observed in the North Pacific and are associated with iceberg melting from the Cordilleran ice sheet. This study investigates the influence of Siku events on the occurrence of Heinrich events. It is an interesting study because the influence of freshwater feedbacks on the climate system and the ocean in specific are often omitted. In the ideal world, these freshwater fluxes come from dynamic ice sheet models in order to have realistic numbers.

This study explains the occurrence of Heinrich events as a chain reaction where a Siku event in the North Pacific leads to ice shelf melting of the British Irish Ice Sheet (BIIS), the resulting freshwater from the BISS would further weaken the AMOC and lead to subsurface warming in the subpolar North Atlantic. I find it an interesting study with a bold conclusion based on one extended experiment. I believe these findings could be better supported if some additional simulations would be performed, which I further detail below.

We thank Reviewer #2 for the supportive feedback and helpful comments. We have carefully addressed all the concerns. Please find our responses to each comment below.

The simulations are 400 years long. One additional experiments is 600 years long, where an additional freshwater flux from the BIIS is added at model year 400 when the freshwater flux in the North Pacific is halted. It would be interesting to have a control run where no additional freshwater is added during the entire length of the simulations (600 years) and another run where the freshwater flux in the North Pacific is halted at year 400 and no additional freshwater flux is added from the BIIS to see whether the AMOC recovers. Also, only one additional run is performed as a continuation from NP02. It would be interesting whether the simulations show the same results for other meltwater releases (NP05 or another additional freshwater release experiment).

We thank Reviewer #2 for the constructive feedback. We agree that the additional simulations proposed by Reviewer #2 will be interesting. Here in this revision, we performed a 200-year extension of the control run (ctrl_ext) to show that the climate and AMOC is stable in the control run. Please see Main text line 349-355.

We also run 3 additional BIIS freshwater sensitivity tests (NP02_BIIS01, NP02_BIIS005, ctrl_BIIS01, ctrl_BIIS005) to test the impacts of forcing strengths and the initial conditions (branched from NP02 vs 19ka). Please see Main text line 392-420.

While we did not run an additional run where the freshwater flux in the North Pacific is halted at year 400 and no additional freshwater flux is added from the BIIS to see whether the AMOC recovers. We can gain insights from the new weakly forced BIIS experiment, NP02_BIIS005. This experiment shows a rapid AMOC weakening in the first 50 years in response to the 0.05 Sv freshwater forcing added to the Irish west coast. However, the AMOC begins to gradually strengthen after year 50 (Fig. S12). This is because when the freshwater flux in the North Pacific is halted, the water masses entering the Atlantic basin through the Agulhas Leakage become saltier as the remaining North Pacific freshwater becomes exhausted, which outcompetes the weak freshwater forcing applied in the Northeast Atlantic in this experiment. The 50-year duration is also consistent with the timing that we noted for water mass transport from the North Pacific to the North Atlantic NADW formation sites. Please see our revised text in line 402-409.

Also see Table S1 copied below for a list of all simulations included in this manuscript.

Table S1. List of simulations reported in this study

ID	Hosing region	Boundary conditions	Freshwater flux	Total length
Control	N/A	N/A	N/A	N/A
ctrl_ext	N/A	iTRACE 19 ka	N/A	200 years
NP01	45–70°N, 160–235°E	iTRACE 19 ka	0.1 Sv	400 years
NP02	45–70°N, 160–235°E	iTRACE 19 ka	0.2 Sv	400 years
NP05	45–70°N, 160–235°E	iTRACE 19 ka	0.5 Sv	400 years
NP02_BIIS01	0–15°W, 50–60°N	NP02 Year 400	0.1 Sv	200 years
NP02_BIIS005	0–15°W, 50–60°N	NP02 Year 400	0.05 Sv	200 years
ctrl_BIIS01	0–15°W, 50–60°N	iTRACE 19 ka	0.1 Sv	200 years
ctrl_BIIS005	0–15°W, 50–60°N	iTRACE 19 ka	0.05 Sv	200 years
NA02	80˚W–20˚E, 50–70˚N	iTRACE 19 ka	0.2 Sv	200 years

Line by line comments:

L74-75: How did you choose the values of 0.2 and 0.5 Sv and why are these numbers exactly chosen?
We thank Reviewer #2 for this valid question. In this revision, we performed an additional North Pacific hosing simulation with 0.1 Sv applied to the North Pacific. The added freshwater in these three sensitivity tests (NP01, NP02, NP05) is equivalent to 3.8, 7.5, and 18.8 meter sea-level equivalent, capturing the possible range of freshwater fluxes associated with Cordilleran ice sheet retreat and allowing us to assess the robustness and

sensitivity of AMOC's response to the North Pacific meltwater event. We clarify this goal in main text line 97-98 and in Methods in line 359-362.

L78-79: It is indeed true that a sea-level rise of 18.8 m in 400 years is a realistic number during the peak of the deglaciation. However, this number comes mainly from 4 main ice masses (North American ice sheet, Fennoscandian ice sheet, Greenland ice sheet and the Antarctic ice sheet). It is estimated that the North American ice sheet (Cordilleran ice sheet and Laurentide ice sheet combined) had a volume equivalent to ~60-80m sea level, or ~45-60% of the 130 m sea-level lowering at the Last Glacial Maximum. It is thought that the Cordilleran ice sheet disappeared fast – in about 4000 years. Given that the Cordilleran ice sheet is the smaller one, a sea-level rise equivalent up to 18.8 m in 400 years is high. Do you assume that the Laurentide ice sheet also partly drained into the North Pacific?

We thank reviewer #2 for this concern. We do not assume that the Laurentide Ice Sheet also partly drained into the North Pacific. We acknowledge that the imposed meltwater forcing in NP02 (7.5 m) and especially NP05 (18.8 m) may have overestimated the actual meltwater contribution from the Cordilleran Ice Sheet during S1, given that the total Cordilleran ice sheet volume is estimated to be ~8.8 m s.l.e. (Seguinot et al., 2016) and that melt from other ice sheets also contributed to the global sea-level change at 19 ka.

However, we would like to argue that such exaggerations can be justified because the current generation of general circulation models is known to produce an overly stable AMOC (Valdes, 2011). For example, while H1 meltwater release from the Laurentide Ice Sheet has been estimated at only ~0.02 Sv (Zhou & McManus, 2024), substantially stronger forcings (0.25 to 1 Sv) are typically required to achieve atmospheric and oceanic responses that are consistent with proxy data (DiNezio et al., 2025; Lawman et al., 2025; Pöppelmeier et al., 2023). The models' over-stability may result from model biases in surface water salinity in the South Atlantic, which cause the models to have a net import of freshwater in the South Atlantic, contradicting the net export shown in observational data (Weijer et al., 2019). This bias salinifies the Atlantic Ocean when the AMOC is weakened, internally reinvigorating the AMOC when the external forcing is too small (Weijer et al., 2019). In this context, our slight exaggeration of freshwater forcing is not only justified, but also allows us to test the sensitivity and robustness of the AMOC's response to varying forcing intensities.

Furthermore, in this revision, we performed an additional experiment, with 0.1 Sv of freshwater added to the North Pacific (3.8 m sea level equivalent). Our three sensitivity experiments, NP01, NP02, and NP05, show qualitatively similar responses, including freshwater transport pathway (Fig. 2; copied below) and North Atlantic subsurface temperature change (Fig. 4a-c; copied below). This suggests that the mechanisms proposed in our study are robust and insensitive to hosing strength.

Please see our revised Methods section in line 357-390.

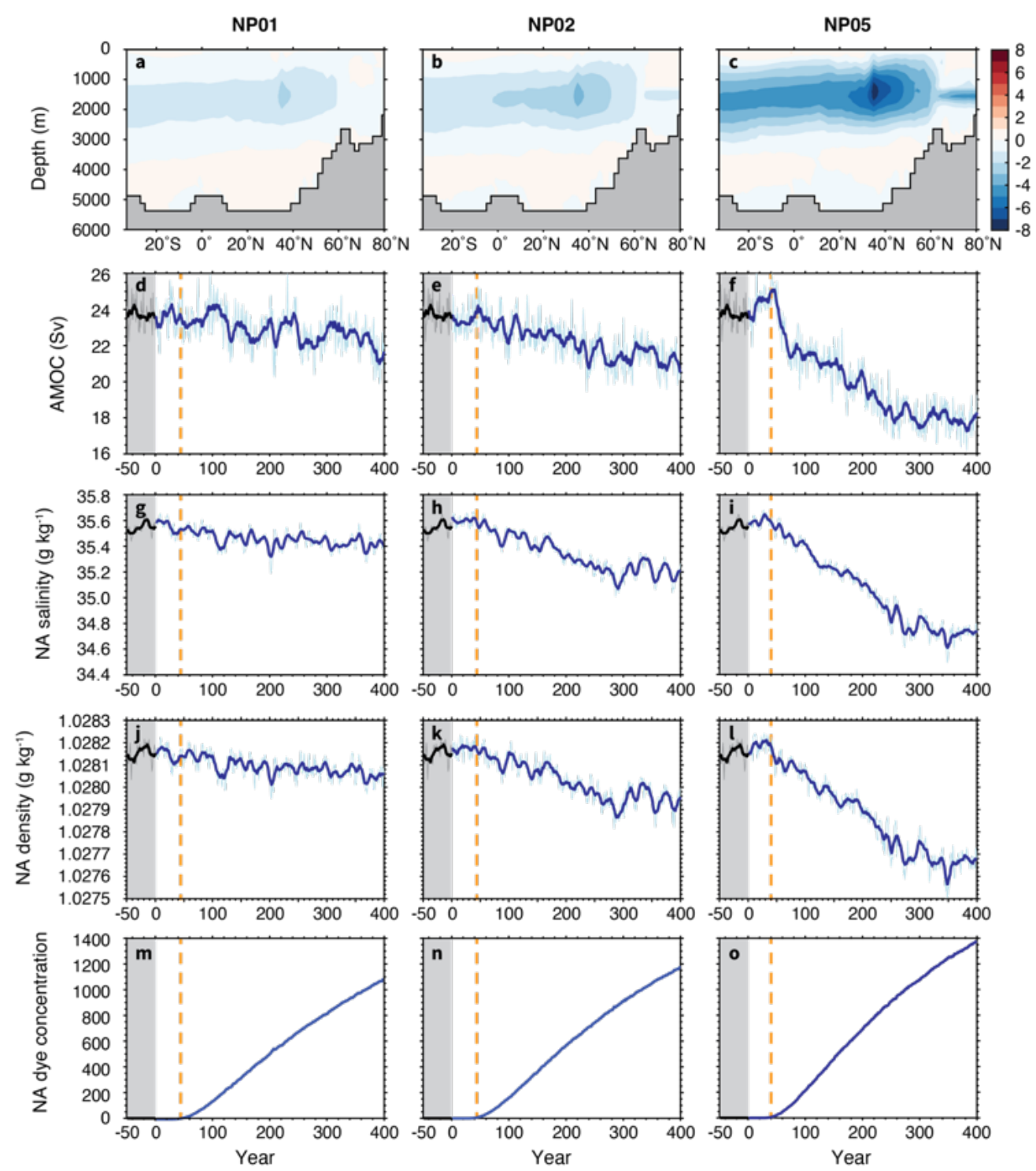

Figure 2 | Relationship between AMOC weakening and freshwater transport from the North Pacific. a-c) Changes in AMOC strength. **d-f)** Evolution of AMOC strength (defined as the maximum stream function between 10 and 60 ° N). **g-i)** Evolution of upper water (top 100 m) salinity in the North Atlantic (45-60°N). **j-l)** Evolution of upper water density in the North Atlantic. **m-o)** Dye tracer concentration in the North Atlantic upper water, indicative of water mass transport from the North Pacific. In panels d-o, the thin light-colored curves represent annual mean values, and the thick dark-colored curves represent 10-year moving average values. The gray bars indicate the last 50 years of the 19 ka control simulation. The orange dashed lines indicate the first appearance of the dye tracer in the North Atlantic.

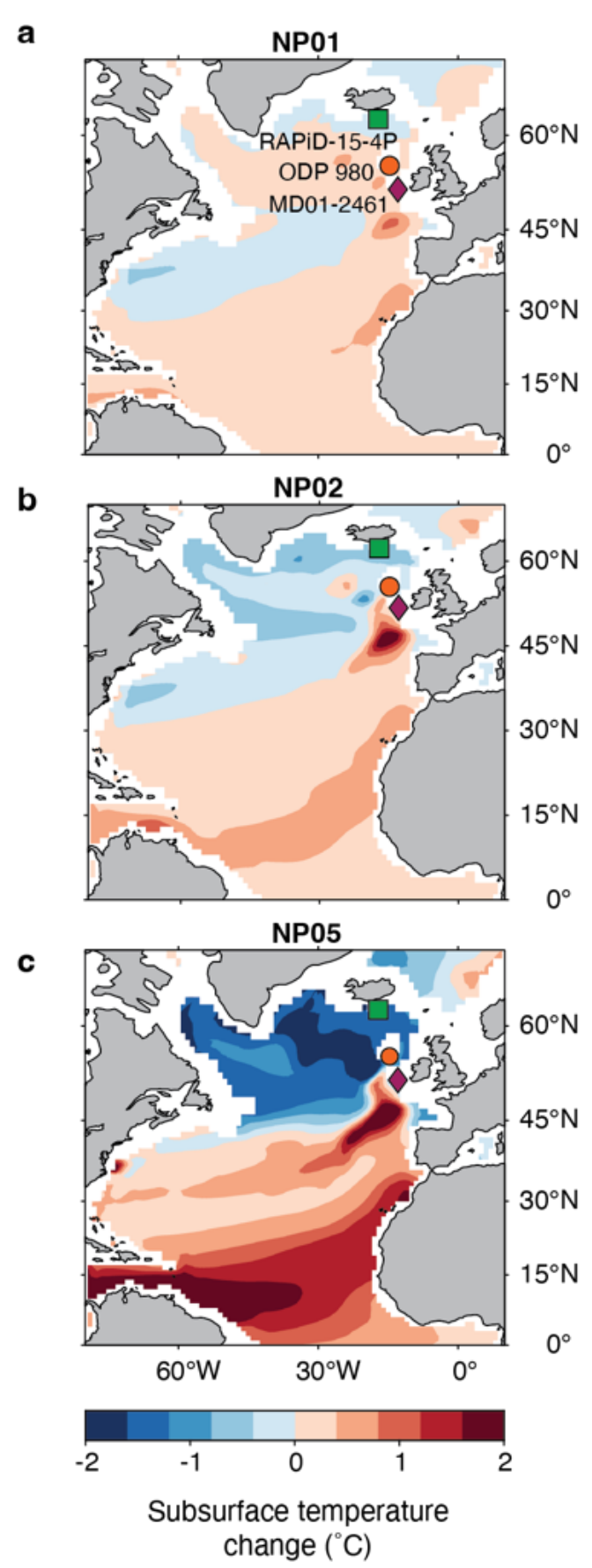

Figure 4 | a–c) Changes in annual mean North Atlantic subsurface temperature in a) NP01, b) NP02, and c) NP05. Mean values of 300–700 m depth are shown.

- DiNezio, P. N., Shanahan, T. M., Sun, T., Sun, C., Wu, X., Lawman, A., Lea, D., Kageyama, M., Merkel, U., Prange, M., Otto-Bliesner, B., & Zhang, X. (2025, 2025/08/01). Tropical response to ocean circulation slowdown raises future drought risk. *Nature, 644*(8077), 676-683.
- Lawman, A. E., Sun, C., Wu, X., Sun, T., Piatrunia, N., Gomez, K., Kageyama, M., Merkel, U., Prange, M., Otto-Bliesner, B., Zhang, X., DiNezio, P., & Shanahan, T. (2025, 2025/12/01/). Mechanisms driving the global tropical response to a weakened AMOC during Heinrich Stadial 1. *Quaternary Science Reviews, 369*, 109567.
- Pöppelmeier, F., Jeltsch-Thömmes, A., Lippold, J., Joos, F., & Stocker, T. F. (2023). Multi-proxy constraints on Atlantic circulation dynamics since the last ice age. *Nature Geoscience, 16*(4), 349-356.
- Seguinot, J., Rogozhina, I., Stroeven, A. P., Margold, M., & Kleman, J. (2016). Numerical simulations of the Cordilleran ice sheet through the last glacial cycle. *The Cryosphere, 10*(2), 639-664.
- Valdes, P. (2011). Built for stability. *Nature Geoscience, 4*(7), 414-416.
- Weijer, W., Cheng, W., Drijfhout, S. S., Fedorov, A. V., Hu, A., Jackson, L. C., Liu, W., McDonagh, E. L., Mecking, J. V., & Zhang, J. (2019). Stability of the Atlantic Meridional Overturning Circulation: A review and synthesis. *Journal of Geophysical Research: Oceans, 124*(8), 5336-5375.
- Zhou, Y., & McManus, J. F. (2024). Heinrich event ice discharge and the fate of the Atlantic Meridional Overturning Circulation. *Science, 384*(6699), 983-986.

L81-82: Say how you would investigate the causes of AMOC weakening in this study instead of giving a result here.

We thank Reviewer #2 for this suggestion. We have revised the result-giving sentence to the text below as the last sentences before transitioning into Results:

"In the first set of simulations, we apply a freshwater flux of 0.1, 0.2, or 0.5 Sverdrup (Sv; 1 Sv = 10^6 m^3 s^{-1}) to the Northeast Pacific (Fig. S1) for 400 years under the glacial boundary conditions (hereafter "NP01", "NP02", and "NP05") using the fully coupled Community Earth System Model version 1.3 [30,31] (see Methods). These varying forcing intensities allow us to assess the robustness and sensitivity of AMOC's response to the North Pacific meltwater event. Building upon these results, we further perform additional simulations to examine whether S1 meltwater, in conjunction with European Ice Sheet melt, could have triggered Heinrich Event 1 (H1; 15–17 ka; see Methods)."

Please see our revised text in line 93-100.

L99: Why does figure S6 appear in the text before you first mention S3, S4 or S5? Please order the figures chronological by first appearance in the text.

We thank Reviewer #2 for this careful examination. We have moved a revised Figure S6 to the main text as the new Figure 3.

L166: Why did you decide to run an additional experiments where freshwater is released from the BIIS only for the NP02 scenario and not for the NP05 scenario?

We thank Reviewer #2 for this valid question. NP02 represents the moderate scenario for S1 freshwater forcing, considering the model's over-stability issue discussed above. As shown in figure 4 (copied below), NP02 shows strong agreement with North Atlantic subsurface temperature records. The rationale is provided in line 396-398.

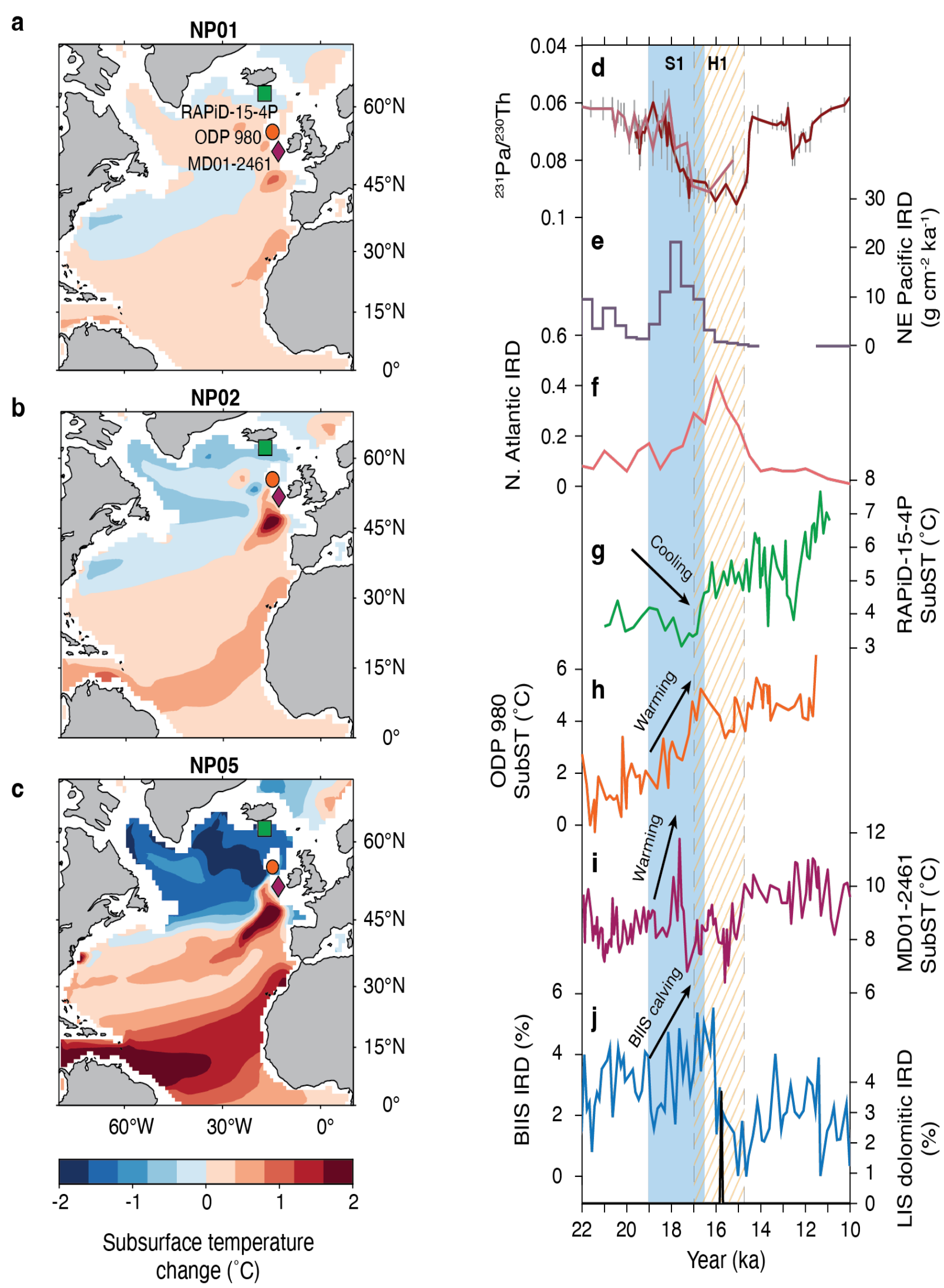

Figure 4 | Comparison of simulated North Atlantic response to proxy records of Northeast Atlantic subsurface temperature along with meltwater events and AMOC variability. a–c) Changes in annual mean North Atlantic subsurface temperature in a) NP01, b) NP02, and c) NP05. Mean values of 300–700 m depth are shown. Green square, orange circle, and purple diamond denote the locations of sites RAPiD-15-4P, ODP 980, and MD01-2461, where subsurface temperature reconstructions are available and shown on the right. **d)** $^{231}Pa/^{230}Th$-based AMOC reconstruction from the Bermuda Rise [6-8]. **e)** IRD record from U1419 in the Northeast Pacific [27]. **f)** North Atlantic IRD stack [66]. **g)** Subsurface temperature (SubST) record from RAPiD-15-4P [67]. **h)** SubST record from ODP 980 [68]. **i)** SubST from MD01-2461 [39]. Subsurface temperatures in g–i are based on Mg/Ca of subsurface dwelling foraminfera *N. Pachyderma* (sinistral). **j)** IRD record form MD01-2461 [39], with blue showing the percentage of BIIS-sourced grains and a single black spike marking Laurentide-derived dolomitic IRD. For data from MD01-2461, Max et al. [16]'s updated age model is used.

L181: Why did you not perform a run where you only have meltwater from the BIIS without precursor meltwater release from a Siku event? You have all the modelling tools in place not to make speculations and hypothesis whether the freshwater forcing could be sufficient.

We thank Reviewer #2 for this suggestion. In this revision, we report 4 hosing simulations to test the impact of BIIS meltwater:

- **NP02_BIIS01:** previously named BIIS01 in our initial submission, where 0.1 Sv of freshwater forcing is added to the Irish west coast, branched from the climate of year 400 of NP02
- **ctrl_BIIS01:** new simulation branched from the climate of iTRACE 19ka, with 0.1 Sv of freshwater forcing added to the Irish west coast.
- **NP02_BIIS005:** new simulation branched from the climate of year 400 of NP02, but with only 0.05 Sv added to the Irish west coast.
- **ctrl_BIIS005:** new simulation branched from the climate of iTRACE 19ka, but with only 0.05 Sv of freshwater forcing added to the Irish west coast.

The comparisons between BIIS simulations run with NP02 vs 19ka conditions (i.e., NP02_BIIS01 vs ctrl_BIIS01 and NP02_BIIS005 vs ctrl_BIIS005) shows that the AMOC is consistently weaker when the North Atlantic has already been freshened after a Siku event (Fig. S12 copied below), suggesting an important role of pre-existing Siku meltwater in enhancing AMOC weakening. Furthermore, NP02_BIIS01 and ctrl_BIIS01 show drastically different northern North Atlantic subsurface temperature responses: NP02_BIIS01 produces subsurface warming in the northern North Atlantic after year ~50, whereas ctrl_BIIS01 remains anomalously cooler for an extended period until year 100 (Fig. S13 copied below), suggesting that the preconditioning of the North Atlantic by S1 meltwater could drastically accelerate the basal melting of the Laurentide ice shelf. The different subsurface temperature responses to AMOC weakening stem from the competing impacts of warming associated with suppressed vertical mixing and cooling due to reduced poleward oceanic heat transport (He et al., 2020). In NP02_BIIS01, Siku meltwater has preconditioned the North Atlantic with a weaker initial AMOC state and more buoyant surface waters, which, combined with BIIS meltwater, makes it easier to cross the threshold where subsurface warming would prevail. Subsequently, this subsurface warming would drive iceberg discharge from the Laurentide Ice Sheet, leading to H1. As such, the role of S1 meltwater in triggering H1 may involve both initiating BIIS meltwater release via a weakened AMOC and its lingering freshwater effect that amplifies the BIIS precursor event.

Please see our revised main text in line 232-254 and Methods in line 413-420.

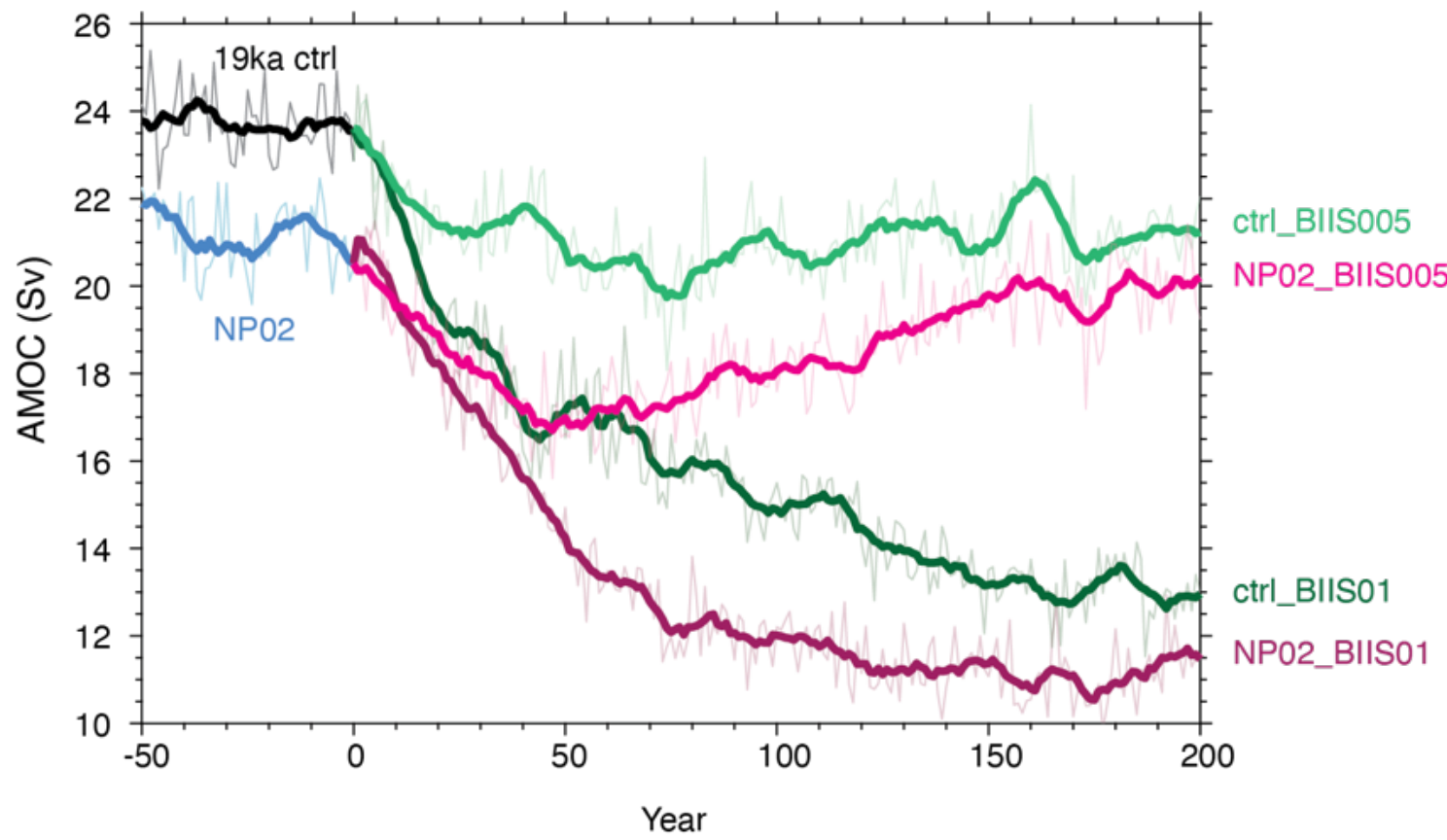

Fig. S12 | Comparison of AMOC change in BIIS experiments branched from the 19ka control (ctrl_BIIS01 and ctrl_BIIS005; red curves) vs. those from the NP02 (NP02_BIIS01 and NP02_BIIS005; green curves) boundary conditions. The simulations branched from NP02 consistently show a weaker AMOC compared to their counterparts branched from the 19ka control run.

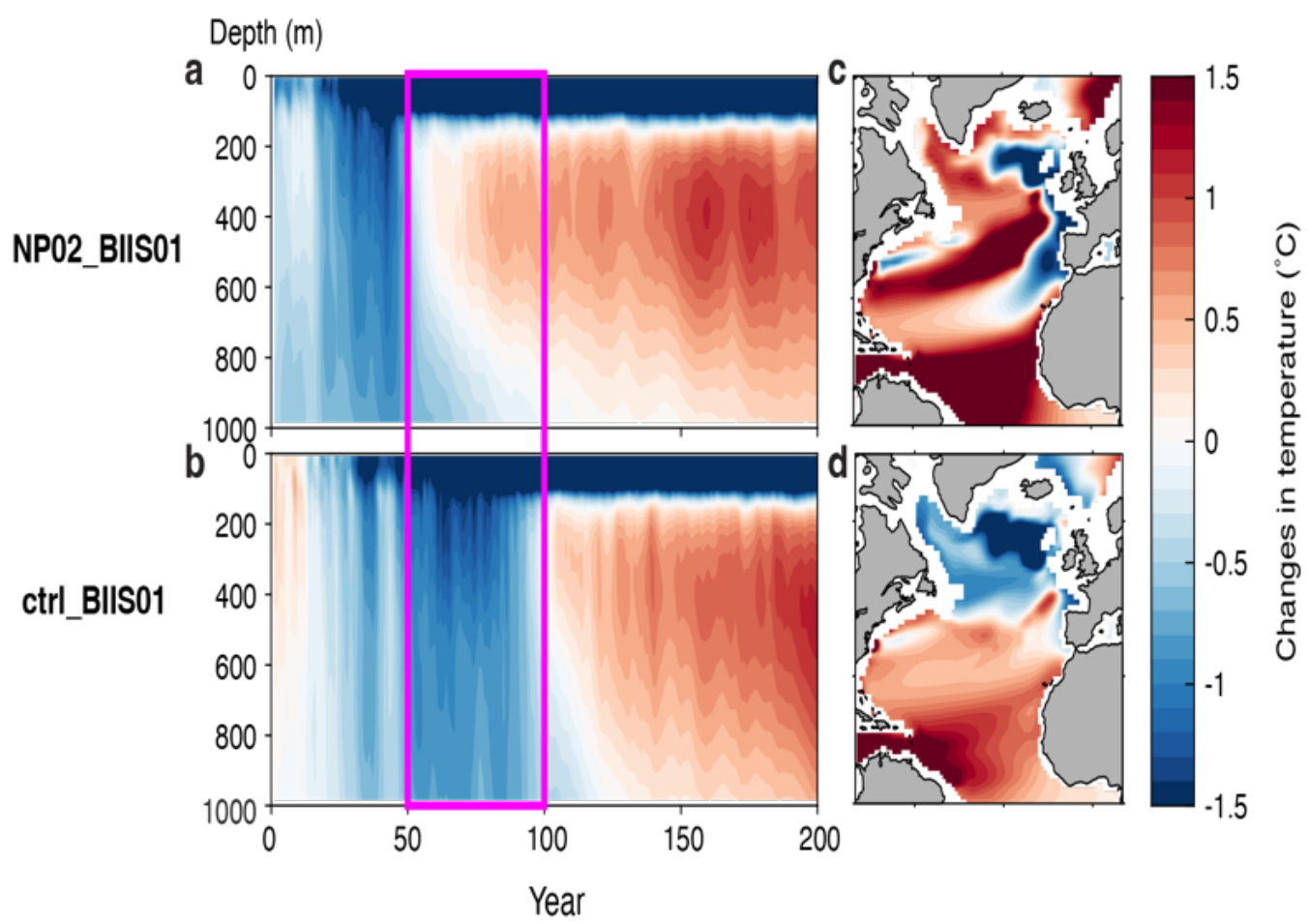

Fig. S13. Comparison of 0.1Sv of meltwater flux from the BIIS under the boundary conditions of NP02 and 19ka control. a-b) Hovmoller plots of northern North Atlantic (north of 45°N, including the Labrador Sea but not the Greenland-Iceland-Norwegian Seas) temperature change in depth and time in a) NP02_BIIS01 and b) ctrl_BIIS01, respectively. **c-d)** North Atlantic subsurface temperature change in c) NP02_BIIS01 and d) ctrl_BIIS01 average over year 50-100 of the simulations. The magenta box on the left panels highlights the difference between the two simulations between year 50 and 100.

We thank Reviewer #2 once again for the thoughtful comments and suggestions, which have greatly strengthened our manuscript. We hope that our revisions satisfactorily address all the concerns.

Response to Reviewer #3

Reviewer #3 (Remarks to the Author):

Review notes on 'Triggering of Heinrich Events by Cordilleran Ice Sheet Meltwater into the North Pacific' by Sun et al.

Sun et al., perform a series of freshwater hosing experiments to investigate possible connections between Northeast Pacific Siku events and North Atlantic Heinrich events using CESM1.3, which uses an ocean model with 1 degree horizontal resolution. Based on the results of their simulations, the authors suggest that Heinrich Event 1 could have been triggered by Siku Event 1.

Their results can be broken down into two main sections:

(1) The addition of freshwater—either 0.2 Sv or 0.5 Sv over 400 years—into the Northeast Pacific causes AMOC to weaken because of a decrease in the salinity and thus density of North Atlantic surface waters. Since the Bering Strait was closed at 19 ka BP, the authors suggest that this freshwater reaches the North Atlantic via the Indonesian Throughflow and Agulhas Leakage. The authors also rule out local changes in hydroclimate because there is a decreasing trend in precipitation over the North Atlantic.

(2) While these initial hosing experiments lead to a weakening of the AMOC, warming of the subsurface is restricted to latitudes south 50N, which is inconsistent with the classic view that warming of the northern North Atlantic subsurface causes Heinrich Events by triggering Laurentide Ice Sheet collapse. Consequently, the authors suggest (and then test this hypothesis with an additional hosing experiment [0.1 Sv released into the Northeast Atlantic over 200 years]) that subsurface warming in the Northeast Atlantic, due a weaker AMOC, caused the British Irish Ice Sheet to melt, releasing additional freshwater in the North Atlantic, which was sufficient to warm the northern North Atlantic surface and trigger a collapse of the Laurentide Ice Sheet consistent with Heinrich Events.

I thoroughly enjoyed reading this well-considered and well-written study, which applies a relatively novel concept to address a long-standing question. I am generally supportive of its eventual publication, provided the authors address the concerns and suggestions outlined below, especially my major concern.

We thank Reviewer #3 for the supportive feedback and helpful suggestions. We have carefully addressed all the concerns. Please see our responses to each comment below.

Main comments:

My major concern with the study is that is in unclear why this new and relatively

complicated Siku mechanism is needed as a trigger for Heinrich Events since the Bassis et al., (2017) hypothesis means we can explain Heinrich Events as paced by isostatic rebound linked to DO type events caused by cyclical changes in the Nordic Seas. Therefore, it is crucial that early on in the manuscript, the authors acknowledge that although plausible/viable mechanisms for explaining Heinrich Events exist, e.g., Bassis et al., 2017, this study wants to explore the potential, but not necessarily requisite, role of Pacific freshwater in Heinrich Events.

We agree with the reviewer that it is crucial to review existing mechanisms and explain the reason for exploring a new mechanism. In this revision, we review existing hypotheses, including the binge-purge mechanism, the North Atlantic subsurface warming, and the isostatic adjustment mechanism proposed by Bassis et al. (2017). Essentially, the isostatic adjustment mechanism plays a key role in regulating the exposure of Laurentide Ice Sheet marine terminus to subsurface warming, which appears to be a satisfactory triggering mechanism for Heinrich events. is not associated with large-scale North Atlantic warming because Greenland Ice core record shows simultaneous surface cooling (Andersen et al., 2004). Alternatively, an early AMOC weakening could drive subsurface warming through reduced downward mixing of surface cold water, leading to build-up of heat in the subsurface (He et al., 2020). Yet, the mechanisms driving AMOC weakening prior to Heinrich events remain unclear. Thus, a better understanding of the mechanism that caused AMOC weakening prior to Heinrich events is needed. With a consistent sequence of Cordilleran-BIIS-Laurentide meltwater events appearing in multiple Heinrich stadials, we seek to understand the potential role of Cordilleran ice sheet in global climate during Heinrich stadials. Please see our revised texts in line 56-88.

- Andersen, K. K., Azuma, N., Barnola, J.-M., Bigler, M., Biscaye, P., Caillon, N., Chappellaz, J., Clausen, H. B., Dahl-Jensen, D., & Fischer, H. (2004). High-resolution record of the Northern Hemisphere climate extending into the last interglacial period. *Nature, 431*, 147-151.
- He, C., Liu, Z., Zhu, J., Zhang, J., Gu, S., Otto-Bliesner, B. L., Brady, E., Zhu, C., Jin, Y., & Sun, J. (2020). North Atlantic subsurface temperature response controlled by effective freshwater input in “Heinrich” events. *Earth and Planetary Science Letters, 539*, 116247.

I also have concerns that this study is not explicit enough in addressing the major limitations, assumptions, and caveats associated with the study’s experimental design. We agree with the reviewer that the paper can be improved by more explicitly addressing the major limitations, assumptions, and caveats associated with the study’s experimental design. Please see our detailed responses to each of the concerns below.

For example, while the added freshwater flux, equivalent to 7.5m and 18.8m, is within bounds of total global sea level rise, it implies that nearly all global sea level rise was due to freshwater flux from the Cordilleran and/or Pacific sector of the LIS. However, previous work (e.g., Clark et al., 2004; Carlson & Clark, 2012; Toucanne et al., 2015) has argued for substantial amounts of melt at 18-19 ka BP from the BIIS and FIS, which is incompatible with the prescribed flux freshwater into the Northeast Pacific associated with Siku 1. Please address this inconsistency by including some additional text discussing the feasibility of the volumes of freshwater fluxes used in these hosing experiments with

respect to previous work, i.e., the references included above.

We thank Reviewer #3 for the concern over the feasibility of the volumes of freshwater fluxes used in our hosing experiments. We acknowledge that the imposed meltwater forcing in NP02 (7.5 m) and especially NP05 (18.8 m) may have overestimated the actual meltwater contribution from the Cordilleran Ice Sheet during S1, given that the total Cordilleran ice sheet volume is estimated to be ~8.8 m s.l.e. (Seguinot et al., 2016) and that melt from other ice sheets also contributed to the global sea-level change at 19 ka.

In this revision, we performed a new North Pacific hosing simulation with a 0.1 Sv freshwater flux (i.e., NP01). All three simulations, with 01, 0.2, and 0.5 Sv of freshwater flux, produce qualitatively similar responses, showing qualitatively similar responses, including freshwater transport pathway (Fig. 2; copied below) and North Atlantic subsurface temperature change (Fig. 4a-c; copied below). This suggests that the mechanisms related to the impact of North Pacific freshwater proposed in our study are robust and insensitive to hosing strength.

Furthermore, we would like to argue that an exaggeration of freshwater forcing intensity can be justified because the current generation of general circulation models is known to produce an overly stable AMOC (Valdes, 2011). For example, while H1 meltwater release from the Laurentide Ice Sheet has been estimated at only ~0.02 Sv (Zhou & McManus, 2024), substantially stronger forcings (0.25 to 1 Sv) are typically required to achieve atmospheric and oceanic responses that are consistent with proxy data (DiNezio et al., 2025; Lawman et al., 2025; Pöppelmeier et al., 2023). The models' over-stability may result from model biases in surface water salinity in the South Atlantic, which cause the models to have a net import of freshwater in the South Atlantic, contradicting the net export shown in observational data (Weijer et al., 2019). This bias salinifies the Atlantic Ocean when the AMOC is weakened, internally reinvigorating the AMOC when the external forcing is too small (Weijer et al., 2019). In this context, our slight exaggeration of freshwater forcing is not only justified, but also allows us to test the sensitivity and robustness of the AMOC's response to varying forcing intensities.

Please see our revised Methods section in line 357-390.

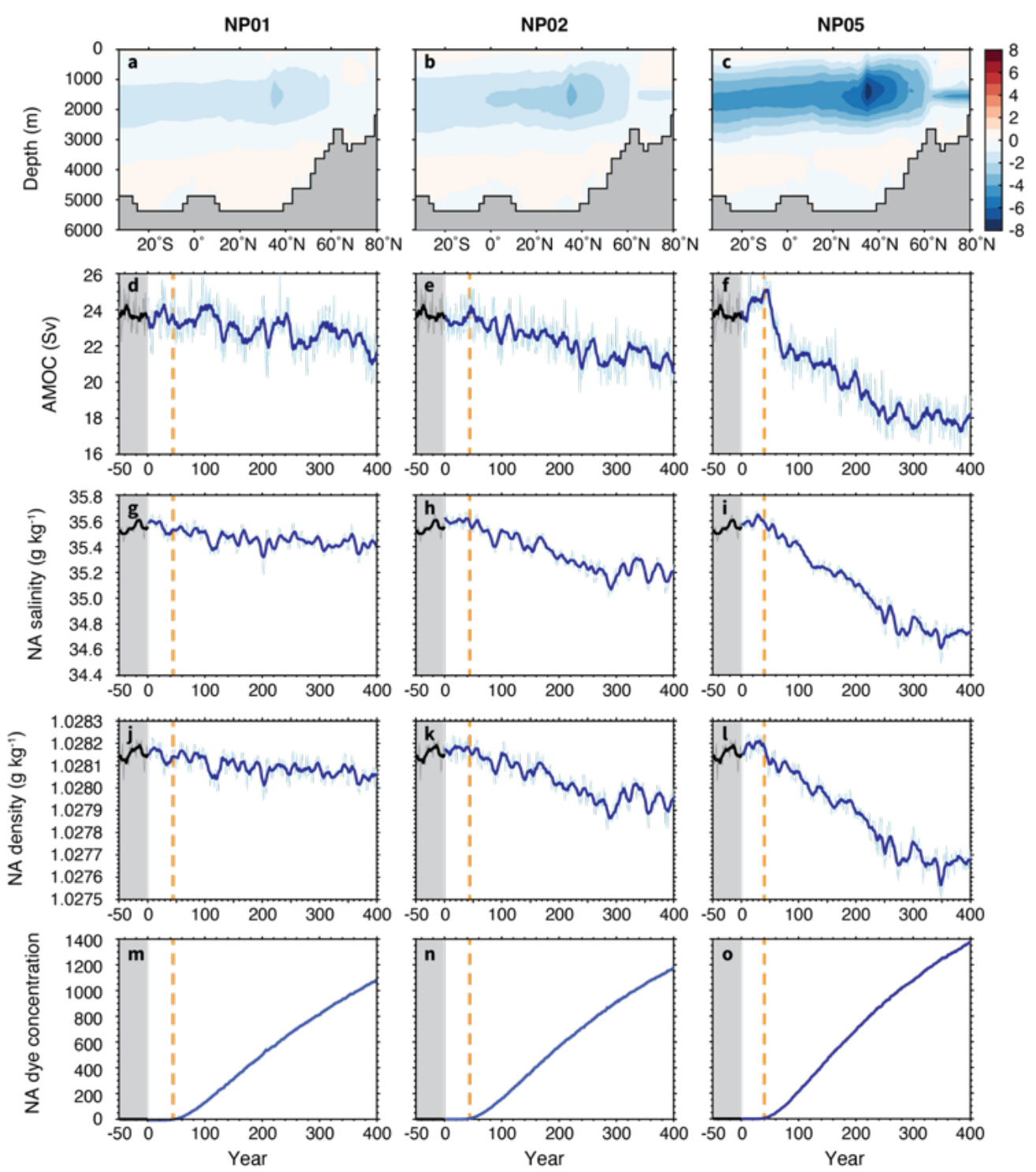

Figure 2 | Relationship between AMOC weakening and freshwater transport from the North Pacific. a-c) Changes in AMOC strength. **d-f)** Evolution of AMOC strength (defined as the maximum stream function between 10 and 60 ° N). **g-i)** Evolution of upper water (top 100 m) salinity in the North Atlantic (45-60°N). **j-l)** Evolution of upper water density in the North Atlantic. **m-o)** Dye tracer concentration in the North Atlantic upper water, indicative of water mass transport from the North Pacific. In panels d-o, the thin light-colored curves represent annual mean values, and the thick dark-colored curves represent 10-year moving average values. The gray bars indicate the last 50 years of the 19 ka control simulation. The orange dashed lines indicate the first appearance of the dye tracer in the North Atlantic.

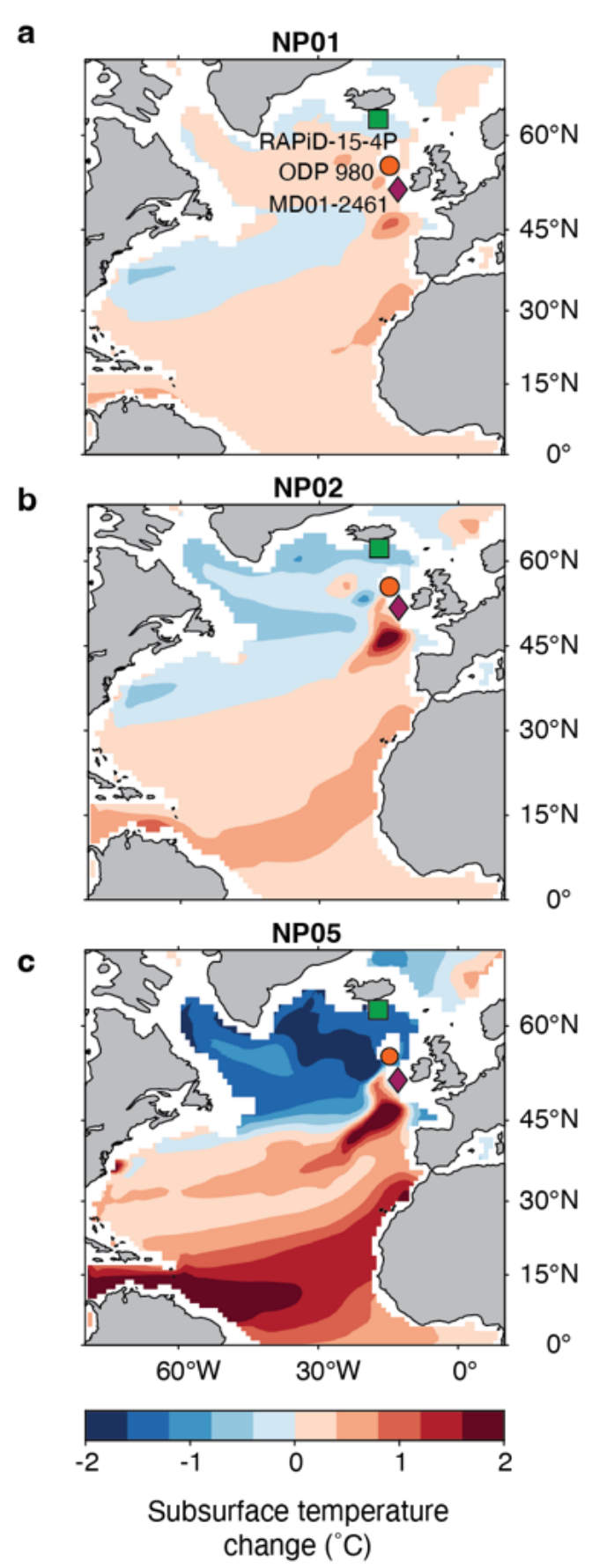

Figure 4 | a–c) Changes in annual mean North Atlantic subsurface temperature in a) NP01, b) NP02, and c) NP05. Mean values of 300–700 m depth are shown.

- DiNezio, P. N., Shanahan, T. M., Sun, T., Sun, C., Wu, X., Lawman, A., Lea, D., Kageyama, M., Merkel, U., Prange, M., Otto-Bliesner, B., & Zhang, X. (2025, 2025/08/01). Tropical response to ocean circulation slowdown raises future drought risk. *Nature, 644*(8077), 676-683.
- Lawman, A. E., Sun, C., Wu, X., Sun, T., Piatrunia, N., Gomez, K., Kageyama, M., Merkel, U., Prange, M., Otto-Bliesner, B., Zhang, X., DiNezio, P., & Shanahan, T. (2025, 2025/12/01/). Mechanisms driving the global tropical response to a weakened AMOC during Heinrich Stadial 1. *Quaternary Science Reviews, 369*, 109567.
- Pöppelmeier, F., Jeltsch-Thömmes, A., Lippold, J., Joos, F., & Stocker, T. F. (2023). Multi-proxy constraints on Atlantic circulation dynamics since the last ice age. *Nature Geoscience, 16*(4), 349-356.
- Seguinot, J., Rogozhina, I., Stroeven, A. P., Margold, M., & Kleman, J. (2016). Numerical simulations of the Cordilleran ice sheet through the last glacial cycle. *The Cryosphere, 10*(2), 639-664.
- Valdes, P. (2011). Built for stability. *Nature Geoscience, 4*(7), 414-416.
- Weijer, W., Cheng, W., Drijfhout, S. S., Fedorov, A. V., Hu, A., Jackson, L. C., Liu, W., McDonagh, E. L., Mecking, J. V., & Zhang, J. (2019). Stability of the Atlantic Meridional Overturning Circulation: A review and synthesis. *Journal of Geophysical Research: Oceans, 124*(8), 5336-5375.
- Zhou, Y., & McManus, J. F. (2024). Heinrich event ice discharge and the fate of the Atlantic Meridional Overturning Circulation. *Science, 384*(6699), 983-986.

Additional text is also required acknowledging that the hypothesised mechanistic relationship between Siku 1 and Heinrich 1 may not be valid for other earlier Heinrich Events, both in terms of the differing volumes freshwater flux (and global sea level rise) and variable boundary conditions during the last glacial period. For example, previous work, e.g., Hemming et al., 2004 (and references therein) & Roberts et al., 2014, suggests that during earlier Heinrich stadials there wasn't 15+ metres of sea level change. Would the volume of freshwater equivalent to a much smaller global sea level rise also result in the same sequence of downstream events leading to a Heinrich Event (e.g., subsurface warming in the Northeast Atlantic, BIIS melt etc)? This should be discussed explicitly within the text, including some acknowledgement that the prescribed volume of freshwater fluxes used in these hosing experiments is not applicable to all Heinrich Events. Similarly, there also needs to be more discussion of the varying boundary conditions during the last glacial period and how this impacts the proposed Northeast Pacific-North Atlantic connection. For example, it has been suggested that the Bering Strait was open from 46-35.7 ka BP (Farmer et al., 2022), so how would this have impacted the freshwater flux associated with Siku 4? Both these points raise the issue that the proposed mechanism is only applicable to Siku 1/Heinrich 1, and this must be acknowledged within the main text.

We thank Reviewer #3's comment on the validity of the proposed mechanistic relationship between Siku 1 and Heinrich 1 for other earlier Heinrich Events. In the revised Summary and Discussion section, we expand our discussion on the implications for other Heinrich Stadials. We acknowledge that all Heinrich Stadials are not the same, with each associated with distinct forcings and boundary conditions. We argue that the repeating sequence of Siku Events, BIIS meltwater discharge, and Heinrich Events during HS2-4 at

least suggests it is plausible that this mechanism applied to these earlier events. We further discuss the possible scenarios specific to Heinrich 3 and 4. We also indicate that future studies need to evaluate Heinrich stadials on a case-by-case basis, including event-specific forcings and boundary conditions. Please see revised text in line 270-295, which we copy below.

“Although our study focuses on HS1, the recurring sequence of Siku Events, BIIS meltwater discharge, and Heinrich Events during HS2-4 according to available proxy records (Figure 1) suggests that a similar mechanism may be plausible for these earlier events as well. However, we acknowledge that all Heinrich stadials are not the same, with each associated with distinct forcings and boundary conditions. For example, Farmer et al. (Farmer et al., 2023) present evidence for an open Bering Strait during HS4, which is different from our model setup with a closed Bering Strait for HS1. Using a high-resolution ocean model with an open Bering Strait, Praetorius et al. (Praetorius et al., 2020) demonstrated that North Pacific freshwater could be transported to the North Atlantic via the Arctic route within a decade, leading to cooling in the North Atlantic. This Arctic pathway is likely more efficient than the Pacific–Indian–Atlantic route proposed for HS1 in this study and is equally, if not more effective, at triggering AMOC weakening. Given that the European Ice Sheets were significantly smaller during HS4, Pacific-sourced meltwater may have played a larger and more direct role in triggering H4 than it did during HS1. Meanwhile, H3 represents a small and atypical Heinrich event, with Ruddiman Belt IRD dominated by European sources (Gwiazda et al., 1996; Hemming, 2004). Our North Pacific hosing simulations suggest that even with a weak North Pacific freshwater forcing, the European ice sheet could still be impacted and lead to H3. However, these hypotheses remain to be tested with further studies, as our results show that the northern North Atlantic subsurface temperature response and the potential impacts on the Laurentide Ice Sheet are sensitive to the combined influences remotely from the North Pacific freshwater and locally from the European Ice Sheets. This highlights the need for future studies to evaluate Heinrich stadials on a case-by-case basis, accounting for event-specific forcings and boundary conditions. Expanded spatiotemporal coverage of proxy data, such as seawater chemical and physical properties, will be critical for further constraining such modeling efforts. Similarly, advances in next-generation eddy-resolving high-resolution models will improve the representation of narrow boundary currents, convections, and eddies, processes which play a key role in freshwater transport and AMOC strength (Jackson et al., 2023; Shan et al., 2024).”

- Farmer, J. R., Pico, T., Underwood, O. M., Cleveland Stout, R., Granger, J., Cronin, T. M., Fripiat, F., Martínez-García, A., Haug, G. H., & Sigman, D. M. (2023). The Bering Strait was flooded 10,000 years before the last glacial maximum. Proceedings of the National Academy of Sciences, 120(1), e2206742119.
- Gwiazda, R., Hemming, S., & Broecker, W. (1996). Provenance of icebergs during Heinrich event 3 and the contrast to their sources during other Heinrich episodes. Paleoceanography, 11(4), 371-378.
- Hemming, S. R. (2004). Heinrich events: Massive late Pleistocene detritus layers of the North Atlantic and their global climate imprint. Reviews of Geophysics, 42(1).

- Jackson, L. C., Hewitt, H. T., Bruciaferri, D., Calvert, D., Graham, T., Guiavarc'h, C., Menary, M. B., New, A. L., Roberts, M., & Storkey, D. (2023). Challenges simulating the AMOC in climate models. Philosophical Transactions of the Royal Society A, 381(2262), 20220187.
- Praetorius, S. K., Condron, A., Mix, A. C., Walczak, M. H., McKay, J. L., & Du, J. (2020). The role of Northeast Pacific meltwater events in deglacial climate change. Science advances, 6(9), eaay2915.
- Shan, X., Sun, S., Wu, L., & Spall, M. (2024). Role of the Labrador Current in the Atlantic Meridional Overturning Circulation response to greenhouse warming. Nature communications, 15(1), 7361.

Although a 1 degree resolution ocean model is good, it is not as high as some state of the art ocean models used in similar studies. Does model resolution have any impact on freshwater routing/spreading pathways? Can you leverage the results from other modeling studies looking at spreading pathways in the ocean, e.g., Condron et al., 2009, to help make the case the freshwater routing in this study is robust and not model dependent? Please include an additional paragraph discussing how simulations using a higher resolution model may (or may not) impact the results of this study. This paragraph should also include a sentence acknowledging that the impact of freshwater on AMOC is dependent on model resolution, e.g., Shan et al., 2024.
We thank the reviewer for this suggestion to use high resolution model to support the freshwater routing in this study. Indeed, Praetorius et al. (2020) previously used the 1/6° MITgcm ocean model to run North Pacific hosing simulations with an open/closed Bering Strait. In their closed Bering Strait simulation, which is similar to our glacial boundary condition setup, the freshwater is transported to the tropical Pacific within 10 years, driving a local sea surface salinity anomaly. In this revision, we cite this simulation to support the similar routing and speed observed in our simulation. However, their simulation is run for only 10 years, so it does not provide a complete view of global transport of the freshwater. Please see our revised text in line 124-127.

In their closed Bering simulation, the freshwater is quickly transported to the North Atlantic within 10 years through the Arctic Ocean, which leads to significant cooling in the North Atlantic. We infer that this North Atlantic SST cooling might be related to an AMOC response to the freshwater. We cite this simulation to infer the potential impact of North Pacific freshwater during Heinrich Stadial 4, when the Bering Strait was likely open. Please see our revised text in line 276-282.

To the best of our knowledge, there are very few existing North Pacific hosing simulation hosing simulations, and Praetorius et al. (2020) is the only one that utilize a high resolution model. In our Summary and Discussion, we suggest that advances in next-generation eddy-resolving high-resolution models will improve the representation of narrow boundary currents, convections, and eddies, processes which play a key role in freshwater transport and AMOC strength (Jackson et al., 2023; Shan et al., 2024). Please see our revised text in line 292-295.

Invoking the BIIS as an intermediary step is well argued and supported with hosing experiment BIIS01, however, after that, the mechanism is a bit more ad hoc and there is no

consideration given to the potential impact of additional FIS melt and associated freshwater input into the northern North Atlantic. Please make this section clearer and include some additional text acknowledging other potential impacts and uncertainties associated with BIIS melt.

We thank Reviewer #3 for this suggestion. In this revision, we report 4 hosing simulations to test the impact of BIIS meltwater to assess other potential impacts and uncertainties associated with BIIS melt:

- **NP02_BIIS01:** previously named BIIS01 in our initial submission, where 0.1 Sv of freshwater forcing is added to the Irish west coast, branched from the climate of year 400 of NP02
- **ctrl_BIIS01:** new simulation branched from the climate of iTRACE 19ka, with 0.1 Sv of freshwater forcing added to the Irish west coast.
- **NP02_BIIS005:** new simulation branched from the climate of year 400 of NP02, but with only 0.05 Sv added to the Irish west coast.
- **ctrl_BIIS005:** new simulation branched from the climate of iTRACE 19ka, but with only 0.05 Sv of freshwater forcing added to the Irish west coast.

Running both NP02_BIIS01 and NP02_BIIS005 allows us to evaluate the sensitivity of the AMOC to BIIS meltwater. This comparison shows that 0.05 Sv of freshwater forcing is too weak. Additionally, we notice that both simulations show subsurface warming in the Nordic Seas, which could potentially drive more freshwater release from the FIS, further weakening the AMOC. Comparing simulations run with different initial conditions (NP02_BIIS01 vs ctrl_BIIS01 and NP02_BIIS005 vs ctrl_BIIS005) allows us to assess the how preconditioning of the North Atlantic with Siku meltwater could modulate the AMOC response to BIIS meltwater. Please see our revised text in line 206-254 and methods in line 393-420.

Since the Bering Strait was closed at 19 ka BP, the authors suggest that freshwater from S1 reaches the North Atlantic via the Indonesian Throughflow and Agulhas Leakage. While this routing is consistent with modelling/observational data and supported by planktic d18Osw records from the southern tip of Africa, more paleo evidence from other sites along the proposed flow path are necessary to fully constrain this long and complex routing, e.g., data from the Pacific Ocean, Indian Ocean, and/or South Atlantic. Or if such records are not available, then this should be acknowledged in the text, e.g., 'paleo evidence in support of this freshwater routing is limited to the southern tip of Africa'.
We thank Reviewer #3 for this helpful suggestion. In this revision, we have included a new Figure 3 that compiles syntheses of d18Osw from NE Pacific, NW Pacific, Indonesia Throughflow, and Agulhas. Table S2 provides the detailed information of each record included. Please see our revised text in line 137-152, which is copied below:
"The proposed meltwater transport pathway is also supported by proxy evidence. Since ice sheet meltwater is isotopically lighter than seawater, planktic foraminiferal oxygen isotope data corrected for temperature and global ice volume changes ($\delta^{18}O_{sw-ivc}$) can be used to track freshwater transport in the upper ocean. Near the meltwater source in the Northeast

Pacific, a previously reported compilation of $\delta^{18}O_{sw-ivc}$ records shows a negative shift of up to -1.2‰ around 19 ka [28] (Fig. 3c,h), and the signal is carried over to the Northwest Pacific (Fig. 3d,i). In the Indonesian Throughflow, the key region where Pacific waters flow into the Indian Ocean, a number of $\delta^{18}O_{sw-ivc}$ records also exhibit a negative shift during S1 [e.g. 35] (Fig. 3e,j), even though $\delta^{18}O_{sw-ivc}$ records from this region can be complicated by the dynamic deglacial hydroclimate variability [3,36], sea level change [37], and age model uncertainty [35]. Additional support comes from a negative shift of ~-0.5‰ in a subsurface $\delta^{18}O_{sw-ivc}$ record based on the upper thermocline species *P.obliquiloculata*, whose isotopic composition is less affected by surface hydrological changes [38], consistent with transport of isotopically light Pacific waters through the Indonesian Throughflow. Further downstream, a negative shift in $\delta^{18}O_{sw-ivc}$ is observed in records from the southern tip of Africa (Fig. 3f,k), suggesting increased freshwater flow into the Atlantic Ocean via the Agulhas Leakage."

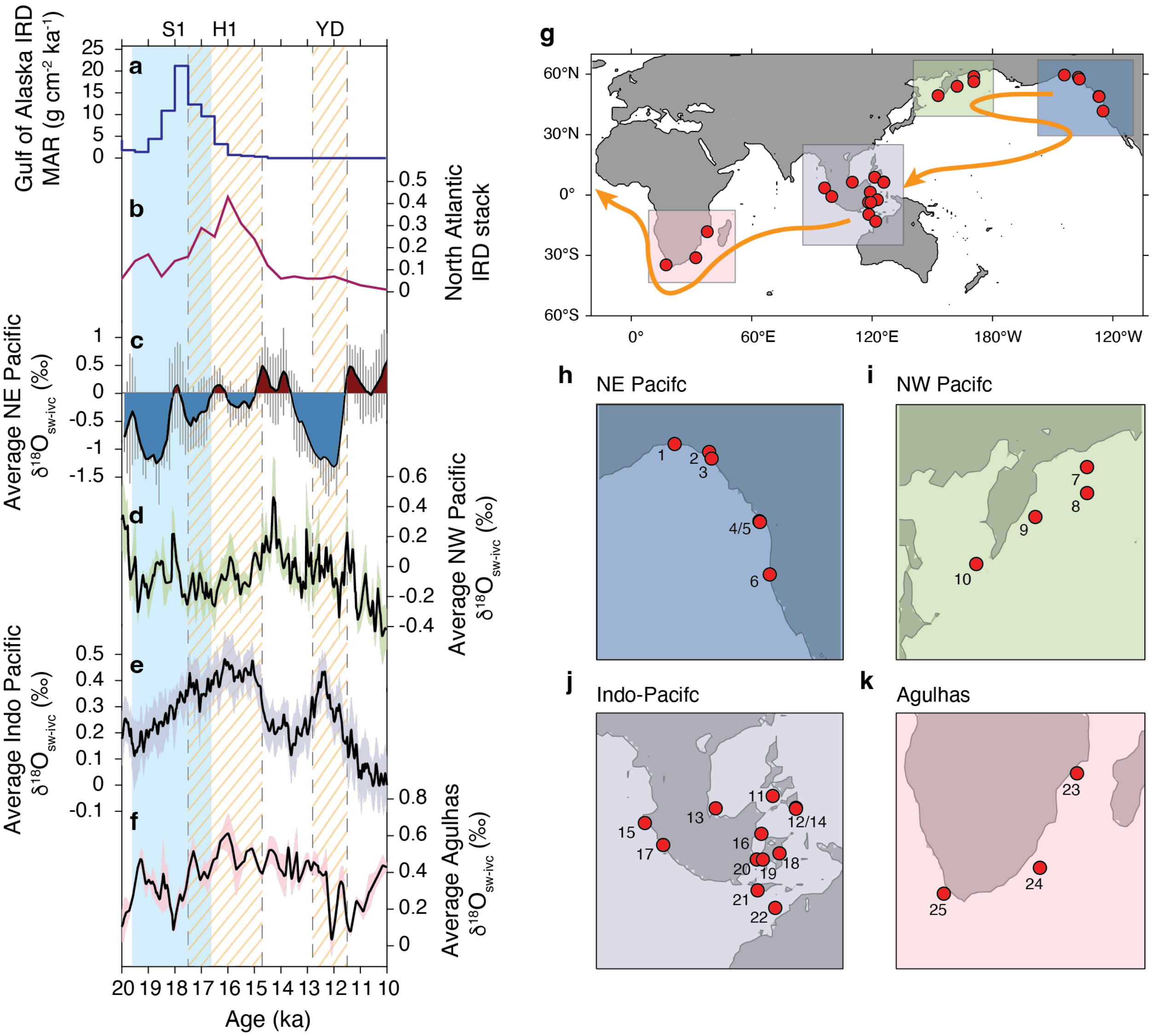

Figure 3 | Proxy evidence for surface freshening along the Pacific-Indian-Atlantic freshwater transport pathway during S1. a) IRD record from U1419 in the Northeast Pacific [27]. **b)** North Atlantic IRD stack [66]. **c)** Average of the $\delta^{18}O_{sw\text{-}ivc}$ records from the Northeast Pacific synthesized by Praetorius et al. [28]. **d)** Average of the $\delta^{18}O_{sw\text{-}ivc}$ records from the Northwest Pacific. **e)** Average of the $\delta^{18}O_{sw\text{-}ivc}$ records from the Indo-Pacific Warm Pool region. **f)** Average of the $\delta^{18}O_{sw\text{-}ivc}$ records from the Agulhas Leakage region. To calculate the averages in d-f, each individual record is linearly interpolated to a 20-year resolution and the mean deglacial values (20–10 ka) is removed prior to averaging. The color shadings denote $\pm\sigma$, with the color corresponding to highlighted regions in panels g-k. The vertical blue bar across the panels on the left highlights S1; the orange hatched bars highlight H1 and Younger Dryas. **g)** A map showing the locations of all $\delta^{18}O_{sw\text{-}ivc}$ records included. **h-k)** Zoomed in maps showing the locations of $\delta^{18}O_{sw\text{-}ivc}$ records from h) the Northeast Pacific; i) the Northwest Pacific; j) the Indo-Pacific Warm Pool; and k) the Agulhas Leakage regions. The numbers next to the site locations correspond to the site numbers in Table S2.

Related to this, the main text would benefit from a more comprehensive description of how freshwater originating in the Northeast Pacific reaches the subpolar North Atlantic. At present the description is limited to 2/3 lines in the main text, with some additional information in the SI, the latter of which contains some really useful context for the reader. Given the relative brevity of the manuscript (which is definitely not a bad thing), can you incorporate this inter-basin freshwater transport text into the main text?
We thank Reviewer #3 for this helpful suggestion. We have incorporated this inter-basin freshwater transport text into the main text to provide more detail. Please see our revised text in line 114-120, copied below:

"Since the Bering Strait is closed at 19 ka, the freshwater transport takes a westward Pacific-Indian-Atlantic surface pathway: the freshwater is quickly mixed and transported to the tropical Pacific by the subtropical gyre, entering the Indian Ocean through the Indonesian Throughflow by model year 10 (Fig. S5, see Supplementary Information). By model year 15, the water mass enters the South Atlantic via the Agulhas Leakage. In the Atlantic Ocean basin, the freshwater travels northward, eventually reaching the North Atlantic in model year 40-50."

Thanks for a really fascinating study!
We thank Reviewer #3 again for the positive feedback and constructive suggestions!

Line by line comments:

L38: 'variability' instead of 'strength' might be better?
Thanks. We have made the change as suggested

L39: Include a reference to the RAPiD array at 26.5oN,
e.g., https://www.bodc.ac.uk/data/published_data_library/catalogue/10.5285/223b34a3-2dc5-c945-e063-7086abc0f274/
Thanks. We have included this reference here as suggested.

L55/56: Unclear, please reword, e.g., ...'similar to the observed retreat of the West Antarctic ice today'.

Thanks. We have reworded the text as suggested.

L91-94: Where is the evidence for a decreasing trend in precipitation over the North Atlantic?

Thanks. We have included a new Figure S4, copied below:

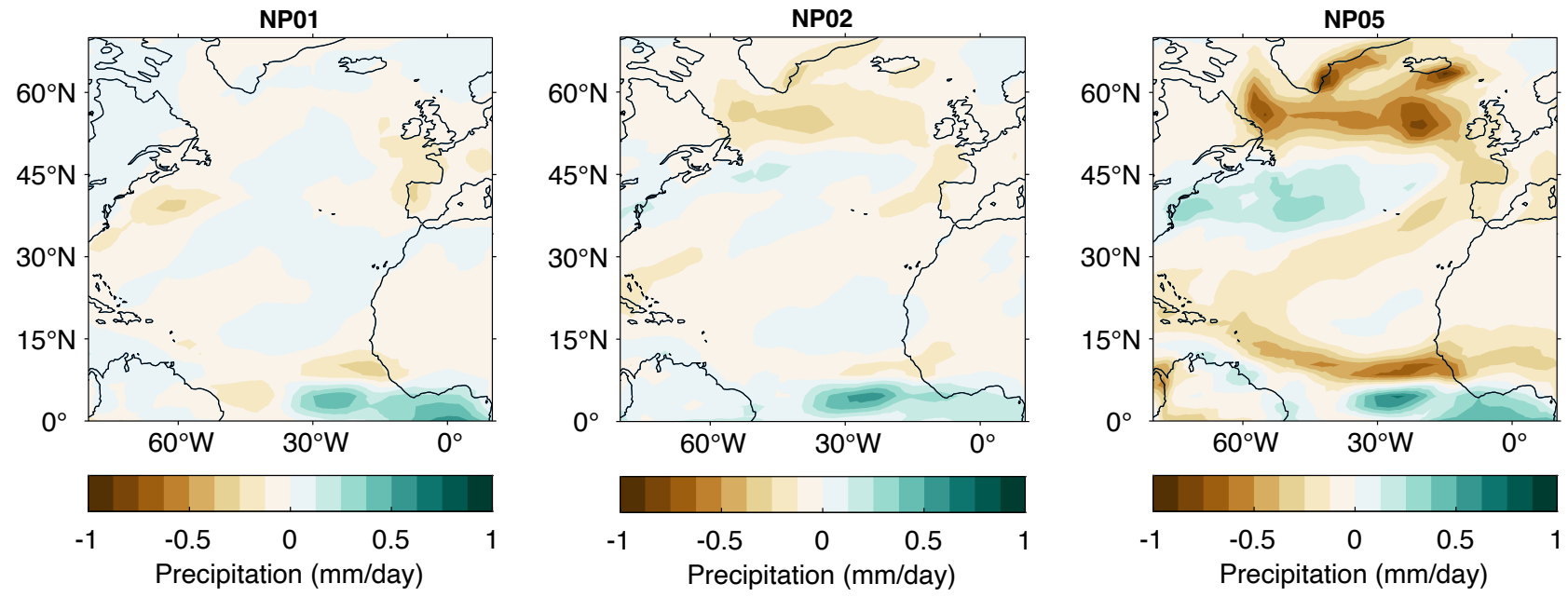

Figure S4 | Changes in mean annual precipitation (mm/day) over the North Atlantic in NP01, NP02, and NP05 relative to the 19ka control.

L102: Not sure I agree with the suggested positive feedback though – would cooling not also increase the density of surface waters, thereby sustaining/facilitating deep water formation?

Thanks. We have revised the text in line 120-122 to clarify this feedback:
"A weaker AMOC is amplified by a positive feedback associated with expanded sea ice, which increases meltwater fluxes when North Atlantic sea ice melts during summer (Fig. S6-7)." It can clearly be seen in Figs S6 and S7 that there is an anomalous freshwater flux under the North Atlantic sea ice region, which reduces the salinity and density of surface waters. Although cooling could increase the density, its impact is smaller than the meltwater flux.

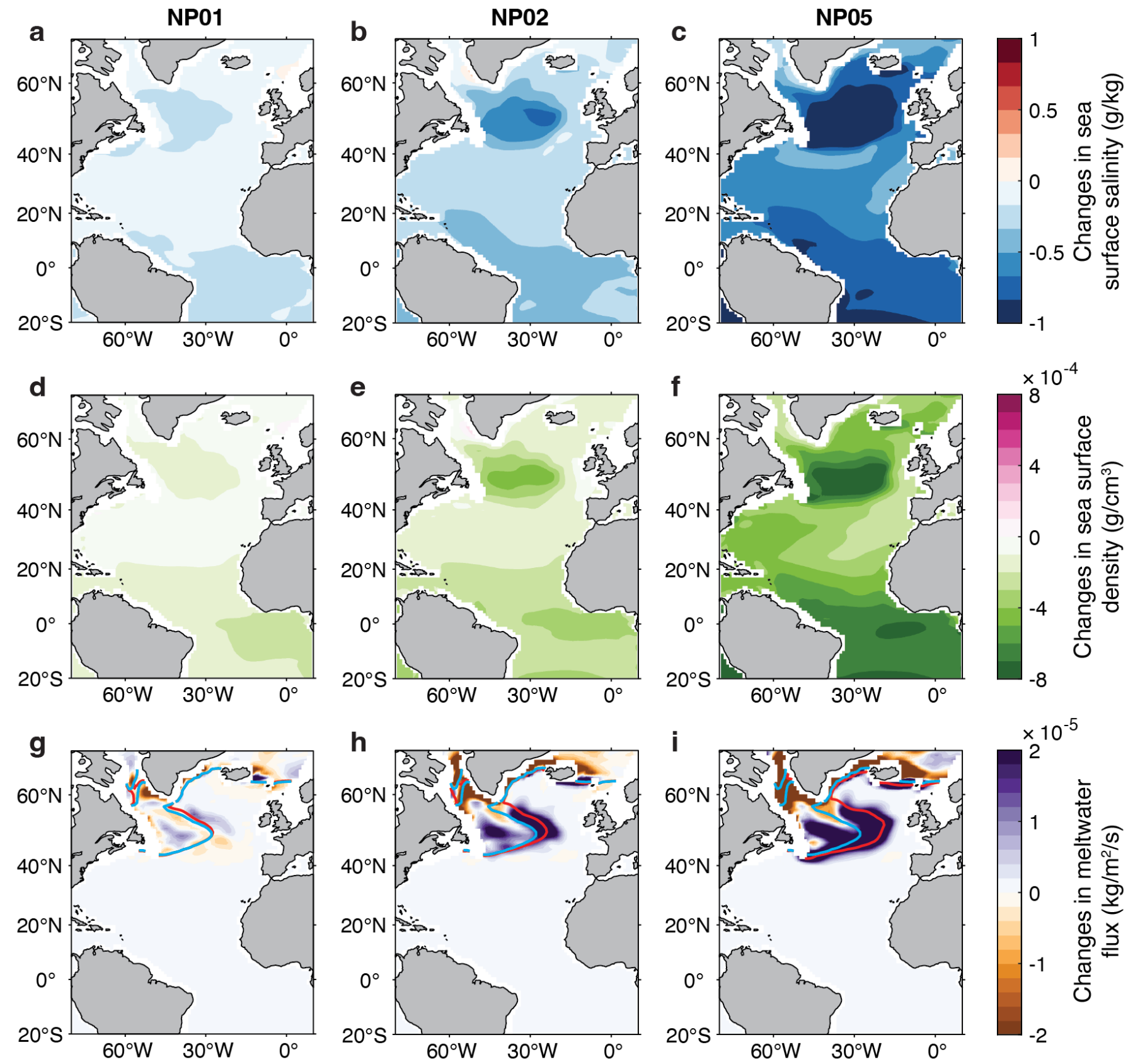

Fig. S6. Maps of mean annual changes in the North Atlantic, including **a-c)** sea surface salinity, **e-g)** sea surface potential density, and **h-j)** sea ice meltwater fluxes in NP01, NP02, and NP05 relative to the 19ka control. In h-j, the blue and redlines indicate February sea ice margins (defined as the 15% sea ice coverage) in the 19 ka control hosing simulations.

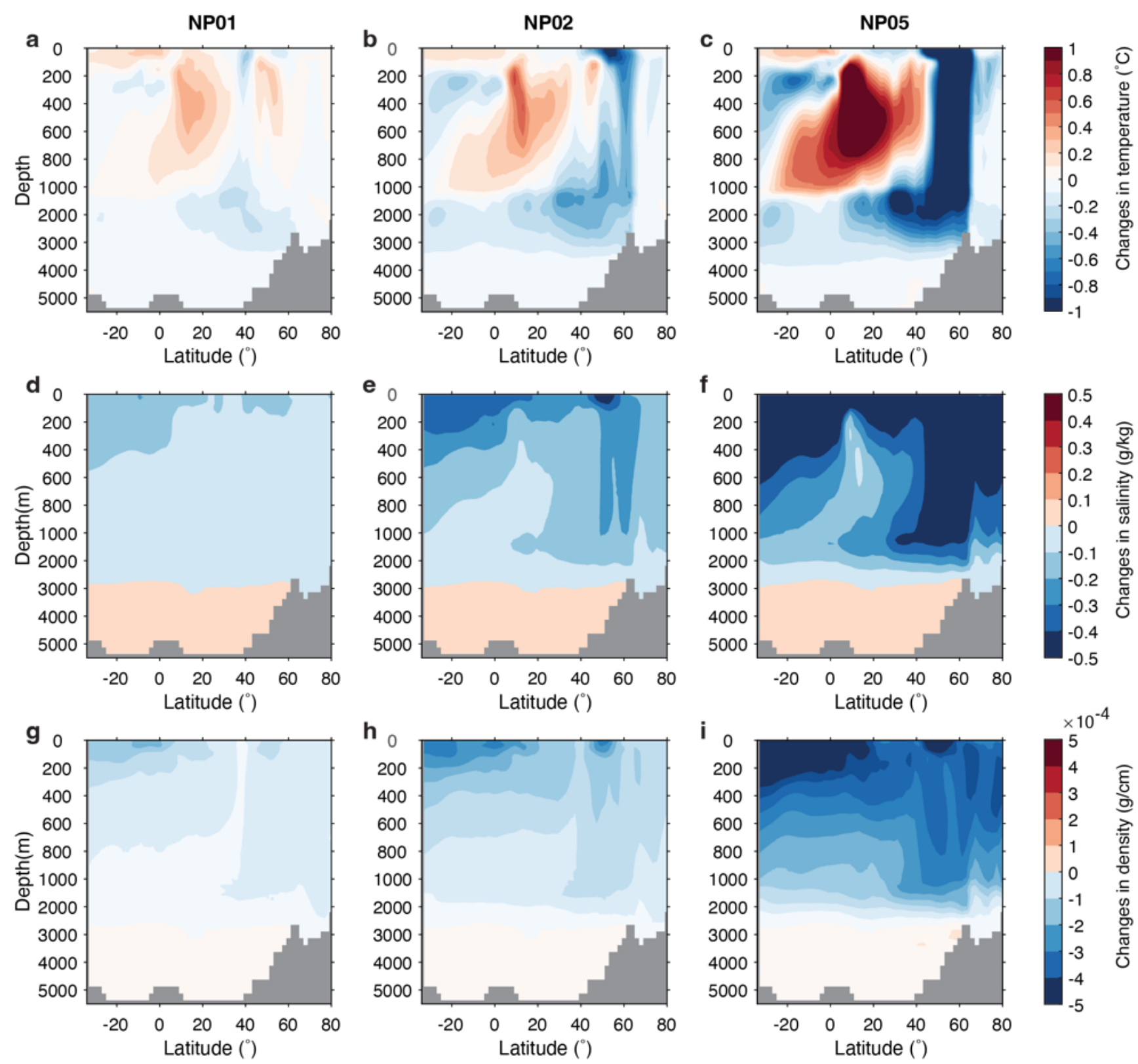

Fig. S7. Zonally averaged sections of changes in the Atlantic Ocean, including **a-c)** temperature anomalies, **d-f)** salinity anomalies and **g-i)** potential density anomalies in NP01, NP02, NP05 relative to the 19ka control.

L116-117: '…represents a robust physical process supported by other modeling studies as well as modern observations of surface water mass transport'. It looks like you refer to the modern observational data before the climate modeling work, so flip the order to reflect that in this set-up sentence.

Thanks. We have made the change following Reviewer #3's suggestions. Please see revised text in line 124-129.

L117: Can you also be a bit more specific about the source of these conclusions, e.g., at least mention the use of ECCOv4?

Thanks for this suggestion. ECCOv4 is cited here as the source of these conclusions. Please see revised text in line 126-127.

L120: I think the point here is that the Rousselet et al., 2020 study is supportive of an Indonesian Throughflow origin, but is there any evidence that these waters originated in the Northeast Pacific?

We thank reviewer #3 for this question. In this revision, we cite Praetorius et al. (2020)'s hosing study using the eddy-permitting MITgcm, which shows that Northeast Pacific freshwater is transported into the Northwest Pacific along boundary currents and subsequently entrained into the North Pacific current, which recirculates it back to the eastern margin and then southwestward towards the western tropical Pacific.

L124: What model? Can you be more specific again please?

We thank reviewer #3 for this question. The model used is the fully coupled COSMOS model run by Maier et al. (2018). Their Extended Figure 5 (copied below) exhibits an SST pattern consistent with the canonical hemispheric temperature seesaw associated with AMOC weakening. However, we have removed this part of the text in this revision because the AMOC response is not explicitly discussed in that paper.

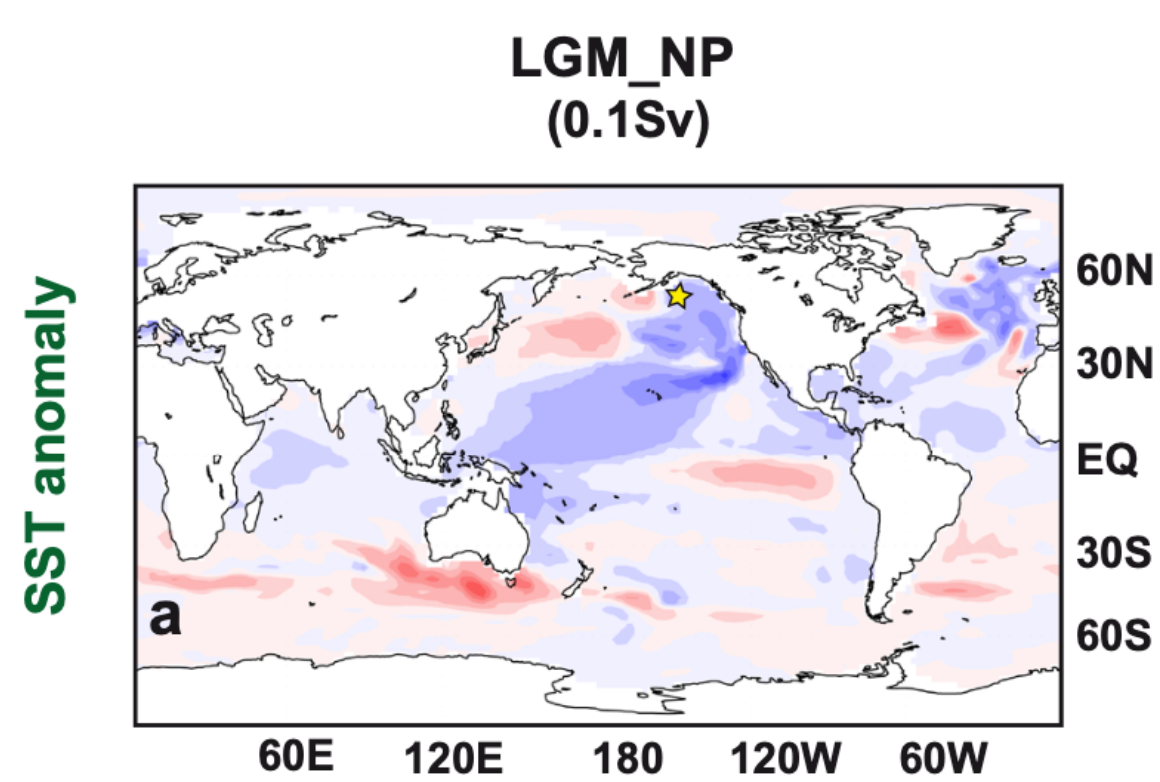

L144: Missing a 'the', e.g., 'warms the northern North Atlantic'.

Thanks. We have made the change.

L146: 'cooling prevails at both surface and subsurface' should be 'cooling prevails in both the surface and subsurface'.

We thank reviewer #3 for the helpful guidance on the language. We have made this change.

L194: This feels more like a conclusion then implications section rather than a discussion
We thank reviewer #3 for this comment. We have changed the section title from "Discussion" to "Summary and Implications".

We thank Reviewer #3 once again for the thoughtful comments and suggestions, which have greatly strengthened our manuscript. We hope that our revisions satisfactorily address all the concerns.

RESPONS TO REVIEWER COMMENTS

Reviewer #1 (Remarks to the Author):

Main comments:
This is my second review of “Triggering of Heinrich events by Cordilleran Ice Sheet meltwater into the North Pacific” by Sun and others. I thank the authors for incorporating the comments into the revised manuscript. My major concern related to the magnitude of freshwater hosing associated with ice sheet dynamics. While the authors argue that the current generation of general circulation models is known to produce an overly stable AMOC, which may be a fair point.

Overall, I do find this to be a valuable study from a mechanistic and global perspective. The work highlights the complexity of the Earth system by demonstrating how ice-sheet changes in different regions can generate interconnected climate response (mainly ocean responses here) across multiple ocean basins. However, since this study does not explicitly simulate the triggering of the BIIS or Heinrich event 1 associated with ice sheet dynamics, I would be cautious about making definitive statements regarding “triggering the Heinrich events” (for example, Lines 69-70, Lines 99-100, Line 163 ...). It may be more appropriate to focus on the mechanism related to North Atlantic subsurface warming. Other mechanisms proposed to trigger Heinrich events cannot be ruled out.

We thank reviewer #1 for this suggestion.

In line 69-70, we removed the word “triggering”.

Line 99-100 is changed to: “Building upon these results, we perform additional simulations to investigate how S1 meltwater, in conjunction with European Ice Sheet melt, could have impacted North Atlantic cryosphere leading up to Heinrich Event 1”

For the subsection title in line 163, we changed it to “AMOC weakening amplified by BIIS retreat and Heinrich Event 1”

Line 313: the role of S1 meltwater in triggering H1 => the role of S1 meltwater during H1

Line 354: we changed “triggering AMOC weakening” to “weakening the AMOC”

We have also changed the title to: North Pacific meltwater weakens the Atlantic Meridional Overturning Circulation and preconditions Heinrich Stadial 1

Line 137-152: The meltwater transport pathway and the comparison with proxy evidence. I appreciate the efforts. However, it remains unclear to what extent the signals can be attributed to the S1 event: (1) the maxima don't match between the IRD and the average NE and NW Pacific records (Fig. 3a,c,d) during 20-17 ka; decreases in the freshwater proxy signal occur earlier than the S1 event. (2) phase changes among different proxy records also differ substantially. The response times indicated by modelling across basins are on the order of tens to hundreds of years, whereas such delays are not evident in the proxy records. (3) there appears to be a strong signal during the YD in the NE pacific record. What is the cause of this signal? Could another Siku event have occurred during this period? If so, why do the Indo-Pacific records exhibit opposite phase changes? Overall, during the deglaciation period, changes in these delta18O records could be complex, given the large changes in orbital configuration, greenhouse gas concentrations, North Hemisphere ice sheets, and associated large-scale precipitation and hydroclimate changes. This needs to be interpreted carefully and should not be attributed solely to the Siku event.

We agree that these records need to be interpreted more carefully and should not be attributed solely to the Siku event. To address these concerns, we made the following revision:

"In the Northeast Pacific, an average of $\delta^{18}O_{sw\text{-}ivc}$ records shows a negative shift of up to -1.2‰ around 19 ka [29] (Fig. 3c,h), consistent with the discharge of isotopically light meltwater from the Cordilleran Ice Sheet. The apparent chronological mismatch between the peak isotope shift and IRD accumulation likely reflects challenges associated with dating of marine sediments, as well as inherently large uncertainties associated with the $\delta^{18}O_{sw\text{-}ivc}$ proxy system and its interpretation, including those due to Mg/Ca-SST quantification, ice volume estimation, and variability in ocean current, hydroclimate, and meltwater $\delta^{18}O$. A negative isotope shift during S1 is also observed at key downstream locations, including the Northwest Pacific (Fig. 3d,i), the Indonesian Throughflow region (Fig. 3e,j), and along the Agulhas Current south of Africa (Fig. 3f,k). Although we cannot rule out the influence of other potential mechanisms in driving these isotope changes, given the proxy uncertainties, the broadly synchronous isotope depletion between 19 and 17 ka is consistent with the meltwater transport mechanism proposed in this study."

We did not explicitly address the comments related to the YD in the paper because YD is not the focus of the paper. To answer Reviewer #1's question, Walczak et al. (2020) did not find YD-aged IRD in the Northeastern Pacific, likely because the Cordilleran Ice Sheet was substantially smaller during YD with limited marine terminus. d18Osw at the Indo-Pacific is likely influenced by hydroclimate change in association with an El Nino-like change over the equatorial Pacific, which drives drying and more positive d18O values (e.g. Hällberg et al., 2022).

Line 157-161: "... reconciling the conundrum that AMOC weakening occurred prior to H1 without the presence of Laurentide meltwater discharge". Not sure about this statement. In your NP02 experiment, the AMOC strength decreases to about 21 Sv, which is still quite vigorous, whereas in NP05, the freshwater input of 0.5 Sv seems excessively large. Moreover, increasingly recent studies suggest that millennial-scale oscillations may arise from changes in boundary conditions, such as orbit-CO2-ice sheet configurations, and this cause during the studied period cannot be ruled out. Additionally, during 19-17ka, insolation was already quite high, making it highly likely that the Eurasian ice sheets (including not only the BIIS but also the Fennoscandian and Barents-Kara Sea ice sheets) began to melt (e.g., Patton et al., 2017), which should also not be ignored.

We thank Reviewer #1 for this helpful feedback. We removed the quoted statement. This paragraph is currently read as " The robust responses in our North Pacific hosing simulations, as well as model-data agreement suggest that the Pacific meltwater may have driven AMOC weakening at the onset of HS1. This mechanism does not require freshwater forcings directly from the North Atlantic (e.g., meltwater from the Laurentide or European Ice Sheets). Although the AMOC is not fully shut down and the changes are relatively small in our S1 simulations, it is consistent with the reconstructed AMOC evolution showing that AMOC weakening was gradual at the onset of HS1 (Figure 4d) [7,8]."

We also agree with Review #1 that these other mechanisms should not be ignored.

In line the introduction, we make the following revision: "Modeling studies suggest that substantial AMOC weakening can result from variations in orbital forcing, CO_2, and ice sheet configurations (Yin et al., 2021; Zhu et al., 2017; Liu et al., 2025); however, these mechanisms are more relevant for Heinrich stadials at glacial terminations or inceptions rather than all Heinrich stadials. Sedimentary records have also suggested that meltwater from the European Ice Sheets could have contributed to AMOC weakening [10,22-25], however, this hypothesis remains debated due to the lack of consistent correspondence between European Ice Sheet melting and Heinrich events in proxy records[26,27]."

We also added a paragraph in the summary with a revised Supplementary Figure S2 to address this shortcoming: "This study does not consider the impacts of solar insolation, CO_2, and ice sheet. Given that these forcings evolve gradually and slowly at the early HS1, they are unlikely to explain the AMOC weakening at the onset of HS1. Using the iTRACE factorized-forcing simulations, we find that the magnitude of AMOC weakening due to insolation, CO_2, and ice sheet at 19 ka are considerably smaller than the response in our North Pacific freshwater hosing simulations (Fig. S2), highlighting an important role for S1 meltwater in weakening the AMOC at the onset of HS1. That said, all these mechanisms are not mutually exclusive. In particular, increasing summer insolation during the deglaciation may have enhanced ice sheet melting and freshwater input (Hemming 2004; Patton et al., 2017), providing an additional freshwater forcing for AMOC weakening."

Other comments:

Line 69-70: This sentence sounds weird. The precursory subsurface warming is one of the proposed mechanisms; describing it as “satisfactory” maybe too subjective.
We changed the sentence to “Thus, the precursory subsurface warming provides a viable mechanism for Heinrich events”

Line 108-111: “precipitation decreases over the northern North Atlantic NADW formation site”. How does continental runoff into the North Atlantic change? Which could potentially also be linked with atmospheric mechanisms.
Indeed, runoff could change as an atmospheric response. In this revision, we thoroughly investigated this process, including precipitation, evaporation, runoff, and ice runoff from land snow capping using our model output (Fig. S7). We have revised the text accordingly: “Additionally, although atmospheric processes may also affect salinity by altering regional hydroclimate, their influence is likely secondary. Cooling over the North Atlantic reduces precipitation and runoff, which is largely balanced by reduced evaporation (Fig. S7) and dwarfed by those from the sea ice meltwater flux (Fig. S5).”

Reference:
Patton, H., Hubbard, A., Andreassen, K., Auriac, A., Whitehouse, P. L., Stroeven, A. P., ... & Hall, A. M. (2017). Deglaciation of the Eurasian ice sheet complex. Quaternary Science Reviews, 169, 148-172.

Reviewer #2 (Remarks to the Author):

The authors have carefully addressed my major comments. A discussion on the freshwater release corresponding to a certain ice sheet volume and the possible magnitude of this ice sheet volume has been properly addressed for both the Cordilleran Ice Sheet and the British-Irish Ice Sheet. The author’s also corresponded to my request for additional simulations. Therefore I would like to support this preprint for publication in Nature Communications.
We would like to thank Reviewer #2 again for their support and helpful feedback.

Reviewer #3 (Remarks to the Author):

The authors have done an excellent job addressing all of my comments. I am particularly impressed with the inclusion of the additional model runs and thorough proxy compilation, as well as their thoughtful responses. Therefore, I recommend publication of this paper.
We would like to thank Reviewer #3 again for their support and helpful feedback.

Apart from a couple of small editorial suggestions, my only very minor point is that the authors should include a line in the main text acknowledging the use of exaggerated freshwater forcing and signposting the methods section that contains the justification, i.e., that the use of exaggerated freshwater forcing is valid due to AMOC's over-stability in GCMs. I think this point is well-argued, and a quick acknowledgment would be useful, as the freshwater volumes are quite striking on first read, and I assume would be for most paleoceanographers.

We agree that this is a reasonable suggestion. We have added the following note: "These forcing scenarios range from a realistic estimate (i.e., NP01) to an exaggerated value (i.e., NP05). An exaggeration of freshwater forcing is often needed to account for the known AMOC over-stability in current-generation general circulation models (see Methods)."

L61: Consider using 'hinges' instead of 'would hinge'.
Thank you. The change is made.

L137: The phrase/argument '...by proxy evidence' could be strengthened by highlighting the spatial extent of the proxy compilation. It's not just a couple of sites; it's much more impressive than that, e.g., ...by proxy evidence from multiple sites in the Pacific, Indonesian Throughflow and south of Africa.
Thank you for this suggestion. We have made the suggested change.

Reviewer #4 (Remarks to the Author):

I co-reviewed this manuscript with one of the reviewers who provided the listed reports. This is part of the Nature Communications initiative to facilitate training in peer review and to provide appropriate recognition for Early Career Researchers who co-review manuscripts.
We thank Reviewer #4 for their contribution and participating in this initiative.

Response to the reviewers' comments:

Reviewer #1 (Remarks to the Author):

This is my third review of "Triggering of Heinrich events by Cordilleran Ice Sheet meltwater into the North Pacific" by Sun and others. I thank the authors for incorporating my comments into the revised manuscript, including changing the title. I only have one more comment:

We sincerely thank Reviewer #1 for their constructive feedback throughout the three rounds of review. We greatly appreciate the time and effort they devoted to improving the manuscript.

Line 74-75:"these mechanisms are more relevant for Heinrich stadials at glacial terminations or inceptions rather than all Heinrich stadials…" I do not agree with this statement. A number of recent studies have examined DO-type oscillations during MIS 3, when climate conditions were intermediate, for example Zhang et al. (2021, NG) and Vettoretti et al. (2022, NG). Please revise this sentence.

Reference:

Zhang, X. et al. Direct astronomical influence on abrupt climate variability. Nat. Geosci. 14, 819–826 (2021).

Vettoretti, G., Ditlevsen, P., Jochum, M. & Rasmussen, S. O. Atmospheric CO2 control of spontaneous millennial-scale ice age climate oscillations. Nat. Geosci. 15, 300–306 (2022).

We agree with reviewer #1 that CO2- and orbitally induced AMOC oscillations are not restricted to glacial terminations or inceptions. We thank the reviewer for the helpful references and have incorporated the theses reference into the revised manuscript. We have also removed the statement in question. This change does not alter the flow or main arguments of the paper.
